# 1,2-Silyl Rearrangement in Gold Carbene Chemistry: Synthesis of Furyl-Decorated Tetrasubstituted Silylallene Derivatives

**DOI:** 10.1021/acs.orglett.4c01468

**Published:** 2024-05-30

**Authors:** Patricia García-Martínez, Luis A. López

**Affiliations:** Departamento de Química Orgánica e Inorgánica, Instituto Universitario de Química Organometálica “Enrique Moles” and Centro de Innovación en Química Avanzada (ORFEO−CINQA), Universidad de Oviedo, 33006-Oviedo, Spain

## Abstract

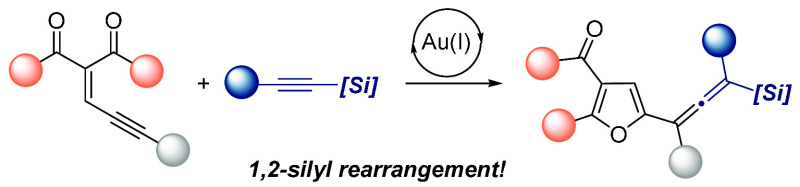

The gold-catalyzed reaction of 2-en-4-ynones with alkynylsilanes provides fully substituted allene derivatives bearing furyl and silyl groups. This transformation would involve generation of a gold furyl carbene intermediate, which regioselectively undergoes a nucleophilic attack by the alkynylsilane at the electrophilic carbene carbon atom with the formation of a β-gold vinyl cation species. The subsequent release of the gold catalyst, accompanied by a 1,2-silyl shift, leads to the formation of tetrasubstituted allene products.

In recent years, there has been significant advancement in gold carbene chemistry, thus becoming a valuable tool in organic synthesis.^1^ This is in part due to a broad reactivity profile as a consequence of the right balance between conventional carbene-like behavior and the atypical reactivity displayed by gold carbene intermediates. This distinctive reactivity, often attributed to their gold-coordinated carbocation-like character,^2^ has been extensively studied, leading to various gold-catalyzed transformations with remarkable efficiency and selectivity. Notwithstanding the wide applicability of gold carbene intermediates, some substrates remain underexplored as trapping reagents in gold carbene chemistry. This is the case of alkynes with only a few examples of capture of gold carbenes with these unsaturated substrates.^3^ In this context, in 2021, our group reported a gold-catalyzed reaction involving propargyl esters and alkynylsilanes, yielding vinylallene derivatives (Scheme 1A).^4^ Mechanistically, this transformation would involve: (1) generation of a gold carbene intermediate thorough [1,2]-acyloxy rearrangement, (2) regioselective C–C bond formation by nucleophilic attack of the alkynylsilane to the carbene carbon with formation of a cationic intermediate, and (3) concurrent gold elimination/[1,2]-silyl rearrangement.

**Scheme 1:**
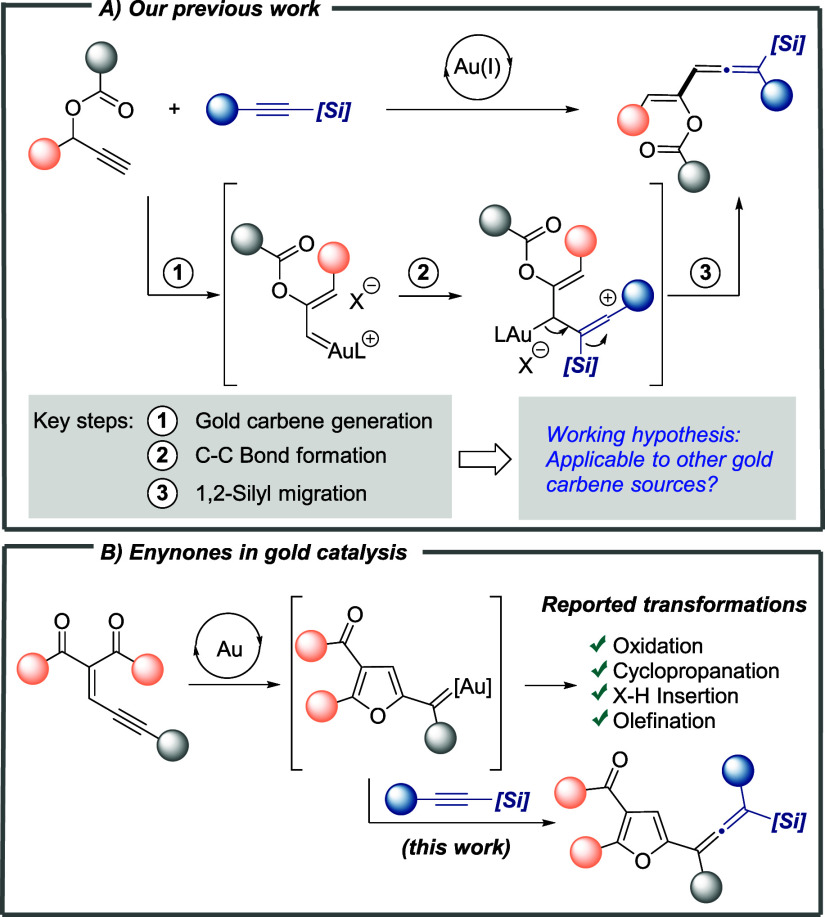
(A) Previous Work and Hypothesis; (B) State-of-the-Art in Gold-Catalyzed Transformations of Enynones

On the other hand, in recent years, enynones have gained increasing attention as nondiazo carbene precursors. In the presence of a suitable transition metal catalyst, these easily available substrates can generate furyl-substituted metal carbene intermediates, which undergo a range of carbene-transfer transformations.^5^ While less explored than other transition metals, some gold complexes were found to be suitable catalysts for the transformation of enynones (Scheme 1B). In 2010, Zhang and co-worker reported the reaction of enynones with H_2_O_2_ in the presence of a catalytic amount of AuCl_3_, leading to 2-acylfurans.^6^ Later on, Zhu and co-workers communicated that a combination of IPrAuCl and Selecfluor is a highly efficient catalytic system for cyclopropanation and X–H (X = O, N, Si) insertion reactions even at very low catalyst loading.^7^ More recently, Sun and co-workers also reported the stereoselective synthesis of 2-vinylfuran derivatives by gold(I)-catalyzed coupling of enynones with diazo reagents.^8^ However, the trapping of furyl-substituted gold carbene intermediates generated from enynones with alkynes has not been previously reported.^9^

Motivated by elegant research on gold-catalyzed transformations of enynones and our current interest in exploring new applications of alkynylsilanes in gold carbene chemistry, we investigated the feasibility of extending the nucleophilic attack/[1,2]-silyl rearrangement sequence observed in gold carbene intermediates from propargyl esters to those from enynones. Herein, we report that the proposed sequence is also operative for furyl-substituted gold carbene intermediates, providing furyl-decorated tetrasubstituted silylallene derivatives (Scheme 1B).

At the outset, we evaluated the performance of several transition-metal catalysts (5 mol %) in the reaction of enynone **1a** and alkynylsilane **2a** in 1,2-dichloroethane (DCE) as the solvent (Table 1).

**Table 1:**
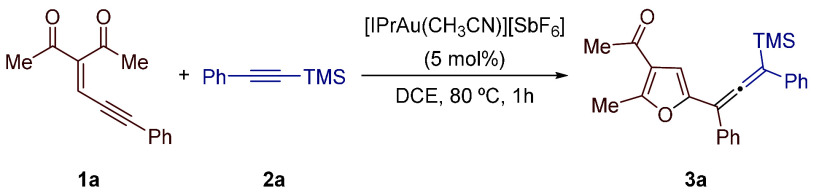
Optimization of Reaction Conditions^a^

entry	Variation from optimal conditions	yield^b^
1	no changes	70%
2	IPrAuNTf_2_ as the catalyst	30%
3	[JohnPhosAu(MeCN)][SbF_6_] as the catalyst	21%
4	Ph_3_PAuCl/AgBF_4_ as the catalyst	16%
5	XPhosAuCl/AgBF_4_ as the catalyst	13%
6^c^	[(2,4-^t^Bu_2_C_6_H_3_O)_3_Au][NTf_2_] as the catalyst	25%
7^d^	IPrAuCl/Selectfluor as the catalyst	traces
8^e^	ZnCl_2_ as the catalyst	2%
9^f^	reaction performed at rt	28%
10	reaction performed at 60 °C	56%
11	1.5 equiv of alkynylsilane	35%
12	10 mol % of the catalyst	55%
13^g^	CH_2_Cl_2_ as the solvent	33%
14	CHCl_3_ as the solvent	21%
15	toluene, THF, acetonitrile as the solvent	–
16^h^	no catalyst	–

a Reaction conditions: enynone **1a** (0.2 mmol), alkynylsilane **2a** (1.0 mmol, 5 equiv), DCE (1 mL), 80 °C, 1 h.

b Isolated yields.

c Reaction time: 90 min.

d Reaction time: 24 h.

e The dimeric alkene resulting from the homocoupling of the enynone was the major product after 24 h at rt.

f Reaction time: 75 h.

g Reaction run at rt.

h Reaction time: 75 h.

As shown, various gold catalysts proved capable of promoting the formation of the desired allene **3a** validating our hypothesis about the feasibility of the proposed sequence (entries 1–6). Among them, [IPrAu(CH_3_CN)][SbF_6_] in DCE at 80 °C outperformed other gold(I) catalysts tested delivering **3a** in 70% yield (entry 1). Although, as stated before,^7^ the use of a combination of IPrAuCl and Selectfluor proved to be extremely useful in cyclopropanation and insertion reactions, it is not a suitable catalytic system for the present transformation (entry 7). Likewise, while ZnCl_2_ was able to catalyze the cyclopropenation of several alkynes using enynones as the carbene source,^8^ it failed to promote the reaction of enynone **1a** and alkynylsilane **2a** (entry 8). Using [IPrAu(CH_3_CN)][SbF_6_] as the catalyst, we found that lower temperatures had a negative impact on the yield of **3a** (entries 9 and10), as did the use of just 1.5 equiv of the alkynylsilane reagent (entry 11). The use of 10 mol % of the catalyst did not translate into an increase of the yield of the desired product (entry 12). On the other hand, conducting the model reaction in CH_2_Cl_2_ or CHCl_3_ provided **3a** in lower yields (entries 13 and 14). In contrast, toluene, THF, and CH_3_CN were not viable solvents for the current transformation (entry 15). Not surprisingly, no reaction was observed at all in the absence of the gold catalyst (entry 16).

With suitable reaction conditions for the model reaction, we next investigated the substrate scope using various enynones **1** and alkynylsilanes **2** (Table 2). First, with enynone **1a** (R^1^ = Me; R^2^ = Ph) as the reaction partner, we investigated the variation of the alkynylsilane component **2**. In this regard, we were pleased to find that several 1-aryl-2-trimethylsilylacetylenes **2** performed satisfactorily in the current transformation. For example, we found that *para*-substituted aryl alkynylsilanes containing methyl and methoxy groups worked well, furnishing the respective products in good yields (**3b**, 73%; **3c**, 60%). Silylallene derivatives **3d**–**3f** bearing *p*-halophenyl groups were also obtained in acceptable yields (40–74%) when using the corresponding alkynylsilanes. Under the developed reaction conditions, an aryl alkynylsilane bearing an electron withdrawing *p*-CF_3_ group could also engage in the reaction with **1a** albeit a lower yield (25%) of the corresponding product **3g** was achieved. It was found that *meta* substitution on the aryl group of the alkynylsilane is not particularly problematic as revealed by the formation of allene **3h** in 62% yield. In contrast, a significant erosion of the yield was observed with an alkynylsilane bearing an *ortho* substituted aryl group as demonstrated by the isolation of compound **3i** in 38% yield. A thienyl-substituted alkynylsilane provided the corresponding allene **3j** in 40% yield. Besides aryl-substituted alkynylsilanes, substrates bearing cyclohexenyl and cyclopropyl substituentes were also amenable reagents providing vinylallenes **3k** and **3l** in 63% and 48% yield, respectively. Given that the gold-catalyzed vinylallene/cyclopentadiene isomerization has been reported by Toste and co-workers,^10^ the isolation of allene **3k** containing a vinylallene framework is remarkable.

**Table 2:**
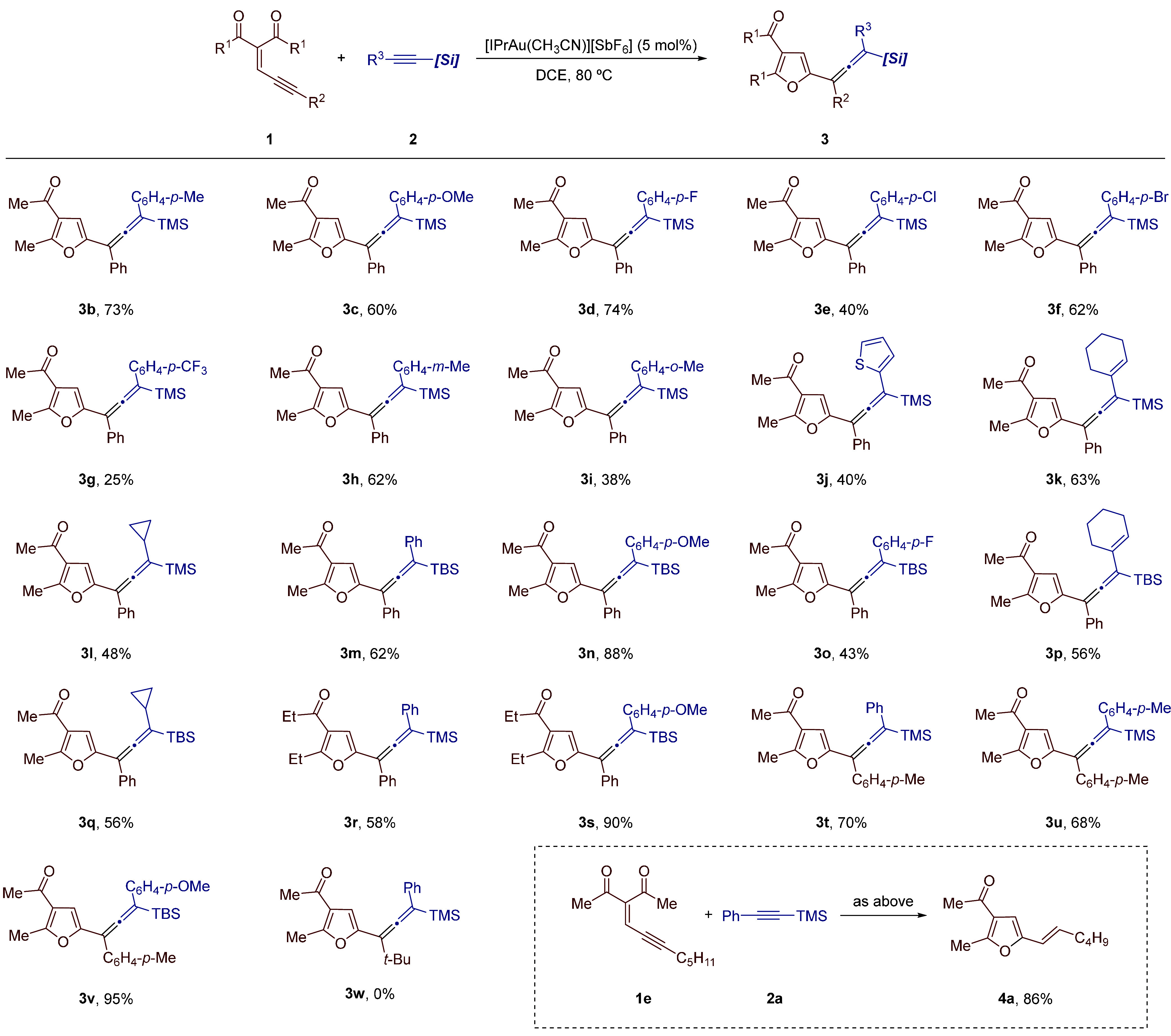
Synthesis of Furyl-Decorated Tetrasubstituted Silylallenes **3**: Scope^a^^,^^b^

a Reaction conditions: enynone **1** (0.2 mmol), alkynylsilane **2** (1.0 mmol, 5 equiv), [(IPr)Au(CH_3_CN)]SbF_6_ (5.0 mol %), DCE (1 mL), 80 °C.

b Yield of isolated products.

Alkynylsilanes bearing a *tert*-butyldimethylsilyl (TBS) group are also able to engage in this gold-catalyzed transformation.^11^ Indeed, under the developed conditions, reaction of enynone **1a** with several TBS-substituted alkynylsilanes containing aryl, cyclohexenyl, and cycloalkyl groups delivered the corresponding vinylallene derivatives **3m**–**3q** in moderate to good yields (43–88%).

Regarding the enynone component, we first demonstrated that enynone **1b** (R^1^ = Et; R^2^ = Ph) arising from 3,5-heptadione also proved to be an effective substrate delivering the corresponding allene derivatives **3r** and **3s** in 58% and 90% yield, respectively. Variation of the aryl group in the enynone was also possible as illustrated by the synthesis of the coupling products **3t**–**3v** in good to excellent yield (68–95%), when using enynone **1c** (R^1^ = Me; R^2^ = *p*-CH_3_C_6_H_4_) in combination with different aryl-substituted alkynylsilanes.

In contrast, enynones substituted at the alkyne terminus with alkyl groups were not suitable substrates for this reaction. For example, reaction of enynone **1d** (R^1^ = Me; R^2^ = *t*-Bu) and alkynylsilane **2a** did not afford the expected furyl-substituted silylallene **3w** and the starting reagents were recovered unchanged. On the other hand, subjecting a mixture of enynone **1e** (R^1^ = Me; R^2^ = C_5_H_11_) and alkynylsilane **2a** to the standard reaction conditions did not provide the expected tetrasubstituted silylallene derivative; instead, 2-vinylfuran derivative **4a** was isolated in 86% yield as the only reaction product (Table 2, dotted box).

Based on our previous findings^4^ and related precedents in gold-catalyzed transformations of enynones,^6−8^ a reasonable catalytic cycle for the formation of furyl-substituted allene derivatives **3** is shown in Scheme 2.^12^ First, coordination of enynone **1** to the gold catalyst followed by 5-*exo*-dig cyclization would generate gold furyl carbene intermediate **I**. Then, the cationic intermediate **II** would result from the attack of the alkynylsilane to the electrophilic carbon of carbene intermediate **I**. Very likely, the stability of cationic species **II** provided by the β-silyl effect would dictate the regioselectivity course of this carbon–carbon bond forming step. Elimination of the gold fragment in intermediate **II** with concurrent 1,2-silyl migration would provide the final product.^13,14^

**Scheme 2:**
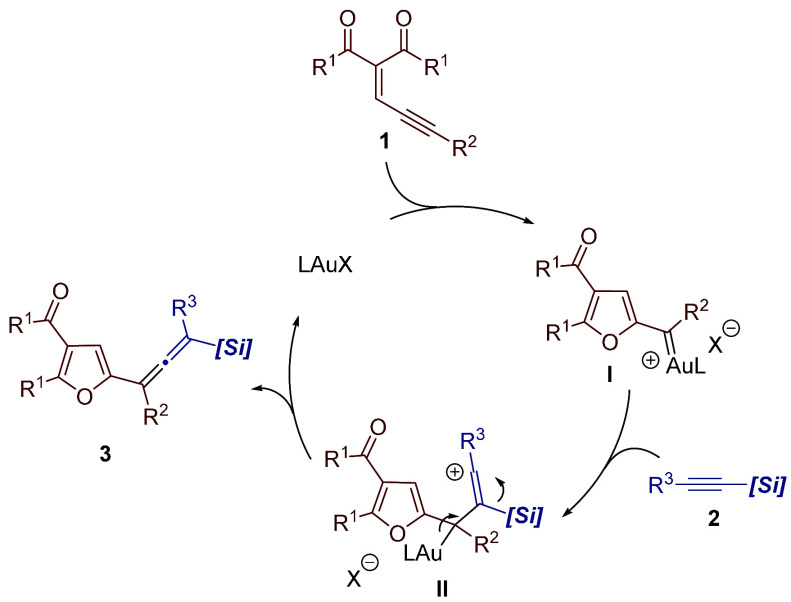
Plausible Mechanism for the Formation of Allenes **3** from Enynones **1** and Alkynylsilanes **2**

Competitive 1,2-H migration in the corresponding gold furyl carbene intermediate **I** would account for the formation of vinylfuran **4a** in the reaction of alkyl-substituted enynone **1e** (see Table 2, dotted box).^15^

Finally, to determine if the proposed gold carbene intermediate **I** could be trapped by alkynes lacking the silyl group, we performed the reaction of enynone **1a** and diphenylacetylene (**2s**). Heating both reagents in DCE at 60 °C in the presence of 5 mol % of [IPrAu(CH_3_CN)][SbF_6_] provided the furyl-substituted indene derivative **5** in low yield (18%) (Scheme 3).^16^ This outcome highlighted the crucial role of the silyl substituent in the successful trapping of gold carbene intermediates generated from enynones.

**Scheme 3:**
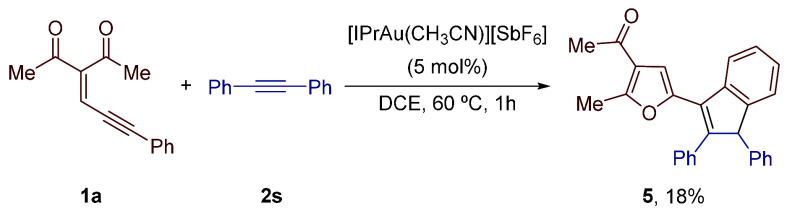
Reaction of Enynone **1a** and Diphenylacetylene (**2s**)

In summary, we have reported a convenient approach to furyl-decorated tetrasubstituted silylallene derivatives based on the gold-catalyzed reaction of enynones and alkynylsilanes. In this transformation, the enynone component would serve as a precursor of a furyl gold carbene intermediate, which would mimic the behavior previously reported for those generated from propargyl esters. Overall, our study highlights the potential of combining the reactivity of gold carbene intermediates with alkynylsilanes for the synthesis of complex allene derivatives. Further exploration of this concept is currently underway in our group.

## Data Availability

The data underlying this study are available in the published article and its Supporting Information.

## Supporting Information Available

Experimental procedures and characterization data for all new compounds (PDF)
