# Supplementary material for: 1,2-Silyl Rearrangement in Gold Carbene Chemistry: Synthesis of Furyl-Decorated Tetrasubstituted Silylallene Derivatives
Source: Org Lett. 2024 May 30;26(22):4695–9. doi: 10.1021/acs.orglett.4c01468 (PMC11165586; doi:10.1021/acs.orglett.4c01468)

Supporting Information

1,2-Silyl Rearrangement in Gold Carbene Chemistry: Synthesis of Furyl-Decorated Tetrasubstituted Silylallene Derivatives

Patricia García-Martínez,[a] Luis A. López[a]*

[a] Departamento de Química Orgánica e Inorgánica, Instituto Universitario de Química Organometálica "Enrique Moles" and Centro de Innovación en Química Avanzada (ORFEO-CINQA), Universidad de Oviedo, Julián Clavería 8, 33006-Oviedo (Spain)

lalg@uniovi.es

Table of Contents

1. General Considerations S-3

2. Optimization of Reaction Conditions S-4

3. General Procedure for the Synthesis of Tetrasubstituted Allenes **3** S-5

4. Characterization Data of Allene Derivatives **3** S-6

5. Synthesis of (*E*)-1-[5-(hex-1-en-1-yl)-2-methylfuran-3-yl]ethan-1-one (**4a**) S-20

6. Experimental Procedure for the Synthesis of **3a** (2.5 mmol Scale) S-21

7. Synthesis of Indene Derivative **5** S-22

8. References S-23

9. NMR Spectra for New Compounds S-24

1. General Considerations

Reactions were performed in a RR9803012 place Carousel Reaction Station™ from Radleys Discovery Technologies, equipped with gastight threaded caps with a valve, cooling reflux head system, and digital temperature controller. All reactions were carried under an atmosphere of nitrogen (99.99%). 1,2-Dichloroethane (DCE) was distilled from CaH_2 before use. The solvents used in column chromatography were obtained from commercial suppliers and used without further distillation. TLC was performed on aluminum-backed plates coated with silica gel 60 with F254 indicator (Merck), using UV light as a visualizing agent and phosphomolybdic acid in ethanol, potassium permanganate solution or *p*-anisaldehyde in ethanol, and heat as developing agent. Flash chromatography was performed on silica gel (40-60 um). ^{1}H NMR (300, 400 MHz) and ^{13}C NMR (75.5, 100 MHz) spectra were measured in $CDCl_3$ at room temperature on a Bruker DPX-300, Bruker AV-300 MHz and Bruker AV-400 instruments, with $CDCl_3$ (δ = 7.26, ^{1}H NMR; δ = 77.16, ^{13}C NMR) as internal standard. Carbon multiplicities were assigned by DEPT techniques.

High-resolution mass spectra (HRMS) were determined by Universidad de Oviedo with a Bruker Impact II, Q – TOF mass Spectrometer.

This study was carried out using enynones **1a**-**e** and alkynylsilanes **2a**-**r** (Figure S1). Enynones **1** were prepared according to literature procedures.[1] Alkynylsilanes **2** were prepared according to literature procedures.[2] [IPrAu(CH_3CN)]SbF_6 was prepared according to a method reported by Nolan and coworkers.[3] All other reagents used in this work were of the best commercial grade available and used without further purification.

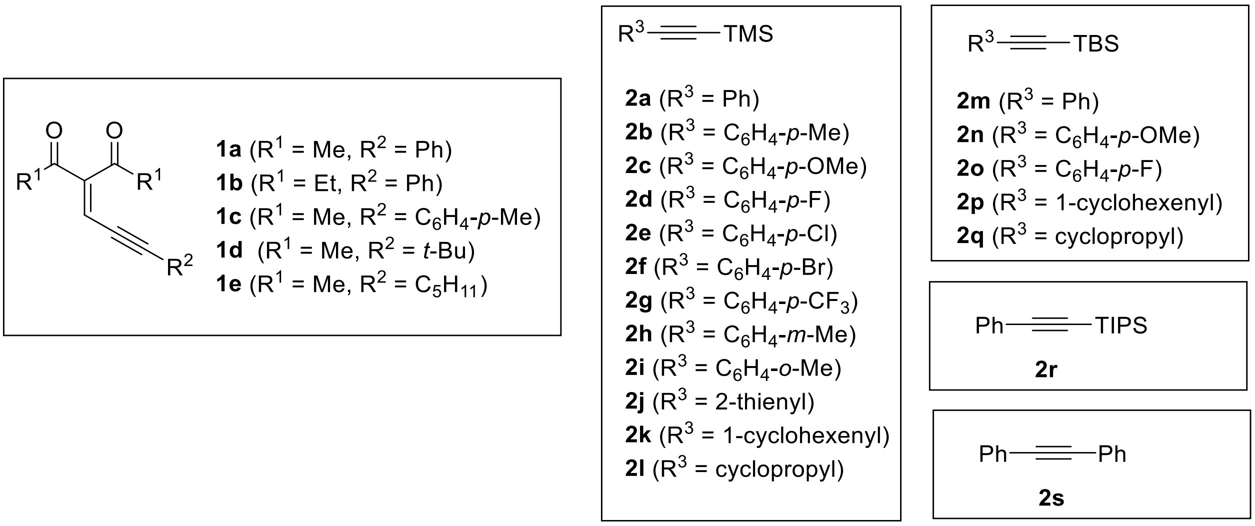

Figure S1. Starting materials used in this work

2. Optimization of Reaction Conditions

Table S1. Reaction of Enynone **1a** and Alkynylsilane **2a**: Optimization of Reaction Conditions[a]

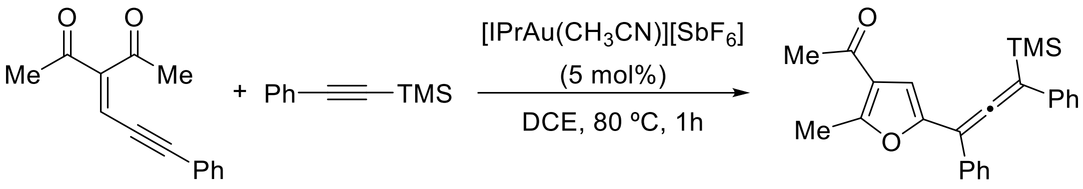

1a **2a** **3a**

entry	Variation from optimal conditions	**3a** (%)[b]
1	no changes	70
2	$IPrAuNTf_2$ used instead	30
3	$[JohnPhosAu(MeCN)][SbF_6]$ used instead	21
4	$Ph_3PAuCl/AgBF_4$ used instead	16
5	$XPhosAuCl/AgBF_4$ used instead	13
6[c]	$[(2,4\text{-}^tBu_2C_6H_3O)_3Au][NTf_2]$ used instead	25
7[d]	IPrAuCl/Selectfluor used instead	traces
8[e]	$ZnCl_2$ used instead	2
9[f]	reaction performed at rt	28
10	reaction performed at 60 ºC	56
11	1.5 equivalents of alkynylsilane	35
12	10 mol% of the catalyst	55
13[g]	CH_2Cl_2 as the solvent	33
14	$CHCl_3$ as the solvent	21
15	Toluene as the solvent	-
16	THF as the solvent	-
17	Acetonitrile as the solvent	-
18[h]	no catalyst	-

[a] Reaction conditions: **1a** (0.2 mmol), **2a** (1.0 mmol, 5 equiv), DCE (1 mL), 80 ºC, 1h. [b] Isolated yields after column chromatography (silica gel; hexanes/ethyl acetate 10:1). [c] Reaction time: 90 min. [d] Reaction time: 24 h. [e] The dimeric alkene resulting from the homocoupling of the enynone was the major product after 24 h at rt. [f] Reaction time: 75 h. [g] Reaction performed at rt. [h] Reaction time: 75 h.

3. General Procedure for the Synthesis of Tetrasubstituted Allenes 3

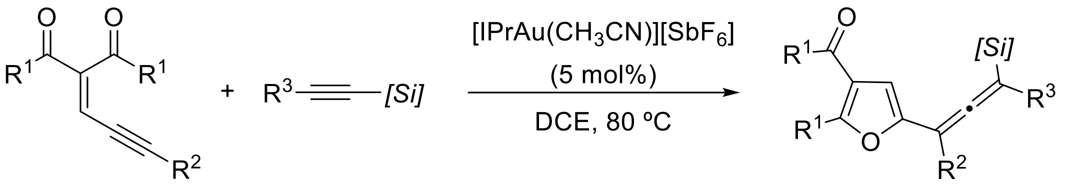

1 **2** **3**

$[(IPr)Au(CH_3CN)]SbF_6$ (8.6 mg, 5.0 mol%) was added to a solution of enynone **1** (0.20 mmol) and alkynylsilane **2** (1.0 mmol, 5 equiv) in DCE (1 mL). The resulting mixture was stirred at 80 ºC until disappearance of the starting propargyl ester (checked by TLC, 0.5-1.0 h). Then, the solvent was removed under reduced pressure and the resulting mixture was purified by flash chromatography (silica gel, hexanes/ethyl acetate 10:1) to yield allenes **3**.

4. Characterization Data of Allene Derivatives 3

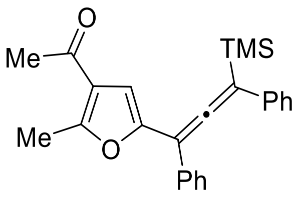

3a

1-{5-[1,3-diphenyl-3-(trimethylsilyl)propa-1,2-dien-1-yl]-2-methylfuran-3-yl}ethan-1-one (3a)

Allene **3a** was obtained from enynone **1a** (42.5 mg, 0.20 mmol) and alkynylsilane **2a** (174.3 mg, 1.0 mmol, 5 equiv.). After 1 h, purification by flash chromatography (silica gel, hexanes/ethyl acetate 10:1) afforded compound **3a** (54.1 mg, 70% yield) as a yellow oil.

^{1}H NMR (300 MHz, $CDCl_3$): δ = 7.48-7.24 (m, 10H), 6.47 (s, 1H), 2.61 (s, 3H), 2.39 (s, 3H), 0.32 (s, 9H) ppm.

^{13}C NMR (75 MHz, $CDCl_3$): δ = 209.2 (C), 194.2 (C), 158.0 (C), 147.7 (C), 136.3 (C), 134.9 (C), 128.83 (CH), 128.80 (CH), 128.2 (CH), 127.8 (CH), 127.6 (CH), 127.1 (CH), 123.2 (C), 108.2 (CH), 105.8 (C), 98.5 (C), 29.3 (CH_3), 14.7 (CH_3), -0.1 (CH_3) ppm.

HRMS (EI) m/z: $[M + H]^+$ Calcd for $C_{25}H_{27}O_2Si$ 387.1775; Found 387.1775.

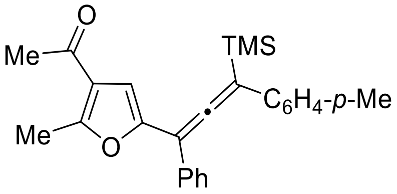

3b

1-{2-methyl-5-[1-phenyl-3-(*p*-tolyl)-3-(trimethylsilyl)propa-1,2-dien-1-yl]furan-3-yl}ethan-1-one (3b)

Allene **3b** was obtained from enynone **1a** (42.5 mg, 0.20 mmol) and alkynylsilane **2b** (188.3 mg, 1.0 mmol, 5 equiv.). After 1 h, purification by flash chromatography (silica gel, hexanes/ethyl acetate 10:1) afforded compound **3b** (58.5 mg, 73% yield) as a yellow oil.

^{1}H NMR (300 MHz, $CDCl_3$): δ = 7.51-7.32 (m, 7H), 7.19 (d, J = 9.0 Hz, 2H), 6.50 (s, 1H), 2.64 (s, 3H), 2.42 (s, 3H), 2.38 (s, 3H), 0.34 (s, 9H) ppm.

^{13}C NMR (75 MHz, $CDCl_3$): δ = 209.0 (C), 194.3 (C), 158.0 (C), 147.8 (C), 136.9 (C), 134.9 (C), 133.0 (C), 129.6 (CH), 128.8 (CH), 128.1 (CH), 127.7 (CH), 127.5 (CH), 123.2 (C), 108.0 (CH), 105.4 (C), 98.5 (C), 29.3 (CH_3), 21.3 (CH_3), 14.7 (CH_3), -0.1 (CH_3) ppm.

HRMS (EI) m/z: $[M + H]^+$ Calcd for $C_{26}H_{29}O_2Si$ 401.1931; Found 401.1931.

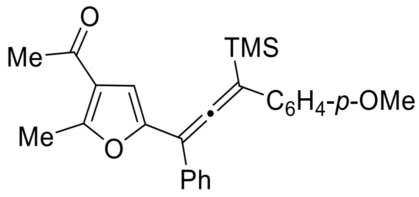

3c

1-{5-[3-(4-methoxyphenyl)-1-phenyl-3-(trimethylsilyl)propa-1,2-dien-1-yl]-2-methylfuran-3-yl}ethan-1-one (3c)

Allene **3c** was obtained from enynone **1a** (42.5 mg, 0.20 mmol) and alkynylsilane **2c** (204.3 mg, 1.0 mmol, 5 equiv.). After 0.5 h, purification by flash chromatography (silica gel, hexanes/ethyl acetate 10:1) afforded compound **3c** (50.0 mg, 60% yield) as a yellow oil.

^{1}H NMR (300 MHz, $CDCl_3$): δ = 7.54-7.26 (m, 7H), 6.91 (d, *J* = 8.7 Hz, 2H), 6.49 (s, 1H), 3.83 (s, 3H), 2.63 (s, 3H), 2.41 (s, 3H), 0.33 (s, 9H) ppm.

^{13}C NMR (75 MHz, $CDCl_3$): δ = 208.8 (C), 194.3 (C), 158.8 (C), 157.9 (C), 147.9 (C), 135.0 (C), 129.3 (CH), 128.8 (CH), 128.1 (C), 127.7 (CH), 127.5 (CH), 123.2 (C), 114.3 (CH), 108.0 (CH), 105.0 (C), 98.5 (C), 55.5 (CH_3), 29.3 (CH_3), 14.8 (CH_3), 0.0 (CH_3) ppm.

HRMS (EI) m/z: $[M + H]^+$ Calcd for $C_{26}H_{29}O_3Si$ 417.1880; Found 417.1884.

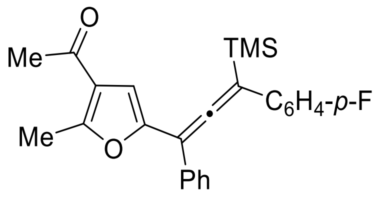

3d

1-{5-[3-(4-fluorophenyl)-1-phenyl-3-(trimethylsilyl)propa-1,2-dien-1-yl]-2-methylfuran-3-yl}ethan-1-one (3d)

Allene **3d** was obtained from enynone **1a** (42.5 mg, 0.20 mmol) and alkynylsilane **2d** (192.3 mg, 1.0 mmol, 5 equiv.). After 1 h, purification by flash chromatography (silica gel, hexanes/ethyl acetate 10:1) afforded compound **3d** (59.9 mg, 74% yield) as a yellow oil.

^{1}H NMR (300 MHz, $CDCl_3$): δ = 7.51-7.31 (m, 7H), 7.09-7.03 (m, 2H), 6.50 (s, 1H), 2.65 (s, 3H), 2.42 (s, 3H), 0.34 (s, 9H) ppm.

^{13}C NMR (75 MHz, $CDCl_3$): δ = 209.0 (C), 194.2 (C), 162.0 (C, J_{C-F} = 245.3 Hz), 158.0 (C), 147.5 (C), 134,7 (C), 132.1 (C, J_{C-F} = 2.3 Hz),129.6 (CH, J_{C-F} = 7.5 Hz), 128.8 (CH), 127.7 (CH), 127.6 (CH), 123.2 (C), 115.7 (CH, J_{C-F} = 21.8 Hz), 108.3 (CH), 104.9 (C), 98.6 (C), 29.3 (CH_3), 14.7 (CH_3), -0.2 (CH_3) ppm.

^{19}F NMR (282 MHz, $CDCl_3$): δ = -115.4 ppm.

HRMS (EI) m/z: $[M + Na]^+$ Calcd for $C_{25}H_{25}FNaO_2Si$ 427.1500; Found 427.1523.

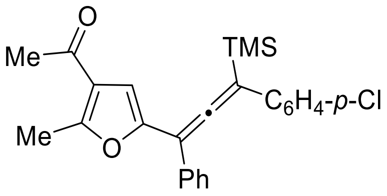

3e

1-{5-[3-(4-chlorophenyl)-1-phenyl-3-(trimethylsilyl)propa-1,2-dien-1-yl]-2-methylfuran-3-yl}ethan-1-one (3e)

Allene **3e** was obtained from enynone **1a** (42.5 mg, 0.20 mmol) and alkynylsilane **2e** (208.8 mg, 1.0 mmol, 5 equiv.). After 1 h, purification by flash chromatography (silica gel, hexanes/ethyl acetate 10:1) afforded compound **3e** (33.7 mg, 40% yield) as a yellow oil.

^{1}H NMR (300 MHz, $CDCl_3$): δ = 7.48-7.29 (m, 9H), 6.48 (s, 1H), 2.63 (s, 3H), 2.41 (s, 3H), 0.32 (s, 9H) ppm.

^{13}C NMR (75 MHz, $CDCl_3$): δ = 209.3 (C), 194.2 (C), 158.1 (C), 147.4 (C), 134.9 (C), 134.6 (C), 132.9 (C), 129.4 (CH), 129.0 (CH), 128.9 (CH), 127.8 (CH), 127.7 (CH), 123.3 (C), 108.4 (CH), 105.1 (C), 98.8 (C), 29.3 (CH_3), 14.7 (CH_3), 0.2 (CH_3) ppm.

HRMS (EI) m/z: $[M + Na]^+$ Calcd for $C_{25}H_{25}ClNaO_2Si$ 443.1205; Found 443.1209.

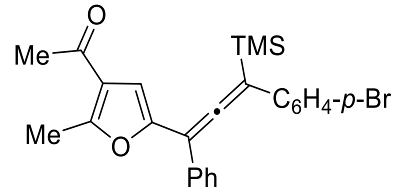

3f

1-{5-[3-(4-bromophenyl)-1-phenyl-3-(trimethylsilyl)propa-1,2-dien-1-yl]-2-methylfuran-3-yl}ethan-1-one (3f)

Allene **3f** was obtained from enynone **1a** (42.5 mg, 0.20 mmol) and alkynylsilane **2f** (253.2 mg, 1.0 mmol, 5 equiv.). After 1 h, purification by flash chromatography (silica gel, hexanes/ethyl acetate 10:1) afforded compound **3f** (57.7 mg, 62% yield) as a yellow oil.

^{1}H NMR (300 MHz, $CDCl_3$): δ = 7.50-7.27 (m, 9H), 6.49 (s, 1H), 2.64 (s, 3H), 2.42 (s, 3H), 0.33 (s, 9H) ppm.

^{13}C NMR (75 MHz, $CDCl_3$): δ = 209.3 (C), 194.2 (C), 158.1 (C), 147.3 (C), 135.3 (C), 134.5 (C), 131.9 (CH), 129.7 (CH), 128.9 (CH), 127.8 (CH), 123.3 (C), 108.4 (CH), 105.1 (C), 98.8 (C), 29.3 (CH_3), 14.7 (CH_3), -0.2 (CH_3) ppm.

HRMS (EI) m/z: $[M + Na]^+$ Calcd for $C_{25}H_{25}BrNaO_2Si$ 487.0699; Found 487.0703.

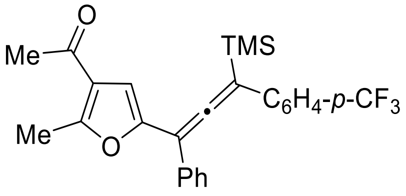

3g

1-{2-methyl-5-[1-phenyl-3-(4-(trifluoromethyl)phenyl)-3-(trimethylsilyl)propa-1,2-dien-1-yl]furan-3-yl}ethan-1-one (3g)

Allene **3g** was obtained from enynone **1a** (42.5 mg, 0.20 mmol) and alkynylsilane **2g** (242.3 mg, 1.0 mmol, 5 equiv.). After 2 h, purification by flash chromatography (silica gel, hexanes/ethyl acetate 10:1) afforded compound **3g** (22.7 mg, 25% yield) as a yellow oil.

^{1}H NMR (300 MHz, $CDCl_3$): δ = 7.50 (d, J = 8.1 Hz, 2H), 7.44-7.13 (m, 7H), 6.38 (s, 1H), 2.53 (s, 3H), 2.30 (s, 3H), 0.23 (s, 9H) ppm.

^{13}C NMR (125 MHz, $CDCl_3$): δ = 209.7 (C), 194.0 (C), 158.1 (C), 146.9 (C), 140.3 (C), 134.1 (C), 128.9 (C, J_{CF} = 32.6 Hz), 128.8 (CH), 128.2 (CH), 127.73 (CH), 127.68 (CH), 125.6 (CH,

J_{CF} = 3.7 Hz), 124.1 (C, J_{CF} = 272.3 Hz), 123.2 (C), 108.5 (CH), 105.2 (C), 98.3 (C), 29.2 (CH_3), 14.6 (CH_3), -0.35 (CH_3) ppm.

^{19}F NMR (282 MHz, $CDCl_3$): δ = - 62.5 ppm.

HRMS (EI) m/z: $[M + H]^+$ Calcd for $C_{26}H_{26}F_3O_2Si$ 455.1649; Found 455.1645.

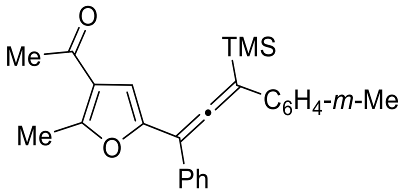

3h

1-{2-methyl-5-[1-phenyl-3-(*m*-tolyl)-3-(trimethylsilyl)propa-1,2-dien-1-yl]furan-3-yl}ethan-1-one (3h)

Allene **3h** was obtained from enynone **1a** (42.5 mg, 0.20 mmol) and alkynylsilane **2h** (188.3 mg, 1.0 mmol, 5 equiv.). After 30 min, purification by flash chromatography (silica gel, hexanes/ethyl acetate 10:1) afforded compound **3h** (49.7 mg, 62% yield) as a yellow oil.

^{1}H NMR (300 MHz, $CDCl_3$): δ = 7.52 (d, *J* = 8.2 Hz, 2H), 7.41 (t, *J* = 7.2 Hz, 2H),7.36-7.21 (m, 4H), 7.13-7.06 (m, 1H), 6.53 (s, 1H), 2.65 (s, 3H), 2.42 (s, 3H), 2.39 (s, 3H), 0.39 (s, 9H) ppm.

^{13}C NMR (75 MHz, $CDCl_3$): δ = 209.0 (C), 194.2 (C), 157.9 (C), 147.7 (C), 138.4 (C), 136.0 (C), 134.8 (C), 128.8 (CH), 128.7 (CH), 128.6 (CH), 127.9 (CH), 127.7 (CH), 127.5 (CH), 125.2 (CH), 123.2 (C), 108.1 (CH), 105.7 (C), 98.4 (C), 29.3 (CH_3), 21.7 (CH_3), 14.7 (CH_3), -0.1 (CH_3) ppm.

HRMS (EI) m/z: $[M + H]^+$ Calcd for $C_{26}H_{29}O_2Si$ 401.1931; Found 401.1918.

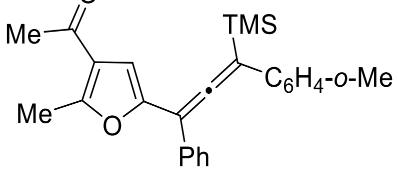

3i

1-{2-methyl-5-[1-phenyl-3-(*o*-tolyl)-3-(trimethylsilyl)propa-1,2-dien-1-yl]furan-3-yl}ethan-1-one (3i)

Allene **3i** was obtained from enynone **1a** (42.5 mg, 0.20 mmol) and alkynylsilane **2i** (188.3 mg, 1.0 mmol, 5 equiv.). After 40 min, purification by flash chromatography (silica gel, hexanes/ethyl acetate 10:1) afforded compound **3i** (30.4 mg, 38% yield) as a yellow oil.

^{1}H NMR (300 MHz, $CDCl_3$): δ = 7.48-7.28 (m, 5H), 7.21-7.09 (m, 4H), 6.41 (s, 1H), 2.63 (s, 3H), 2.38 (s, 3H), 2.28 (s, 3H), 0.21 (s, 9H) ppm.

^{13}C NMR (75 MHz, $CDCl_3$): δ = 205.7 (C), 194.4 (C), 157.7 (C), 148.0 (C), 136.2 (C), 135.5 (C), 134.9 (C), 130.5 (CH), 128.7 (CH), 128.3 (CH), 127.9 (CH), 127.4 (CH), 126.6 (CH), 125.8 (CH), 123.2 (C), 107.9 (CH), 104.7 (C), 96.2 (C), 29.3 (CH_3), 20.8 (CH_3), 14.6 (CH_3), - 0.8 (CH_3) ppm.

HRMS (EI) m/z: $[M + Na]^+$ Calcd for $C_{26}H_{28}NaO_2Si$ 423.1751; Found 423.1753.

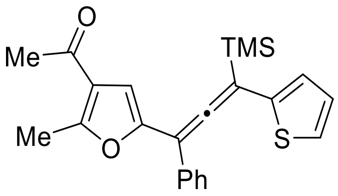

3j

1-{2-methyl-5-[1-phenyl-3-(thiophen-2-yl)-3-(trimethylsilyl)propa-1,2-dien-1-yl]furan-3-yl}ethan-1-one (3j)

Allene **3j** was obtained from enynone **1a** (42.5 mg, 0.20 mmol) and alkynylsilane **2j** (180.3 mg, 1.0 mmol, 5 equiv.). After 45 min, purification by flash chromatography (silica gel, hexanes/ethyl acetate 10:1) afforded compound **3j** (31.4 mg, 40% yield) as a yellow oil.

^{1}H NMR (300 MHz, $CDCl_3$): δ = 7.58-7.51 (m, 2H), 7.48-7.34 (m, 3H), 7.28 (dd, J = 4.9 and 1.5 Hz, 1H), 7.11-7.04 (m, 2H), 6.54 (s, 1H), 2.66 (s, 3H), 2.45 (s, 3H), 0.40 (s, 9H) ppm.

^{13}C NMR (75 MHz, $CDCl_3$): δ = 209.3 (C), 194.2 (C), 158.3 (C), 147.3 (C), 139.5 (C), 128.8 (CH), 128.0 (CH), 127.8 (CH), 127.6 (CH), 125.3 (CH), 125.1 (CH), 123.2 (C), 108.6 (CH), 100.2 (C), 99.4 (C), 29.3 (CH_3), 14.7 (CH_3), 0.3 (CH_3) ppm.

HRMS (EI) m/z: $[M + H]^+$ Calcd for $C_{23}H_{25}O_2SSi$ 393.1339; Found 393.1340.

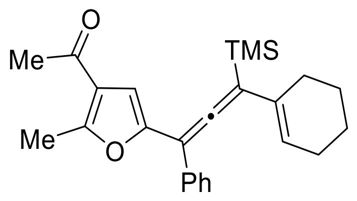

3k

1-{5-[3-(cyclohex-1-en-1-yl)-1-phenyl-3-(trimethylsilyl)propa-1,2-dien-1-yl]-2-methylfuran-3-yl}ethan-1-one (3k)

Allene **3k** was obtained from enynone **1a** (42.5 mg, 0.20 mmol) and alkynylsilane **2k** (178.4 mg, 1.0 mmol, 5 equiv.). After 1 h, purification by flash chromatography (silica gel, hexanes/ethyl acetate 10:1) afforded compound **3k** (49.2 mg, 63% yield) as a yellow oil.

^{1}H NMR (300 MHz, $CDCl_3$): δ = 7.37-7.09 (m, 5H) 6.29 (s, 1H), 5.71 (br s, 1H) 2.49 (s, 3H), 2.27 (s, 3H), 2.10-2.02 (m 4H), 1.63-1.44 (m, 4H), -0.13 (s, 9H) ppm.

^{13}C NMR (75 MHz, $CDCl_3$): δ = 208.3 (C), 194.3 (C), 157.8 (C), 148.5 (C), 135.5 (C), 132.9 (C), 128.7 (CH), 127.5 (CH), 127.3 (CH), 127.2 (CH), 123.1 (C), 107.5 (CH), 98.4 (C), 29.3 (CH_3), 28.9 (CH_2), 26.3 (CH_2), 23.1 (CH_2), 22.3 (CH_2), 14.8 (CH_3), 0.23 (CH_3) ppm.

HRMS (EI) m/z: $[M + Na]^+$ Calcd for $C_{25}H_{30}NaO_2Si$ 413.1907; Found 413.1944.

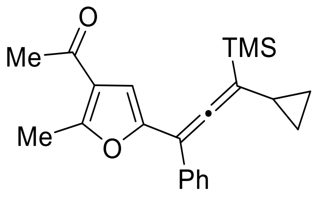

3l

1-{5-[3-(cyclopropyl-1-phenyl-3-(trimethylsilyl)propa-1,2-dien-1-yl]-2-methylfuran-3-yl}ethan-1-one (3l)

Allene **3l** was obtained from enynone **1a** (42.5 mg, 0.20 mmol) and alkynylsilane **2l** (188.3 mg, 1.0 mmol, 5 equiv.). After 1 h, purification by flash chromatography (silica gel, hexanes/ethyl acetate 10:1) afforded compound **3l** (33.7 mg, 48% yield) as a yellow oil.

^{1}H NMR (300 MHz, $CDCl_3$): δ = 7.47-7.24 (m, 5H), 6.38 (s, 1H), 2.61 (s, 3H), 2.40 (s, 3H) 1.34-1.23 (m, 1H), 0.83-0.74 (m, 2H), 0.58-0.54 (m, 2H), 0.24 (s, 9H) ppm.

^{13}**C NMR** (75 MHz, $CDCl_3$): δ = 203.1 (C), 194.3 (C), 157.6 (C), 148.6 (C), 135.6 (C), 128.6 (CH), 127.5 (CH), 127.2 (CH), 123.1 (C), 107.4 (CH), 107.2 (C), 98.9 (C), 29.3 (CH_3), 14.7 (CH_3), 10.1 (CH), 8.7 (CH_2), 8.1 (CH_2), - 1.1 (CH_3) ppm.

HRMS (EI) m/z: $[M + H]^+$ Calcd for $C_{22}H_{27}O_2Si$ 351.1775; Found 351.1773.

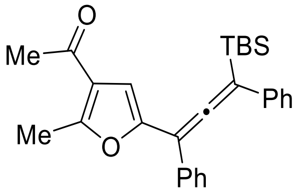

3m

1-{5-[3-(*tert*-butyldimethylsilyl)-1,3-diphenylpropa-1,2-dien-1-yl]-2-methylfuran-3-yl}ethan-1-one (3m)

Allene **3m** was obtained from enynone **1a** (42.5 mg, 0.20 mmol) and alkynylsilane **2m** (216.4 mg, 1.0 mmol, 5 equiv.). After 75 min, purification by flash chromatography (silica gel, hexanes/ethyl acetate 10:1) afforded compound **3m** (53.2 mg, 62% yield) as a yellow oil.

^{1}**H NMR** (300 MHz, $CDCl_3$): δ = 7.36-7.11 (m, 10H), 6.35 (s, 1H), 2.51 (s, 3H), 2.28 (s, 3H), 0.76 (s, 9H) 0.19 (s, 3H), 0.13 (s, 3H) ppm.

^{13}**C NMR** (75 MHz, $CDCl_3$): δ = 210.1 (C), 194.3 (C), 157.9 (C), 147.8 (C), 137.6 (C), 134.9 (C), 128.74 (CH), 128.66 (CH), 128.5 (CH), 127.9 (CH), 127.5 (CH), 126.8 (CH), 123.3 (C), 108.1 (CH), 104.6 (C), 97.6 (C), 29.3 (CH_3), 27.1 (CH_3), 18.3 (C), 14.7 (CH_3), -4.0 (CH_3), -4.2 (CH_3) ppm.

HRMS (EI) m/z: $[M + Na]^+$ Calcd for $C_{28}H_{32}NaO_2Si$ 451.2064; Found 451.2065.

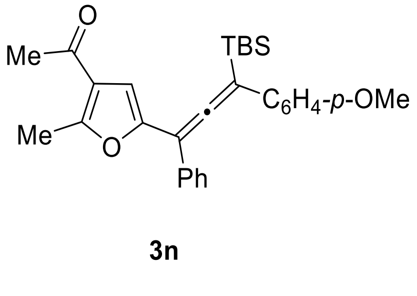

3n

1-{5-[3-(tert-butyldimethylsilyl)-3-(4-methoxyphenyl)-1-phenylpropa-1,2-dien-1-yl]-2-methylfuran-3-yl}ethan-1-one (3n)

Allene **3n** was obtained from enynone **1a** (42.5 mg, 0.20 mmol) and alkynylsilane **2n** (246.4 mg, 1.0 mmol, 5 equiv.). After 50 min, purification by flash chromatography (silica gel, hexanes/ethyl acetate 10:1) afforded compound **3n** (80.7 mg, 88% yield) as a yellow oil.

^{1}H NMR (300 MHz, $CDCl_3$): δ = 7.50 (d, *J* = 7.0 Hz, 2H), 7.44-7.24 (m, 5H), 6.91 (d, *J* = 8.9 Hz, 2H), 6.50 (s, 1H), 3.83 (s, 3H), 2.65 (s, 3H), 2.42 (s, 3H), 0.92 (s, 9H), 0.35 (s, 3H), 0.28 (s, 3H) ppm.

^{13}C NMR (75 MHz, $CDCl_3$): δ = 209.9 (C), 194.2 (C), 158.6 (C), 157.7 (C), 147.9 (C), 135.0 (C), 129.5 (CH), 129.3 (C), 128.7 (CH), 127.8 (CH), 127.4 (CH), 123.1 (C), 114.1 (CH), 107.9 (CH), 103.8 (C), 97.6 (C), 55.3 (CH_3), 29.3 (CH_3), 27.1 (CH_3), 18.2 (C), 14.6 (CH_3), - 4.0 (CH_3), - 4.2 (CH_3) ppm.

HRMS (EI) m/z: $[M + Na]^+$ Calcd for $C_{29}H_{34}NaO_3Si$ 481.2169; Found 481.2170.

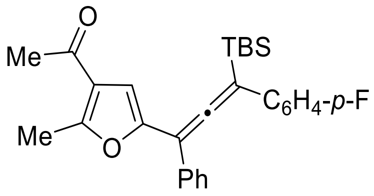

3o

1-{5-[3-(*tert*-butyldimethylsilyl)-3-(4-fluorophenyl)-1-phenylpropa-1,2-dien-1-yl]-2-methylfuran-3-yl}ethan-1-one (3o)

Allene **3o** was obtained from enynone **1a** (42.5 mg, 0.20 mmol) and alkynylsilane **2o** (234.4 mg, 1.0 mmol, 5 equiv.). After 1.5 h, purification by flash chromatography (silica gel, hexanes/ethyl acetate 10:1) afforded compound **3o** (38.4 mg, 43% yield) as a yellow oil.

^{1}H NMR (300 MHz, $CDCl_3$): δ = 7.52-7.23 (m, 7H), 7.01 (t, *J* = 8.7 Hz, 2H), 6.45 (s, 1H), 2.61 (s, 3H), 2.39 (s, 3H), 0.85 (s, 9H) 0.29 (s, 3H), 0.23 (s, 3H) ppm.

^{13}C NMR (75 MHz, $CDCl_3$): δ = 210.1 (C), 194.2 (C), 162.0 (C, $J_{C\text{-}F}$ = 245.8 Hz), 157.9 (C), 147.6 (C), 134,7 (C), 133 (C),129.9 (CH, $J_{C\text{-}F}$ = 7.8 Hz), 128.8 (CH), 127.9 (CH), 127.7 (CH), 123.3 (C), 115.6 (CH, $J_{C\text{-}F}$ = 21.4 Hz), 108.3 (CH), 103.8 (C), 97.8 (C), 29.3 (CH_3), 27.0 (CH_3), 18.3 (C), 14.7 (CH_3), - 4.1 (CH_3) - 4.3 (CH_3) ppm.

^{19}F NMR (282 MHz, $CDCl_3$): δ = -115.9 ppm.

HRMS (EI) m/z: $[M + Na]^+$ Calcd for $C_{28}H_{31}FNaO_2Si$ 469.1970; Found 469.1968.

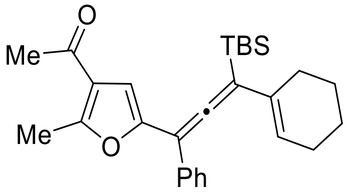

3p

1-{5-[3-(*tert*-butyldimethylsilyl)-3-(cyclohex-1-en-1-yl)-1-phenylpropa-1,2-dien-1-yl]-2-methylfuran-3-yl}ethan-1-one (3p)

Allene **3p** was obtained from enynone **1a** (42.5 mg, 0.20 mmol) and alkynylsilane **2p** (220.4 mg, 1.0 mmol, 5 equiv.). After 1 h, purification by flash chromatography (silica gel, hexanes/ethyl acetate 10:1) afforded compound **3p** (48.5 mg, 56% yield) as a yellow oil.

^{1}H NMR (300 MHz, $CDCl_3$): δ = 7.59-7.24 (m, 5H) 6.42 (s, 1H), 5.86-5.82 (m, 1H) 2.61 (s, 3H), 2.40 (s, 3H), 2.27-2.13 (m 4H), 1.74-1.57 (m, 4H), 0.92 (s, 9H) 0.24 (s, 3H), 0.18 (s, 3H) ppm.

^{13}C NMR (75 MHz, $CDCl_3$): δ = 209.3 (C), 194.3 (C), 157.7 (C), 148.4 (C), 135.5 (C), 133.4 (C), 128.6 (CH), 127.7 (CH), 127.66 (CH), 127.2 (CH), 123.1 (C), 107.5 (CH), 106.0 (C), 98.0 (C), 29.3 (CH_2), 27.4 (CH_3), 26.2 (CH_2), 23.2 (CH_2), 22.2 (CH_2), 18.2 (C), 14.6 (CH_3), - 3.5 (CH_3), - 3.7 (CH_3) ppm.

HRMS (EI) m/z: $[M + H]^+$ Calcd for $C_{28}H_{37}O_2Si$ 433.2557; Found 433.2572.

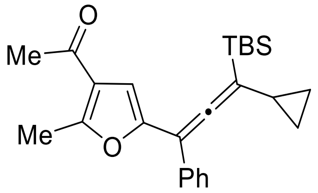

3q

1-{5-[3-(*tert*-butyldimethylsilyl)-3-cyclopropyl-1-phenylpropa-1,2-dien-1-yl]-2-methylfuran-3-yl}ethan-1-one (3q)

Allene **3q** was obtained from enynone **1a** (42.5 mg, 0.20 mmol) and alkynylsilane **2q** (180.4 mg, 1.0 mmol, 5 equiv.). After 40 min, purification by flash chromatography (silica gel, hexanes/ethyl acetate 10:1) afforded compound **3q** (44.0 mg, 56% yield) as a yellow oil.

^{1}H NMR (300 MHz, $CDCl_3$): δ = 7.34-7.09 (m, 5H), 6.24 (s, 1H), 2.47 (s, 3H), 2.26 (s, 3H), 1.21-1.08 (m, 1H), 0.85 (s, 9H) 0.75-0.62 (m, 2H), 0.57-0.51 (m, 2H), 0.08 (s, 3H), 0.04 (s, 3H) ppm.

^{13}C NMR (100 MHz, $CDCl_3$): δ = 203.9 (C), 194.3 (C), 157.5 (C), 148.6 (C), 135.6 (C), 128.6 (CH), 127.6 (CH), 127.2 (CH), 123.1 (C), 107.4 (CH), 105.6 (C), 99.1 (C), 29.2 (CH_3), 26.8 (CH_3), 17.8 (C), 14.6 (CH_3), 10.8 (CH), 9.9 (CH_2), 9.2 (CH_2), -5.4 (CH_3), -5.6 (CH_3) ppm.

HRMS (EI) m/z: $[M + H]^+$ Calcd for $C_{25}H_{33}O_2Si$ 393.2244; Found 393.2251.

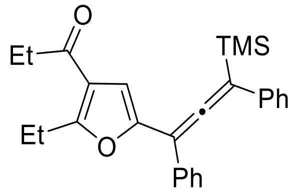

3r

1-{5-[1,3-diphenyl-3-(trimethylsilyl)propa-1,2-dien-1-yl]-2-ethylfuran-3-yl}propan-1-one (3r)

Allene **3r** was obtained from enynone **1b** (48.1 mg, 0.20 mmol) and alkynylsilane **2a** (174.3 mg, 1.0 mmol, 5 equiv.). After 1 h, purification by flash chromatography (silica gel, hexanes/ethyl acetate 10:1) afforded compound **3r** (48.1 mg, 58% yield) as a yellow oil.

^{1}H NMR (300 MHz, $CDCl_3$): δ = 7.71-7.19 (m, 10H), 6.51 (s, 1H), 3.22-2.96 (m, 2H), 2.76 (q, *J* = 7.3 Hz, 2H), 1.31 (t, *J* = 7.5 Hz, 3H), 1.19 (t, *J* = 7.3 Hz, 3H), 0.35 (s, 9H) ppm.

^{13}C NMR (75 MHz, $CDCl_3$): δ = 209.2 (C), 197.2 (C), 162.6 (C), 147.5 (C), 136.4 (C), 134.9 (C), 128.8 (CH), 128.77 (CH), 128.2 (CH), 127.8 (CH), 127.5 (CH), 127.0 (CH), 121.7 (C), 107.7 (CH), 105.8 (C), 98.6 (C), 34.5 (CH_2), 21.9 (CH_2), 12.1 (CH_3), 8.0 (CH_3), -0.2 (CH_3) ppm.

HRMS (EI) m/z: $[M + Na]^+$ Calcd for $C_{27}H_{30}NaO_2Si$ 437.1907; Found 437.1916.

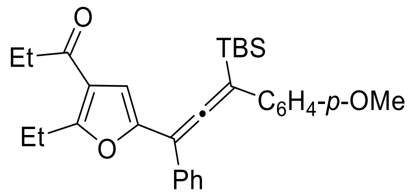

3s

1-{5-[3-(tert-butyldimethylsilyl)-3-(4-methoxyphenyl)-1-phenylpropa-1,2-dien-1-yl]-2-ethylfuran-3-yl}propan-1-one (3s)

Allene **3s** was obtained from enynone **1b** (48.1 mg, 0.20 mmol) and alkynylsilane **2n** (246.4 mg, 1.0 mmol, 5 equiv.). After 1 h, purification by flash chromatography (silica gel, hexanes/ethyl acetate 10:1) afforded compound **3s** (87.6 mg, 90% yield) as a yellow oil.

^{1}H NMR (300 MHz, $CDCl_3$): δ = 7.51 (d, *J* = 8.4 Hz, 2H), 7.42-7.31 (m, 5H), 6.91 (d, *J* = 8.4 Hz, 2H), 6.50 (s, 1H), 3.83 (s, 3H), 3.10 (q, *J* = 7.5 Hz, 2H), 2.76 (q, *J* = 7.3 Hz, 2H), 1.32 (t, *J* = 7.5 Hz, 3H), 1.19 (t, *J* = 7.3 Hz, 3H), 0.92 (s, 9H), 0.34 (s, 3H), 0.29 (s, 3H) ppm.

^{13}C NMR (75 MHz, $CDCl_3$): δ = 209.9 (C), 197.1 (C), 162.5 (C), 158.6 (C), 147.7 (C), 135.0 (C), 129.5 (C), 129.4 (CH), 128.6 (CH), 127.8 (CH), 127.3 (CH), 121.6 (C), 114.0 (CH), 107.5 (CH), 103.8 (C), 97.6 (C), 55.3 (CH_3), 34.4 (CH_2), 27.0 (CH_3), 21.8 (CH_2); 18.2 (C), 12.1 (CH_3), 7.9 (CH_3), - 4.1 (CH_3) - 4.3 (CH_3) ppm.

HRMS (EI) m/z: $[M + H]^+$ Calcd for $C_{31}H_{39}O_3Si$ 487.2663; Found 487.2663.

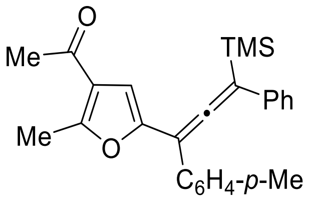

3t

1-{2-methyl-5-[3-phenyl-1-(*p*-tolyl)-3-(trimethylsilyl)propa-1,2-dien-1-yl]furan-3-yl}ethan-1-one (3t)

Allene **3t** was obtained from enynone **1c** (45.3 mg, 0.20 mmol) and alkynylsilane **2a** (174.3 mg, 1.0 mmol, 5 equiv.). After 1 h, purification by flash chromatography (silica gel, hexanes/ethyl acetate 10:1) afforded compound **3t** (58.0 mg, 70% yield) as a yellow oil.

^{1}H NMR (300 MHz, $CDCl_3$): δ = 7.53-7.18 (m, 9H), 6.50 (s, 1H), 2.64 (s, 3H), 2.42 (s, 3H), 2.41 (s, 3H), 0.35 (s, 9H) ppm.

^{13}C NMR (75 MHz, $CDCl_3$): δ = 209.2 (C), 194.3 (C), 158.0 (C), 147.9 (C), 137.4 (C), 136.3 (C), 131.8 (C), 129.5 (CH), 128.8 (CH), 128.2 (CH), 127.7 (CH), 127.0 (CH), 123.2 (C), 108.0 (CH), 105.6 (C), 98.3 (C), 29.3 (CH_3), 21.3 (CH_3), 14.7 (CH_3), - 0.1 (CH_3) ppm.

HRMS (EI) m/z: $[M + H]^+$ Calcd for $C_{26}H_{29}O_2Si$ 401.1931; Found 401.1943.

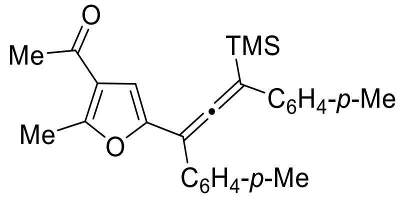

3u

1-{5-[1,3-di-p-tolyl-3-(trimethylsilyl)propa-1,2-dien-1-yl]-2-methylfuran-3-yl}ethan-1-one (3u)

Allene **3u** was obtained from enynone **1c** (45.3 mg, 0.20 mmol) and alkynylsilane **2b** (188.3 mg, 1.0 mmol, 5 equiv.). After 40 min, purification by flash chromatography (silica gel, hexanes/ethyl acetate 10:1) afforded compound **3u** (56.4 mg, 68% yield) as a yellow oil.

^{1}H NMR (300 MHz, $CDCl_3$): δ = 7.38 (d, *J* = 8.2 Hz, 2H), 7.33 (d, *J* = 8.2 Hz, 2H), 7.21-7.15 (m, 4H), 6.48 (s, 1H), 2.62 (s, 3H), 2.40 (s, 3H), 2.39 (s, 3H), 2.36 (s, 3H), 0.33 (s, 9H) ppm.

^{13}C NMR (75 MHz, $CDCl_3$): δ = 209.0 (C), 194.2 (C), 157.9 (C), 148.0 (C), 137.3 (C), 136.8 (C), 133.2 (C), 131.9 (C), 129.51 (CH), 129.46 (CH), 129.1 (C), 128.0 (CH), 127.6 (CH), 123.2 (C), 107.9 (CH), 105.3 (C), 98.3 (C), 29.3 (CH_3), 21.3 (CH_3), 21.2 (CH_3), 14.7 (CH_3), - 0.1 (CH_3) ppm.

HRMS (EI) m/z: $[M + Na]^+$ Calcd for $C_{27}H_{30}NaO_2Si$ 437.1907; Found 437.1906.

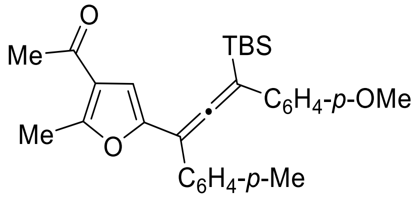

3v

1-{5-[3-(*tert*-butyldimethylsilyl)-3-(4-methoxyphenyl)-1-(*p*-tolyl)propa-1,2-dien-1-yl]-2-methylfuran-3-yl}ethan-1-one (3v)

Allene **3v** was obtained from enynone **1c** (45.3 mg, 0.20 mmol) and alkynylsilane **2n** (246.4 mg, 1.0 mmol, 5 equiv.). After 50 min, purification by flash chromatography (silica gel, hexanes/ethyl acetate 10:1) afforded compound **3v** (89.8 mg, 95% yield) as a yellow oil.

^{1}H NMR (300 MHz, $CDCl_3$): δ = 7.43 (d, *J* = 8.0 Hz, 2H), 7.39 (d, *J* = 8.9 Hz, 2H), 7.25 (d, *J* = 7.9 Hz, 2H), 6.94 (d, *J* = 8.8 Hz, 2H), 6.52 (s, 1H), 3.87 (s, 3H), 2.68 (s, 3H), 2.46 (s, 3H), 2.44 (s, 3H), 0.96 (s, 9H), 0.38 (s, 3H), 0.32 (s, 3H) ppm.

^{13}C NMR (75 MHz, $CDCl_3$): δ = 209.9 (C), 194.2 (C), 158.5 (C), 157.6 (C), 148.0 (C), 137.1 (C), 131.9 (C), 129.5 (CH), 129.3 (CH), 127.7 (CH), 123.1 (C), 114.0 (CH), 107.8 (CH), 103.6 (C), 97.4 (C), 55.3 (CH_3), 29.2 (CH_3), 27.0 (CH_3), 21.2 (CH_3), 18.2 (C), 14.5 (CH_3), - 4.0 (CH_3), -4.2 (CH_3) ppm.

HRMS (EI) m/z: $[M + Na]^+$ Calcd for $C_{30}H_{36}NaO_3Si$ 495.2326; Found 495.2325.

5. Synthesis of (*E*)-1-[5-(hex-1-en-1-yl)-2-methylfuran-3-yl]ethan-1-one (4a)

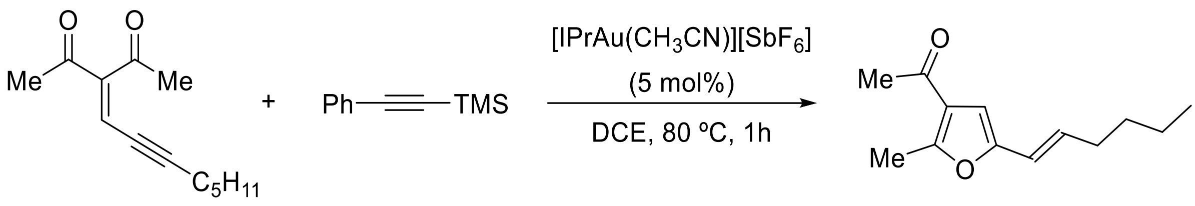

1e **2a** **4a**

$[(IPr)Au(CH_3CN)]SbF_6$ (8.6 mg, 5.0 mol%) was added to a solution of enynone **1e** (41.3 mg, 0.20 mmol) and alkynylsilane **2a** (174.3 mg, 1.0 mmol, 5 equiv.) in DCE (1 mL). The resulting mixture was stirred at 80 ºC until disappearance of the starting propargyl ester (checked by TLC, 1.0 h). Then, the solvent was removed under reduced pressure and the resulting mixture was purified by flash chromatography (silica gel, hexanes/ethyl acetate 10:1) to afforded compound **4a** (35.5 mg, 86% yield) as a yellow oil.

(*E*)-1-[5-(hex-1-en-1-yl)-2-methylfuran-3-yl]ethan-1-one (4a)

^{1}H NMR (300 MHz, $CDCl_3$): δ = 6.28 (s, 1H), 6.16 (dt, *J* = 15.8 and 6.2 Hz, 1H), 6.07 (d, *J* = 15.9 Hz, 1H), 2.55 (s,3H), 2.35 (s, 3H), 2.16 (q, *J* = 6.6 Hz, 2H), 1.47-1.31 (m, 4H), 0.90 (t, *J* = 7.2 Hz, 3H) ppm.

The spectral data of compound **4a** matched the literature.[4]

6. Experimental Procedure for the Synthesis of 3a (2.5 mmol Scale)

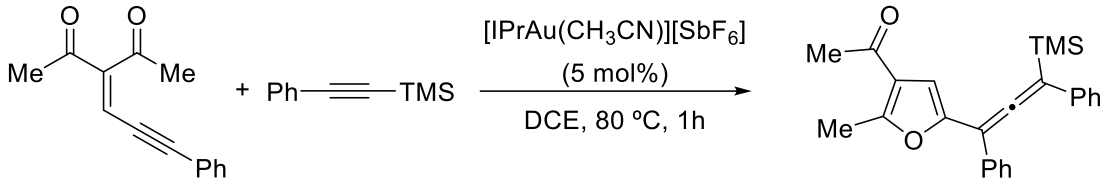

1a (2.5 mmol) **2a** **3a** (696 mg, 72%)

[(IPr)Au(CH_3CN)]SbF_6 (107 mg, 5.0 mol%) was added to a solution of enynone **1a** (530.6 mg, 2.5 mmol) and alkynylsilane **2a** (2.2 g, 12.5 mmol, 5 equiv) in DCE (10 mL). The resulting mixture was stirred at 80 ºC until disappearance of the starting propargyl ester (checked by TLC, 1.5 h). Then, the solvent was removed under reduced pressure and the resulting mixture was purified by flash chromatography (silica gel, hexanes/ethyl acetate 10:1) to yield compound **3a** (696 mg, 72%). The spectroscopic data of compound **3a** match with those reported for the 0.20 mmol scale (see page S-6).

7. Synthesis of Indene Derivative 5

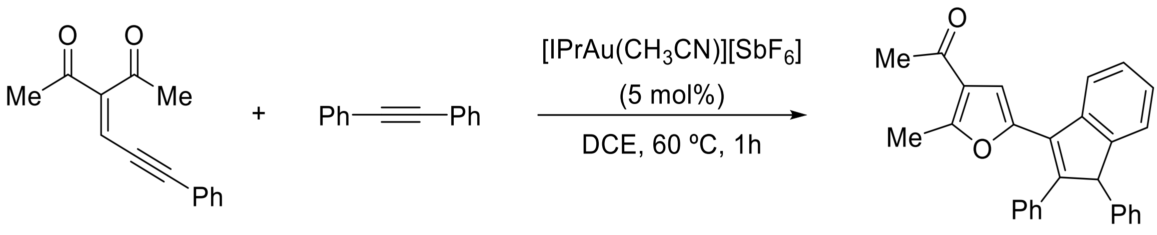

1a **2s** **5**, 18%

[(IPr)Au(CH_3CN)]SbF_6 (8.6 mg, 5.0 mol%) was added to a solution of enynone **1a** (42.5 mg, 0.20 mmol) and 1,2-diphenylacetylene **2s** (178.2 mg, 1.0 mmol, 5 equiv.) in DCE (1 mL). The resulting mixture was stirred at 60 ºC until disappearance of the starting propargyl ester (checked by TLC, 1.0 h). Then, the solvent was removed under reduced pressure and the resulting mixture was purified by flash chromatography (silica gel, hexanes/ethyl acetate 10:1) to afforded compound **5** (14.1 mg, 18% yield) as an orange oil.

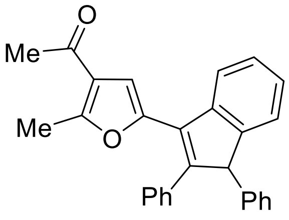

5

1-[5-(1,2-diphenyl-1*H*-inden-3-yl)-2-methylfuran-3-yl]ethan-1-one (5)

^{1}H NMR (300 MHz, $CDCl_3$): δ = 7.79 (t, *J* = 7.6 Hz, 2H), 7.55-7.16 (m, 10H), 7.09-6.98 (m, 2H), 6.53 (s, 1H), 5.02 (s, 1H), 2.61 (s, 3H), 2.35 (s, 3H) ppm.

^{13}C NMR (75 MHz, $CDCl_3$): δ = 194.3 (C), 157.8 (C), 148.4 (C), 148.0 (C), 147.6 (C), 142.5 (C), 139.1 (C), 136.2 (C), 130.0 (C), 129.1 (CH), 128.8 (CH), 128.5 (CH), 128.2 (CH), 127.7 (CH), 127.3 (CH), 127.0 (CH), 126.2 (CH), 124.3 (CH), 122.8 (C), 121.4 (CH) 110.0 (CH), 59.6 (CH), 29.2 (CH_3), 14.6 (CH_3) ppm.

HRMS (EI) m/z: $[M + Na]^+$ Calcd for $C_{28}H_{22}NaO_2$ 413.1512; Found 413.1509.

8. References

(1) a) Wang, T.; Zhang, J. *Dalton Trans.* **2010**, *39*, 4270; b) Vicente, R.; González, J.; Riesgo, L.; González, J.; López, L. A. *Angew. Chem. Int. Ed.* **2012**, *51*, 8063; c) Barluenga, J.; Riesgo, L.; Vicente, R.; López, L. A.; Tomás, M. *J. Am. Chem. Soc.* **2007**, *129*, 7772.

(2) a) Chuprun, S.; Acosta, C. M.; Mathivathanan, L.; Bukhryakov, K. V. *Organometallics* **2020**, *39*, 3453; b) Rajkiewicz, A. A.; Wojciechowska, N.; Kalek, M. *ACS Catal.* **2020**, *10*, 831.

(3) de Frémont, P.; Marion, N.; Nolan, S. P. *J. Organomet. Chem.* **2009**, *694*, 551.

(4) a) Cao, H.; Zhan, H.; Cen, J.; Lin, J; Lin, Y.; Zhu, Q.; Fu, M.; Jiang, H. *Org. Lett.* **2013**, *15*, 1080; b) Zhan, H; Lin, X.; Qiu, Y.; Du, Z; Li, P.; Li, Y.; Cao, H. *Eur. J. Org. Chem.* **2013**, 2284.

^{1}H NMR of compound 3a (300 MHz, $CDCl_3$)

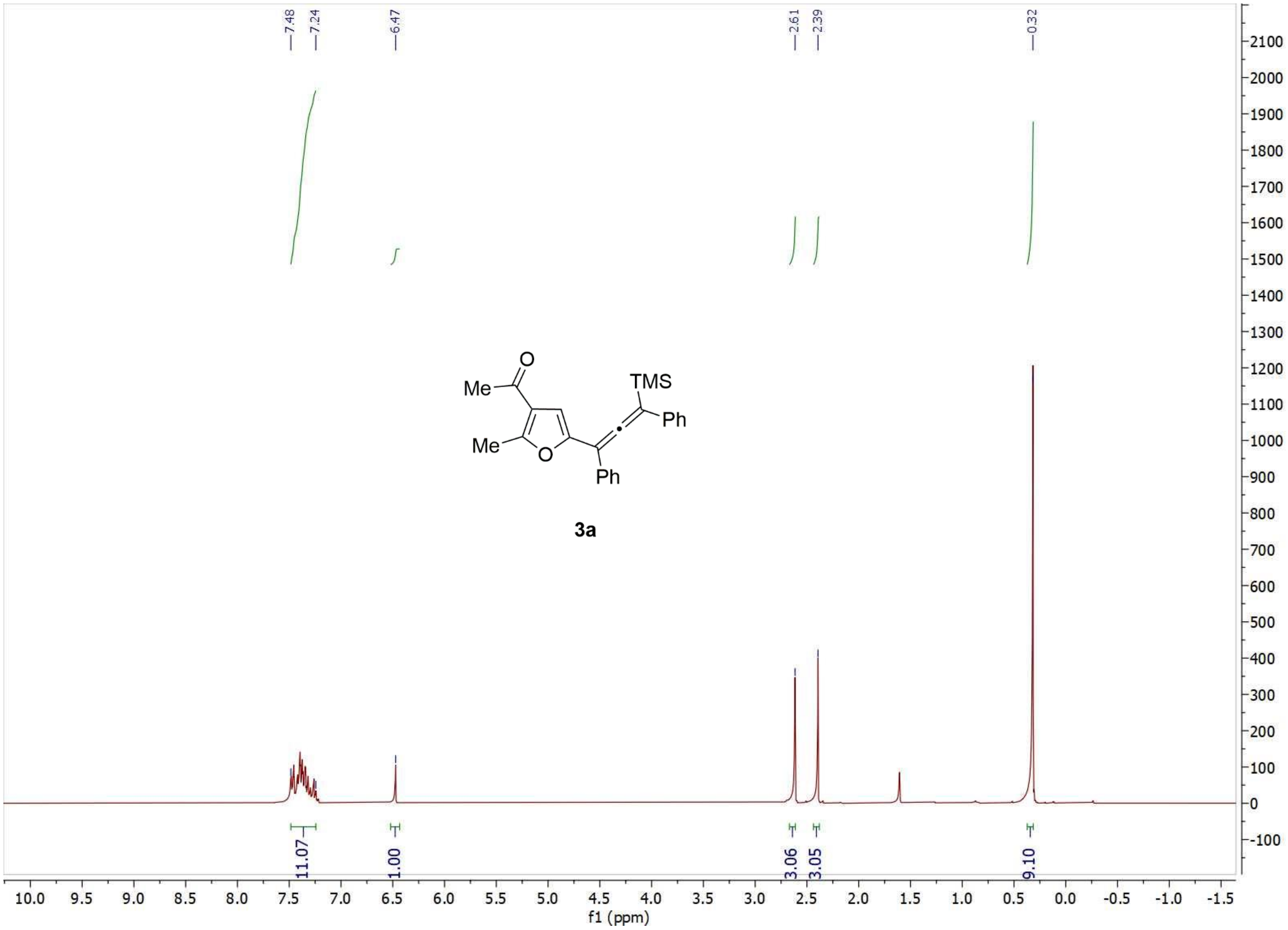

^{13}C NMR of compound 3a (75 MHz, $CDCl_3$)

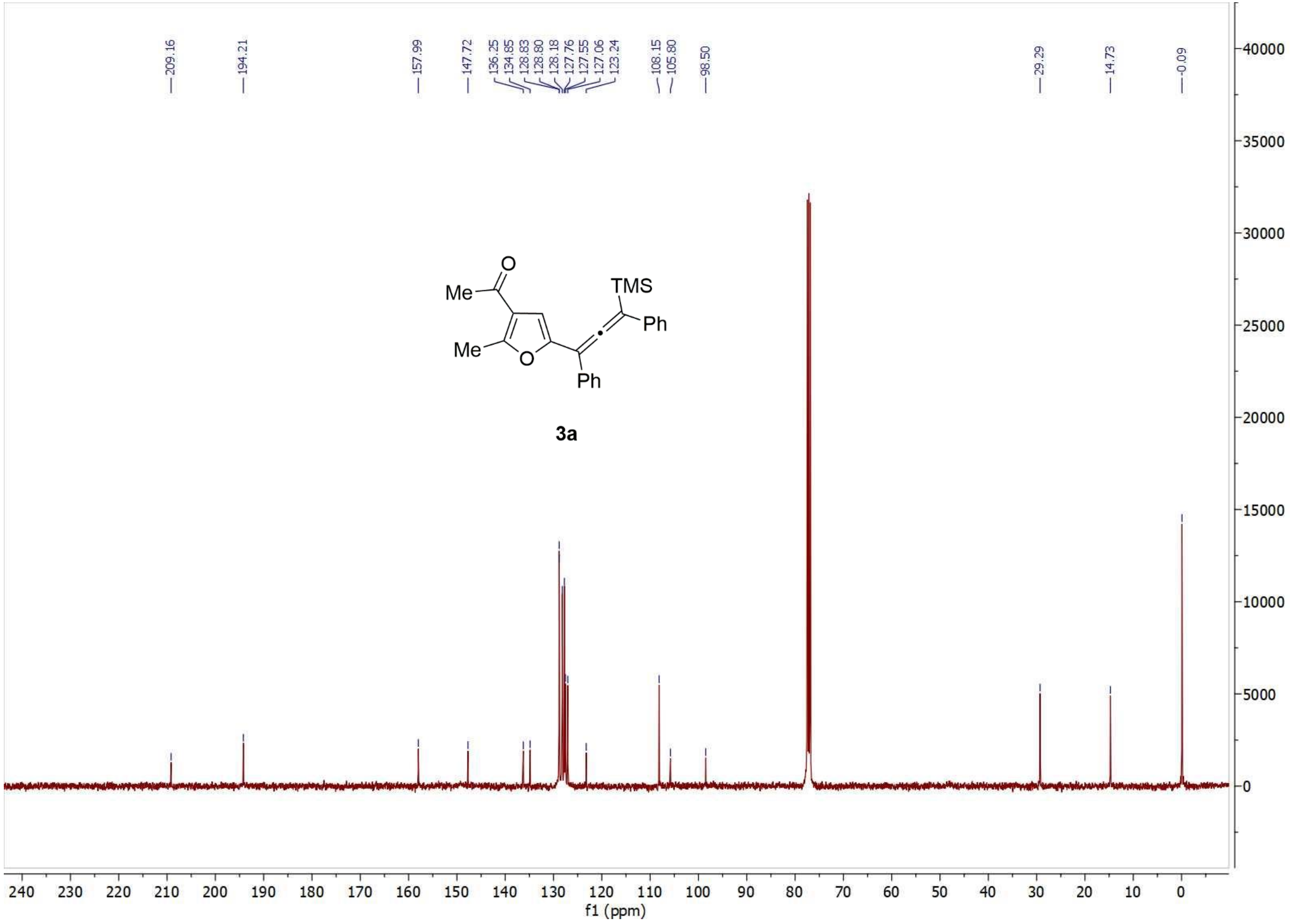

DEPT ^{13}C NMR of compound 3a (75 MHz, $CDCl_3$)

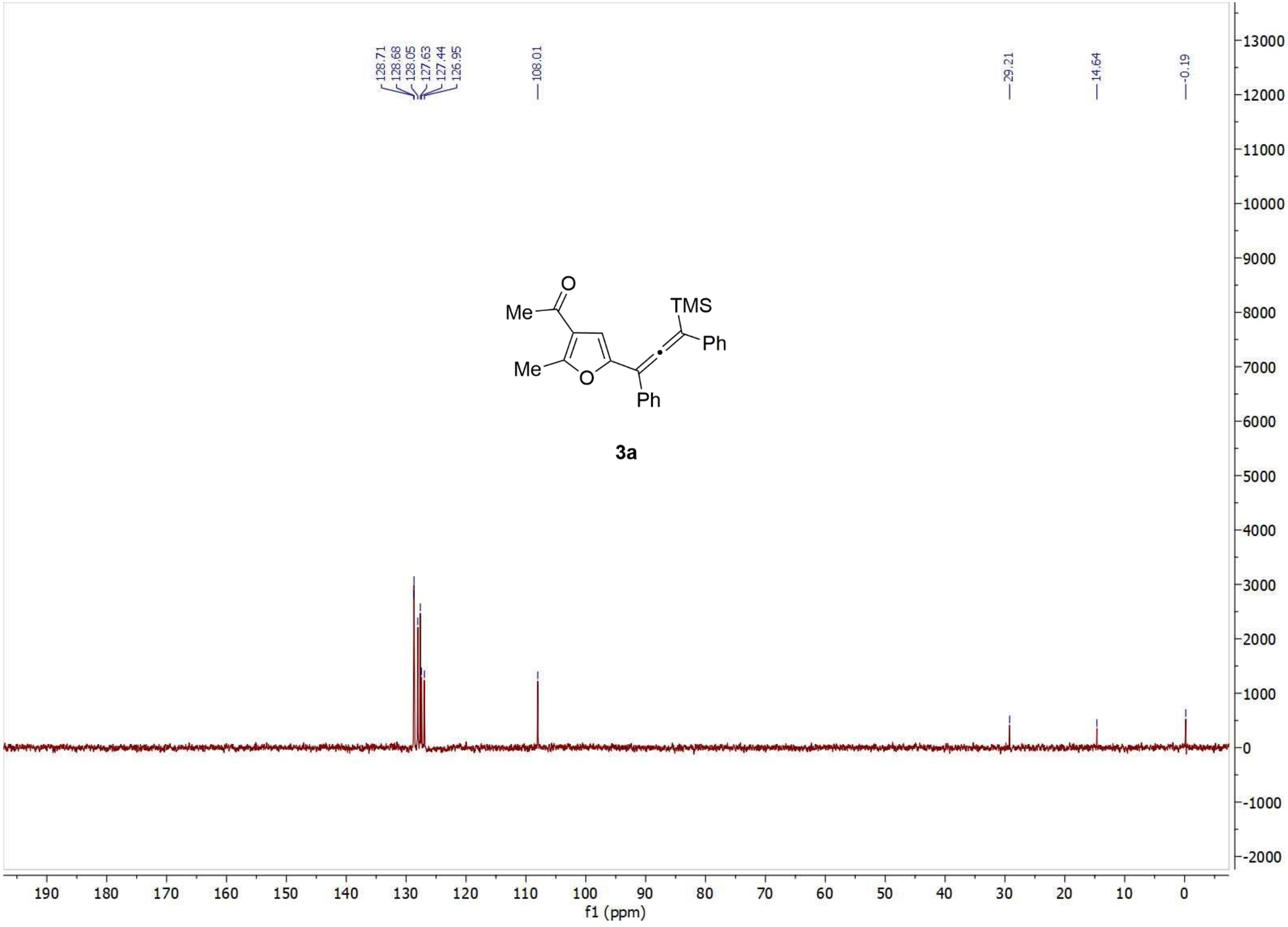

^{1}H NMR of compound 3b (300 MHz, $CDCl_3$)

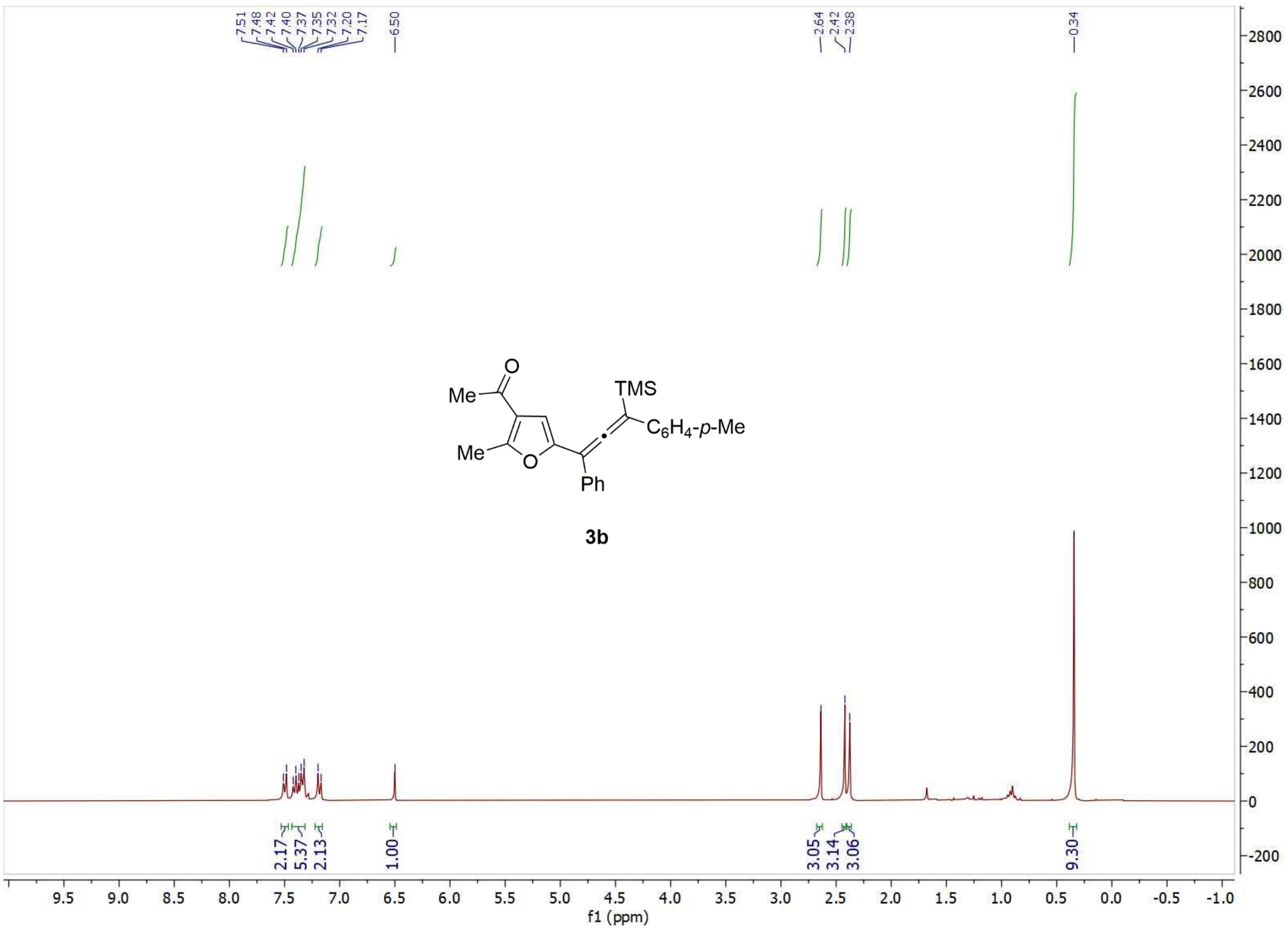

^{13}C NMR of compound 3b (75 MHz, $CDCl_3$)

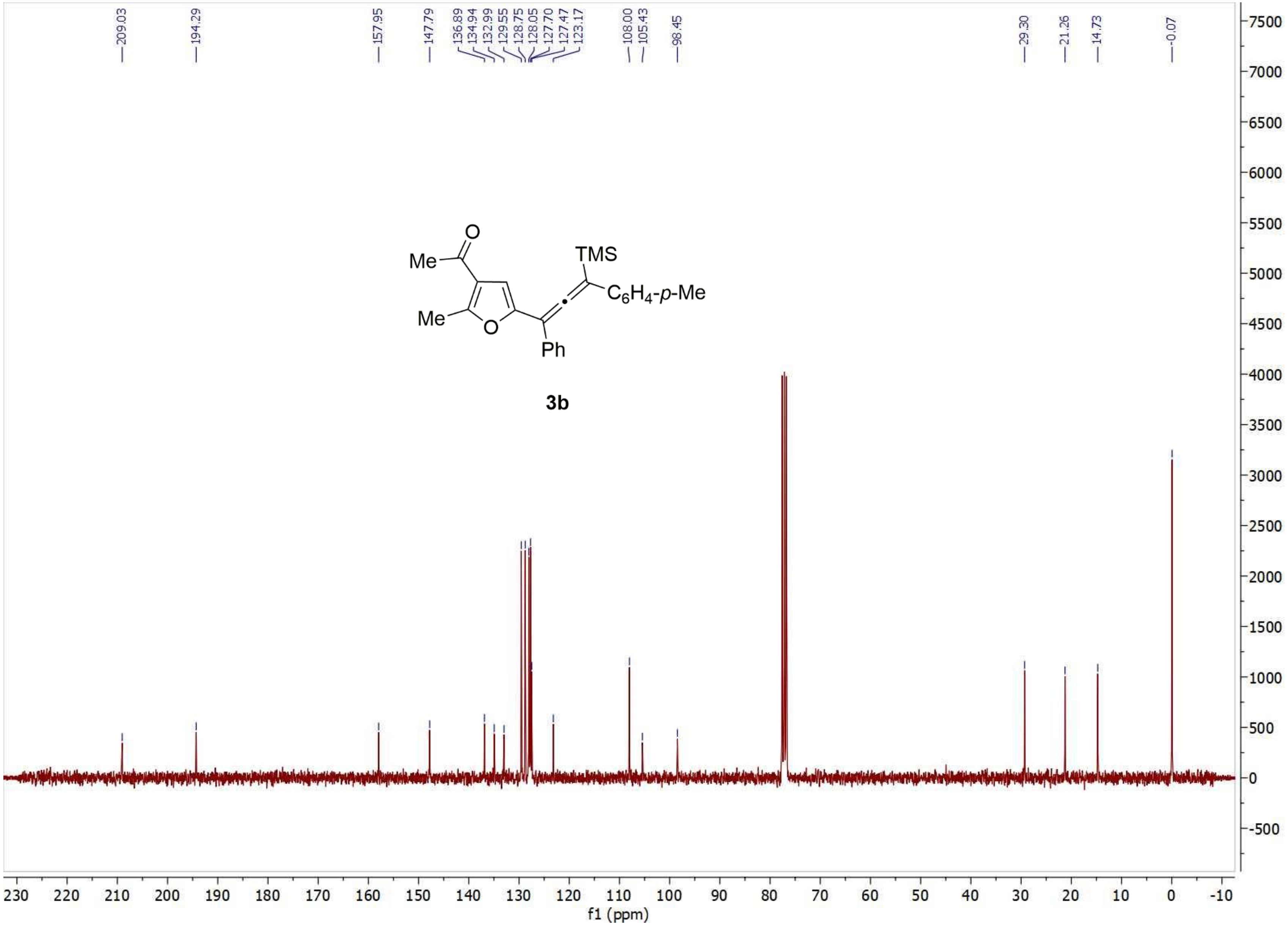

DEPT ^{13}C NMR of compound 3b (75 MHz, $CDCl_3$)

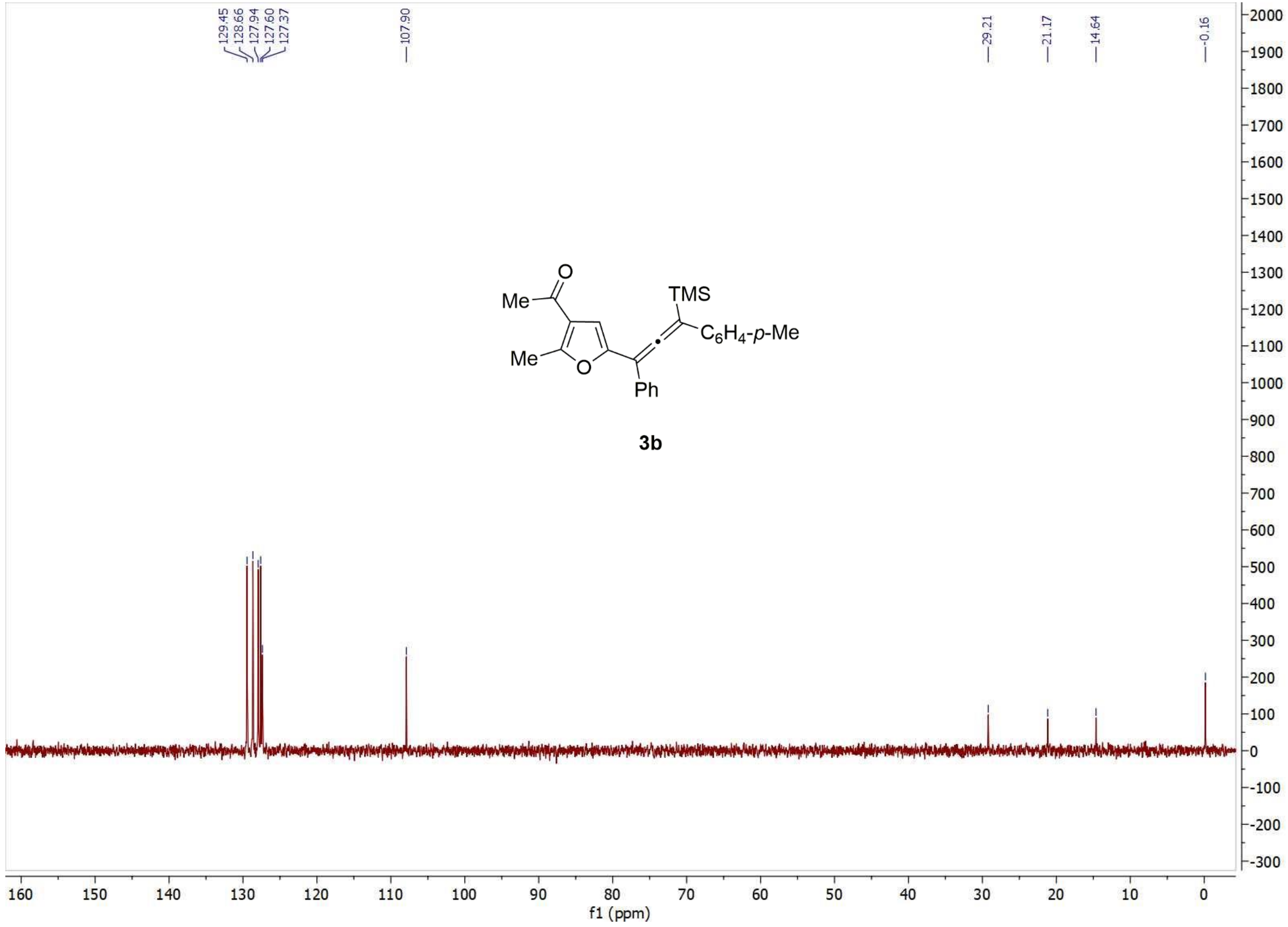

^{1}H NMR of compound 3c (300 MHz, $CDCl_3$)

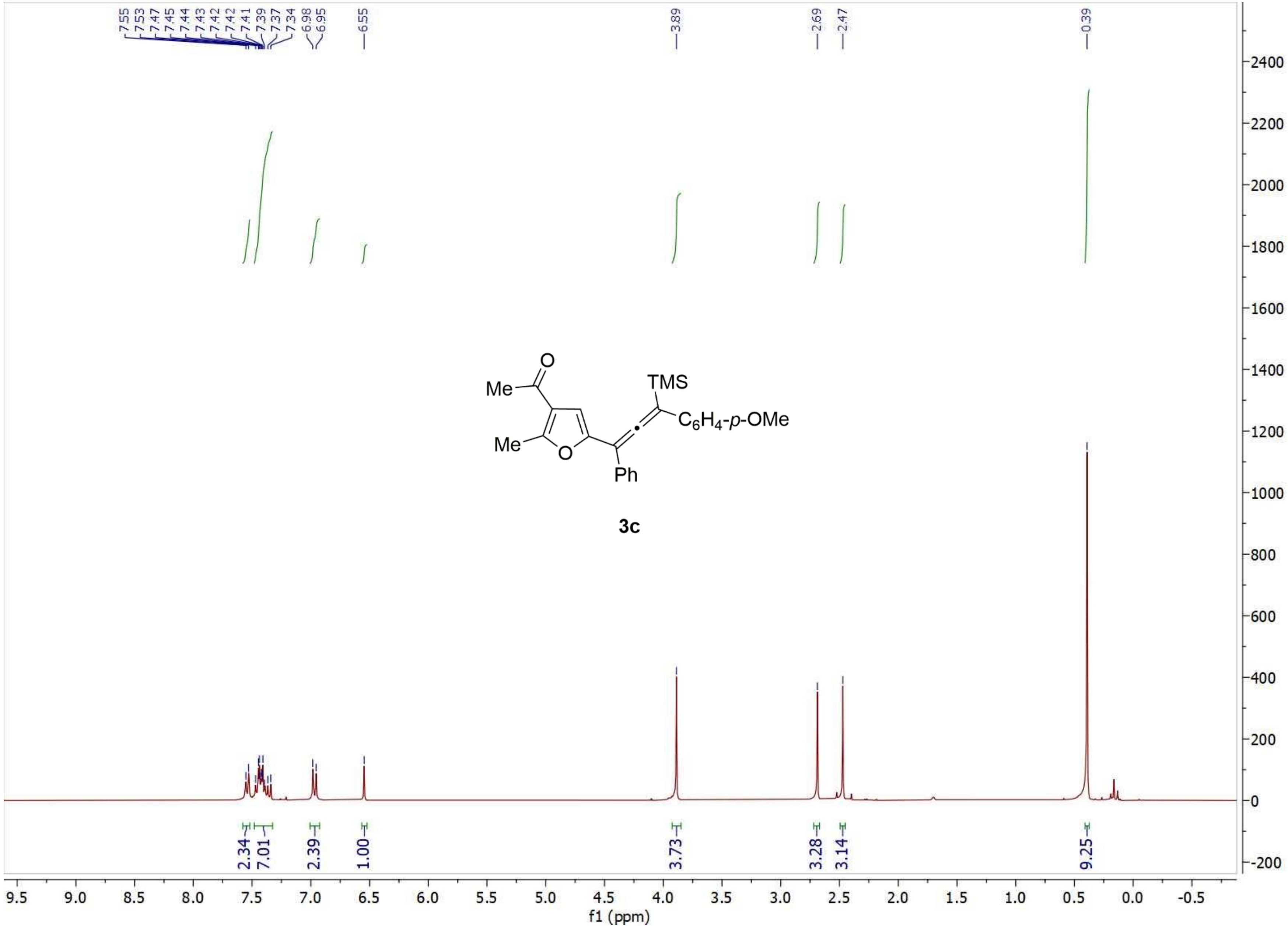

^{13}C NMR of compound 3c (75 MHz, $CDCl_3$)

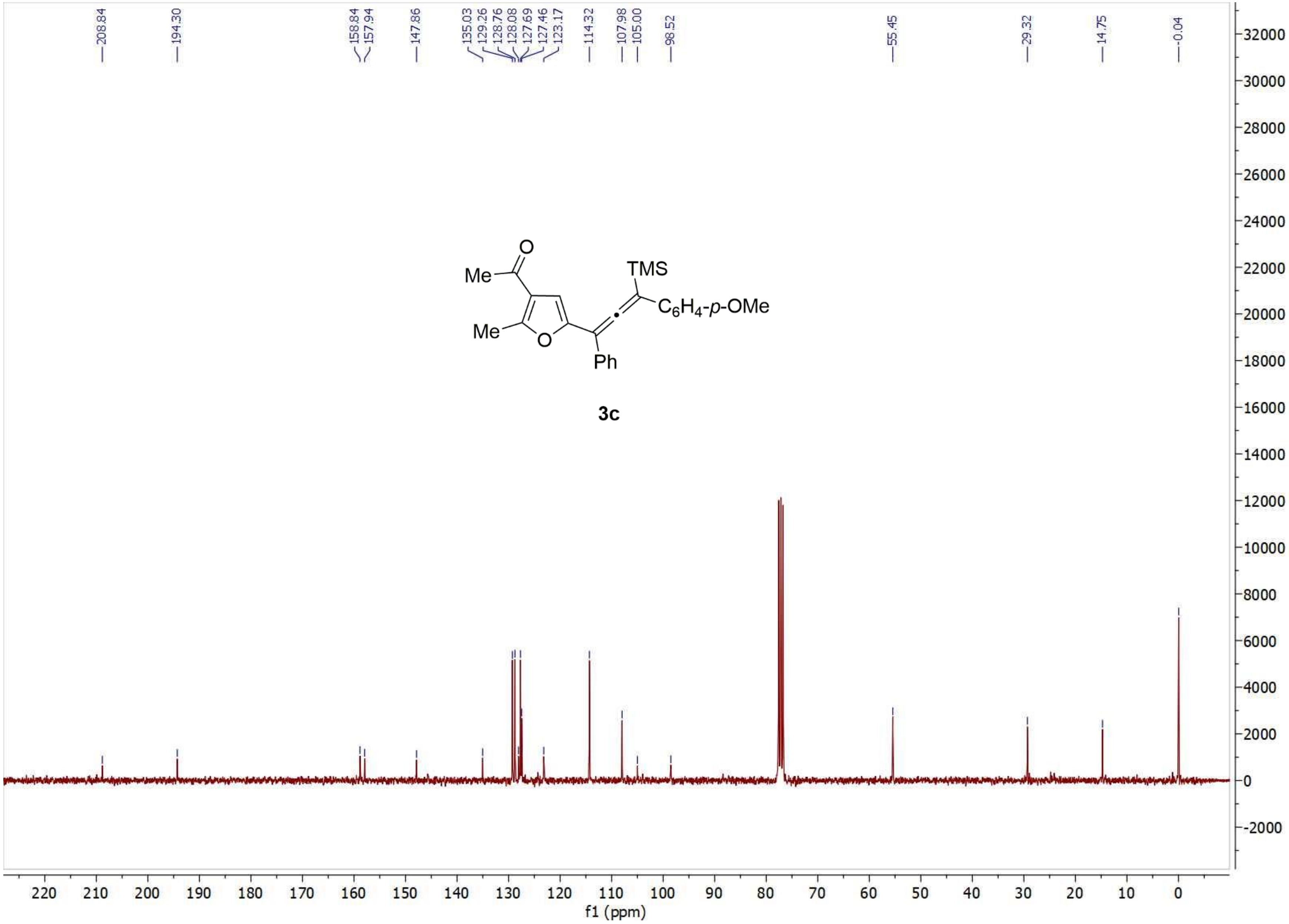

DEPT ^{13}C NMR of compound 3c (75 MHz, $CDCl_3$)

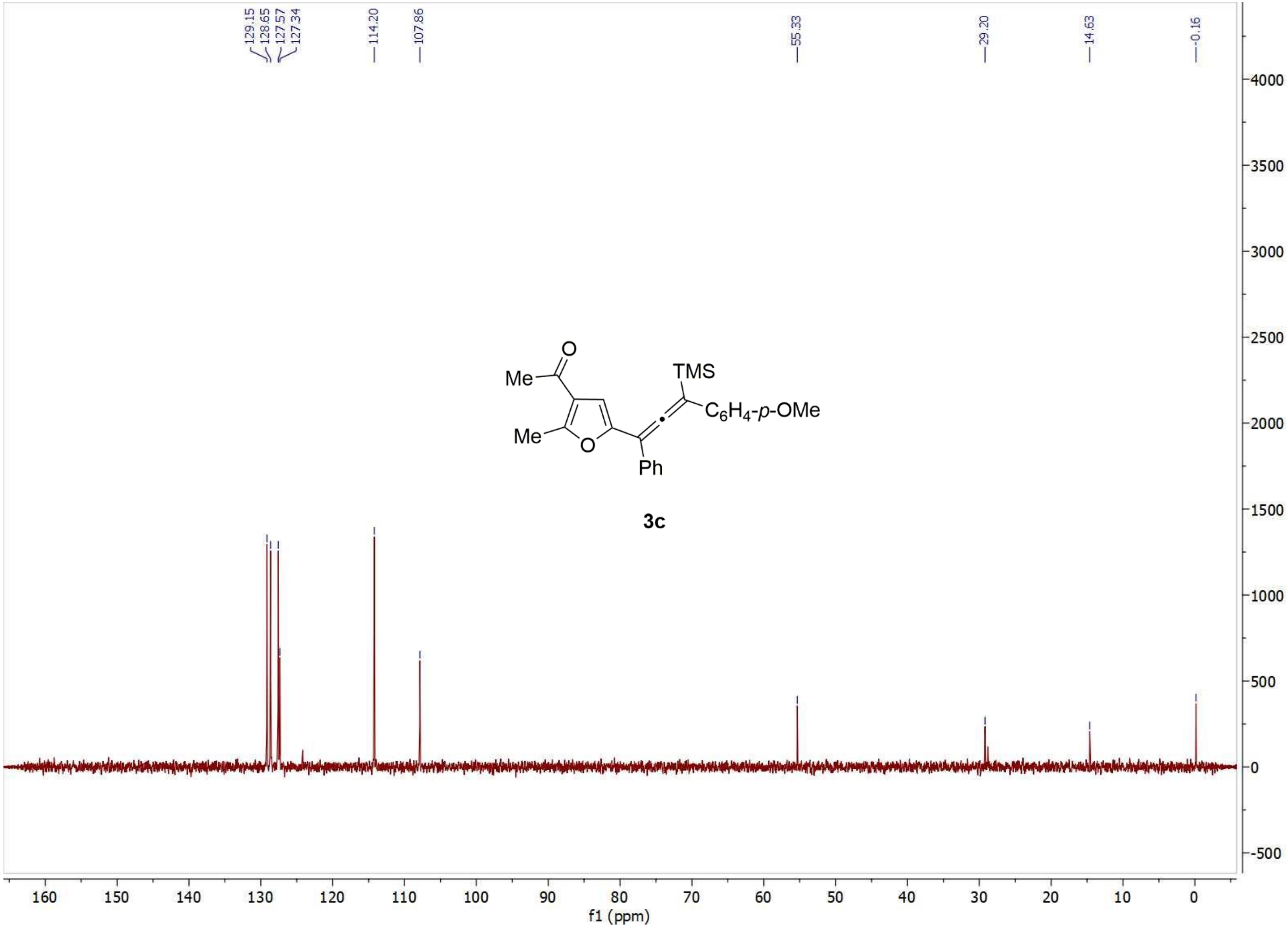

^{1}H NMR of compound 3d (300 MHz, $CDCl_3$)

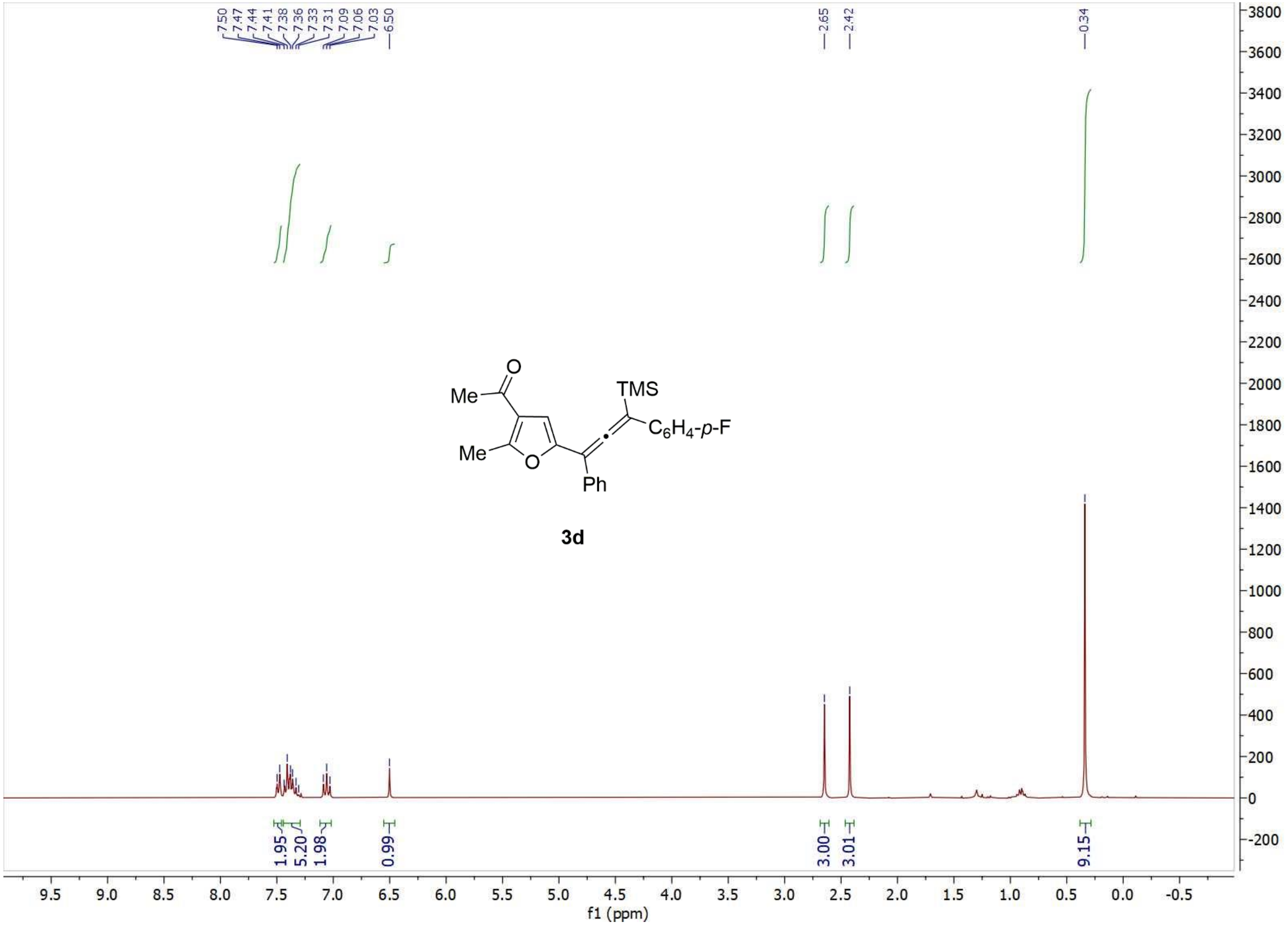

^{13}C NMR of compound 3d (75 MHz, $CDCl_3$)

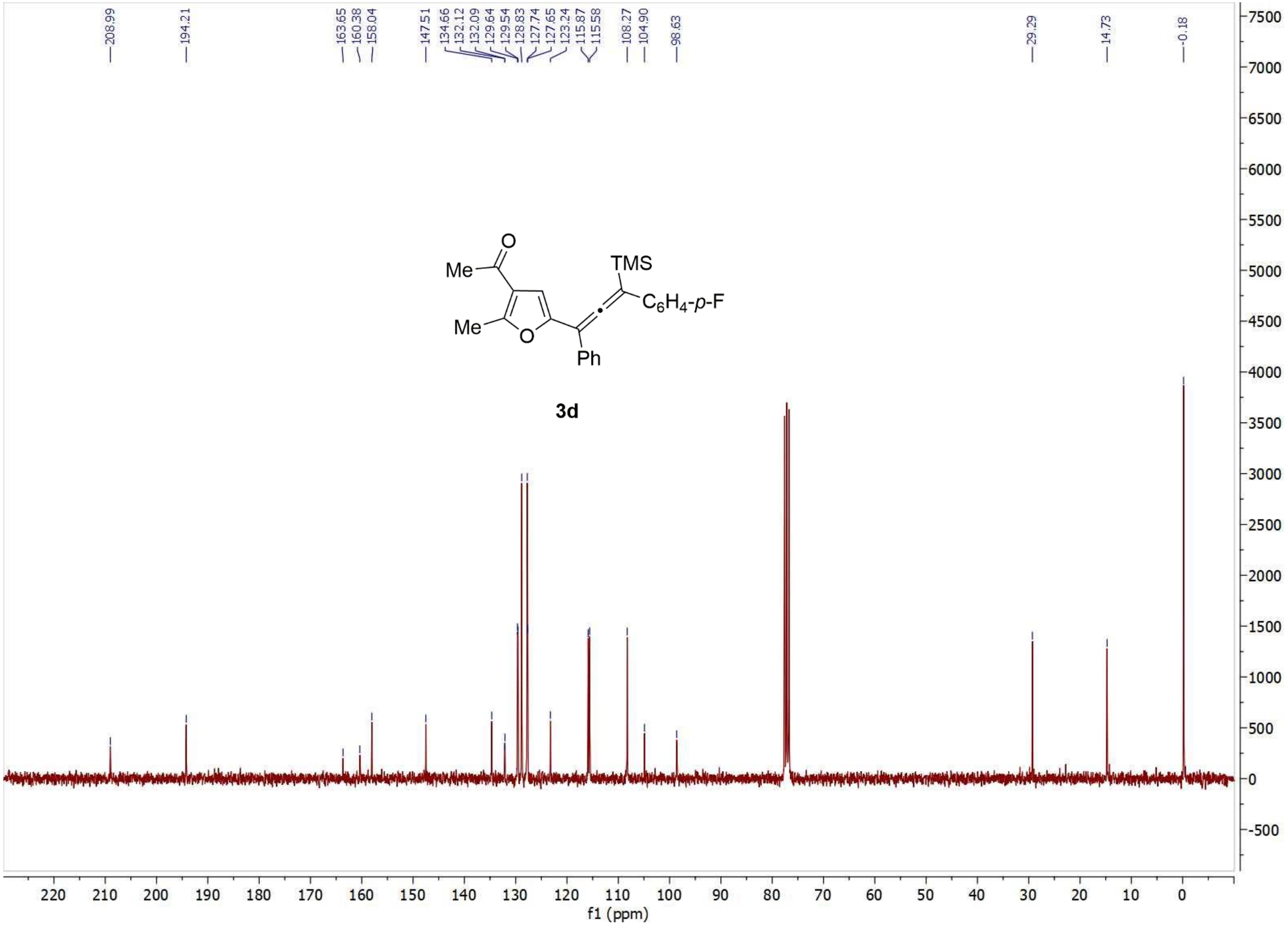

DEPT ^{13}C NMR of compound 3d (75 MHz, $CDCl_3$)

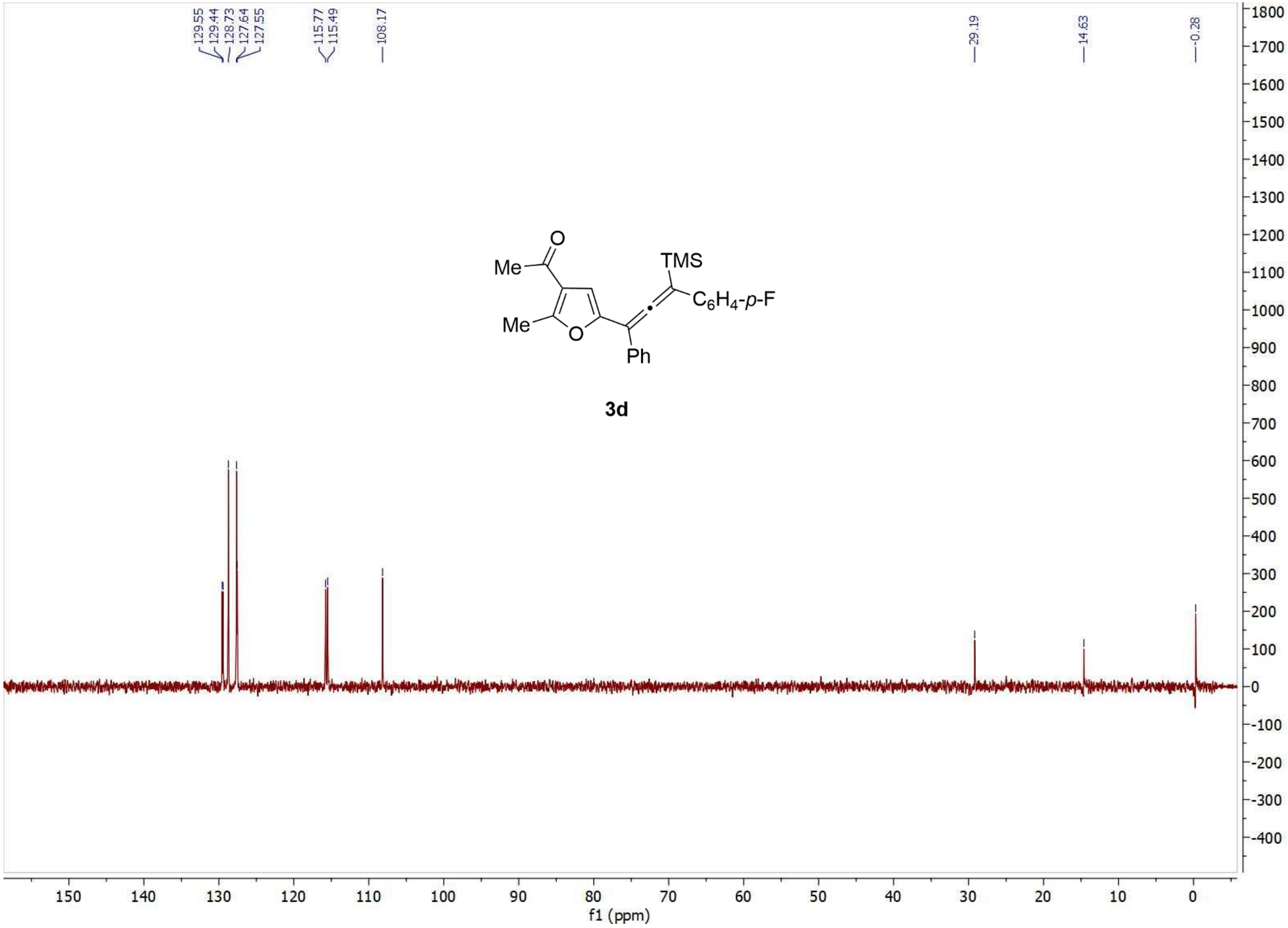

^{19}F NMR of compound 3d (282 MHz, $CDCl_3$)

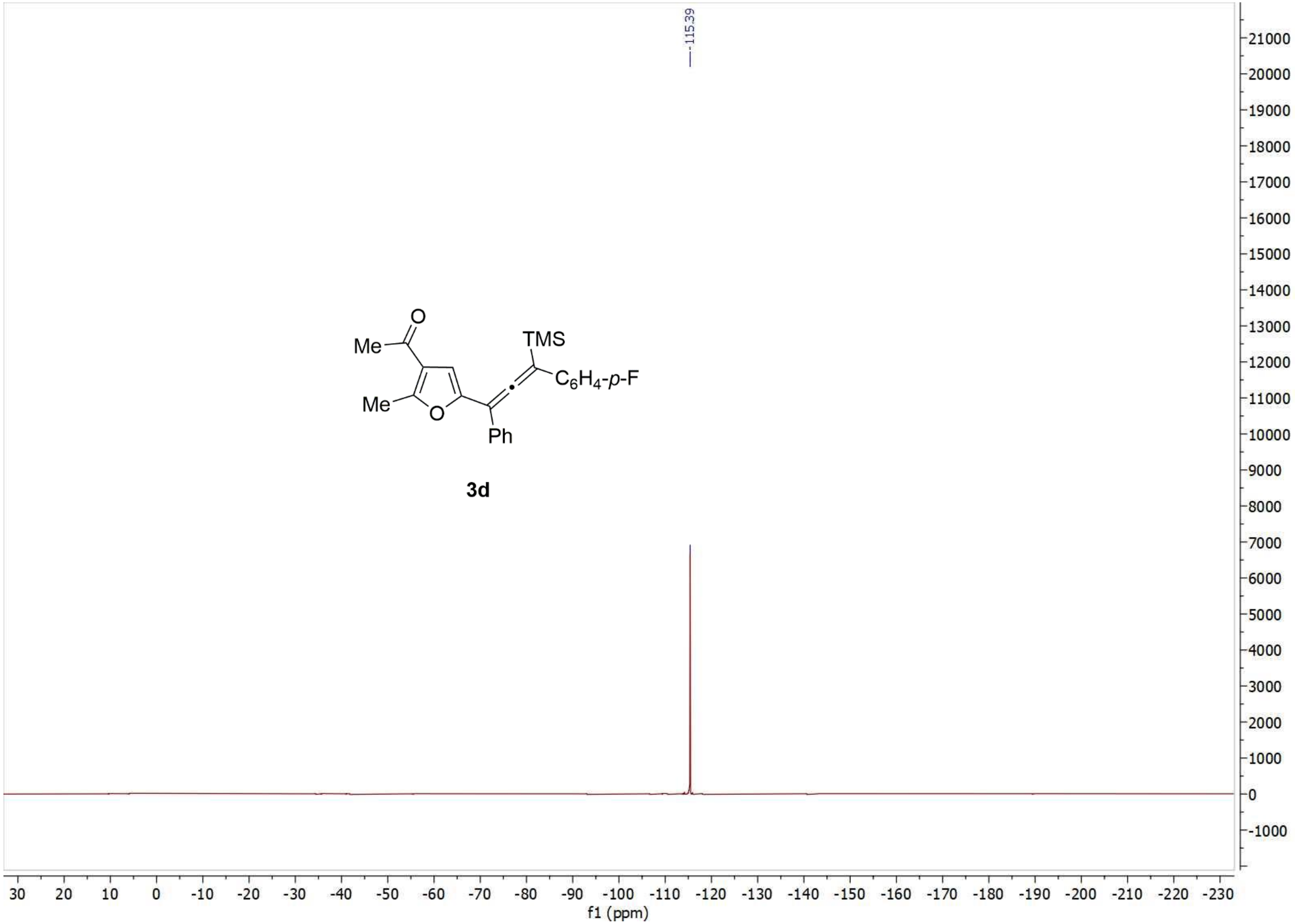

^{1}H NMR of compound 3e (300 MHz, $CDCl_3$)

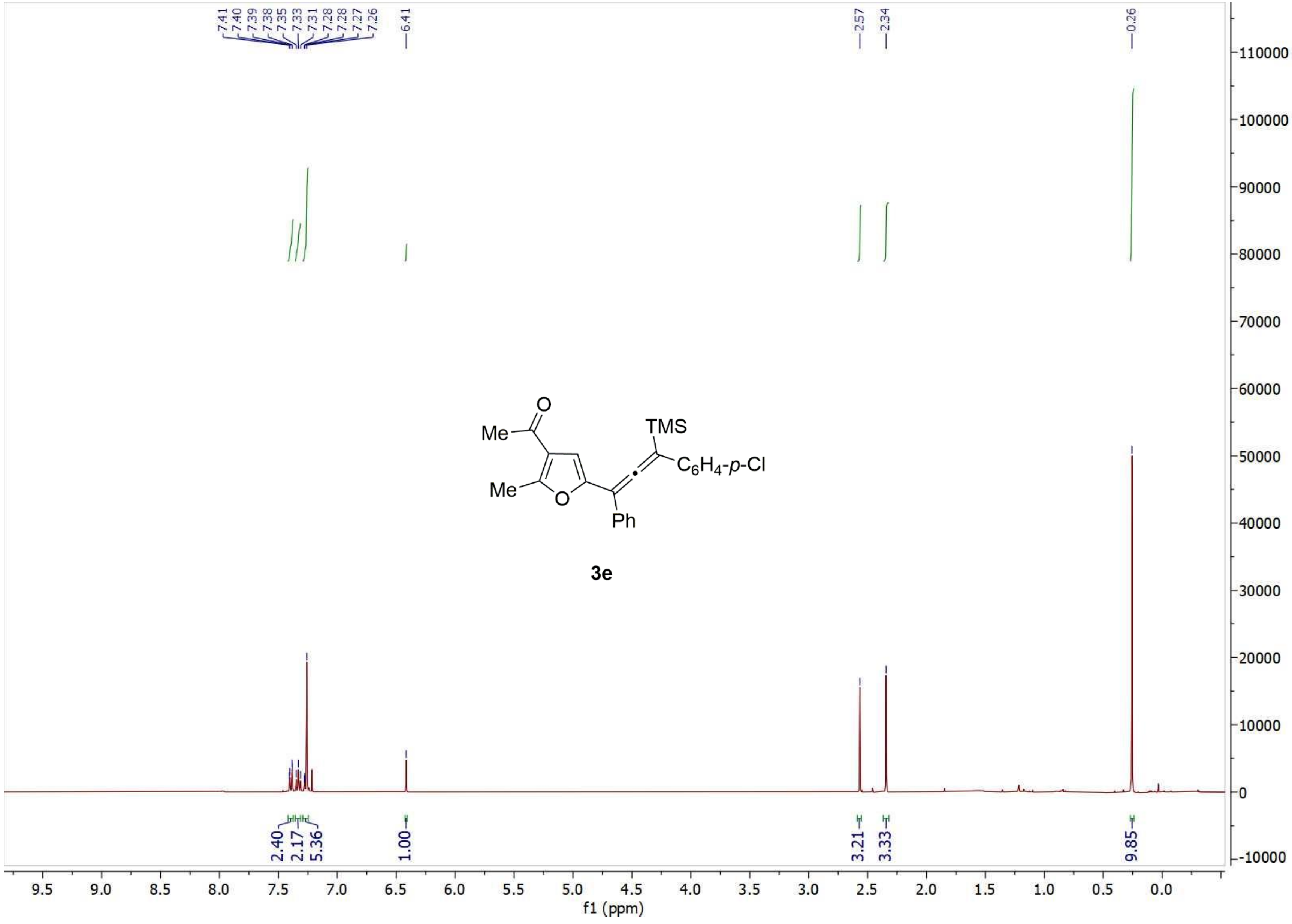

^{13}C NMR of compound 3e (75 MHz, $CDCl_3$)

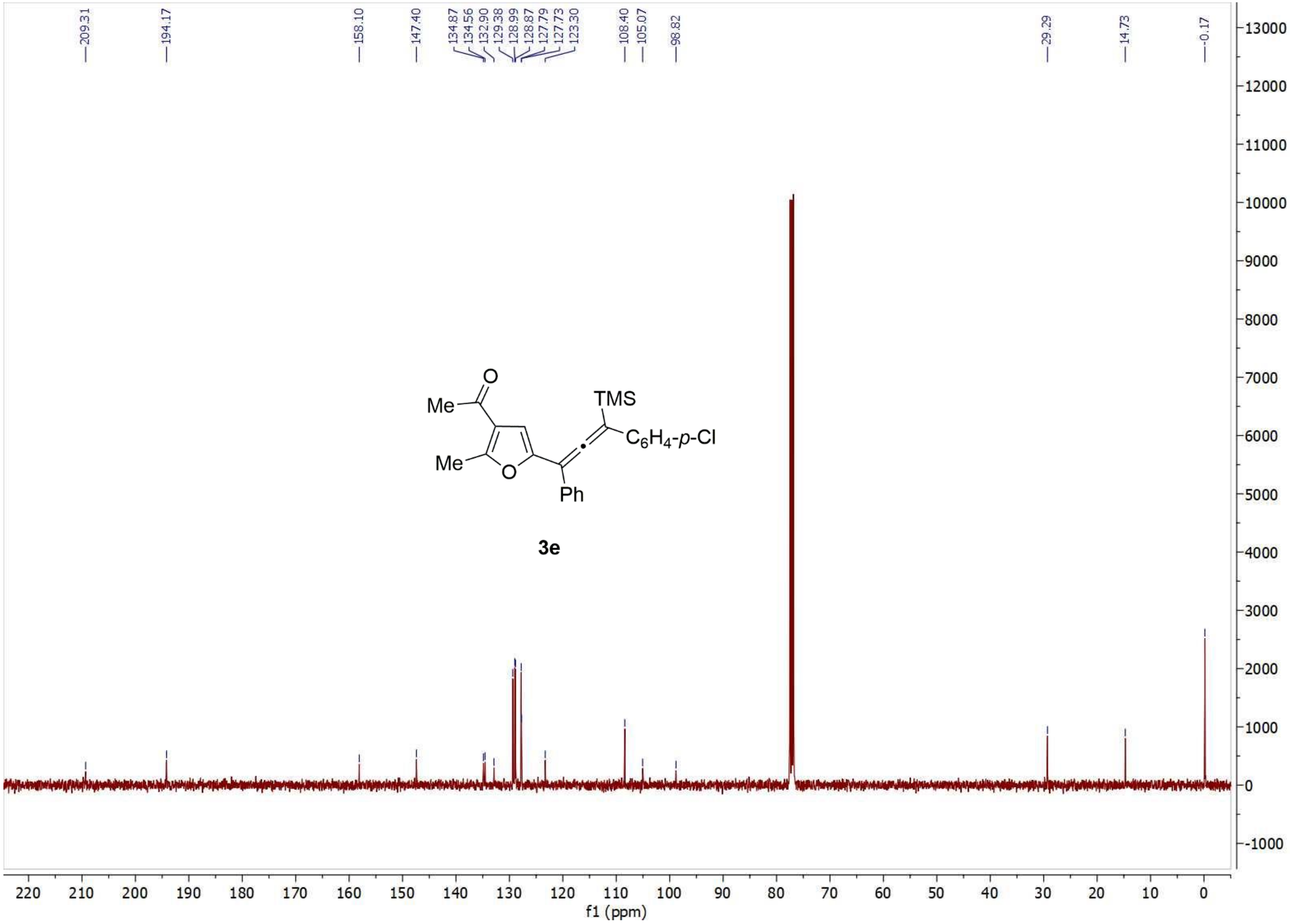

DEPT ^{13}C NMR of compound 3e (75 MHz, $CDCl_3$)

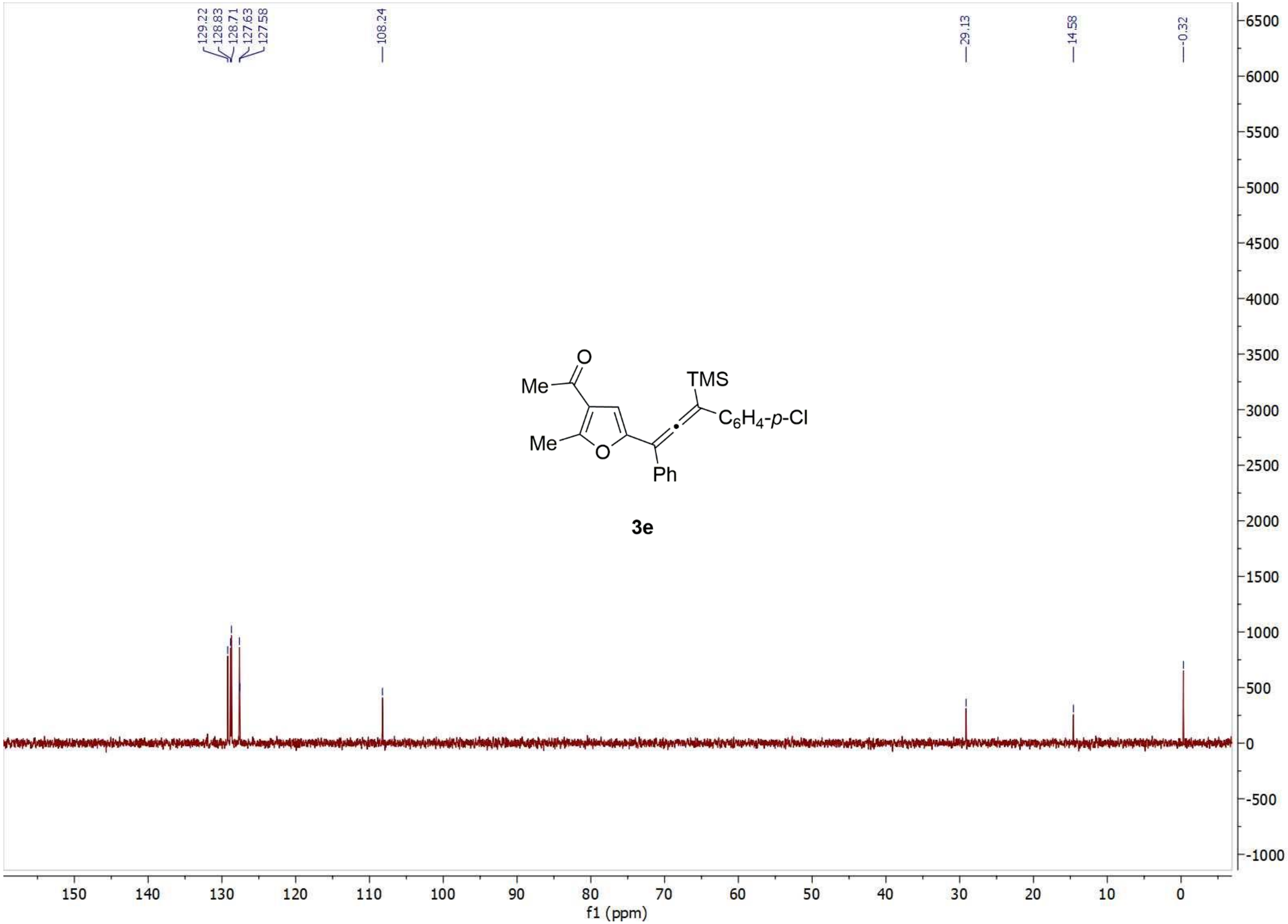

^{1}H NMR of compound 3f (300 MHz, $CDCl_3$)

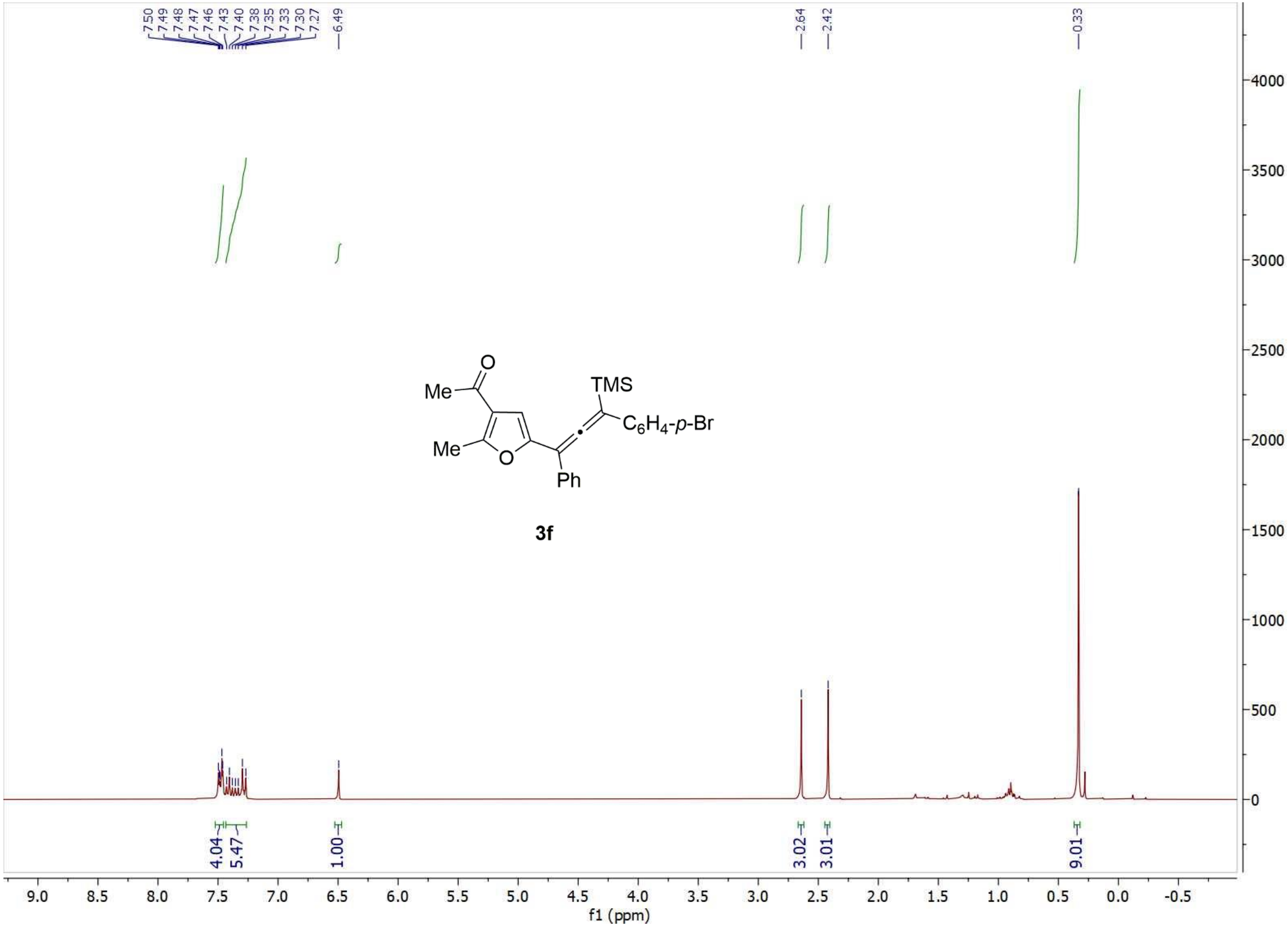

^{13}C NMR of compound 3f (75 MHz, $CDCl_3$)

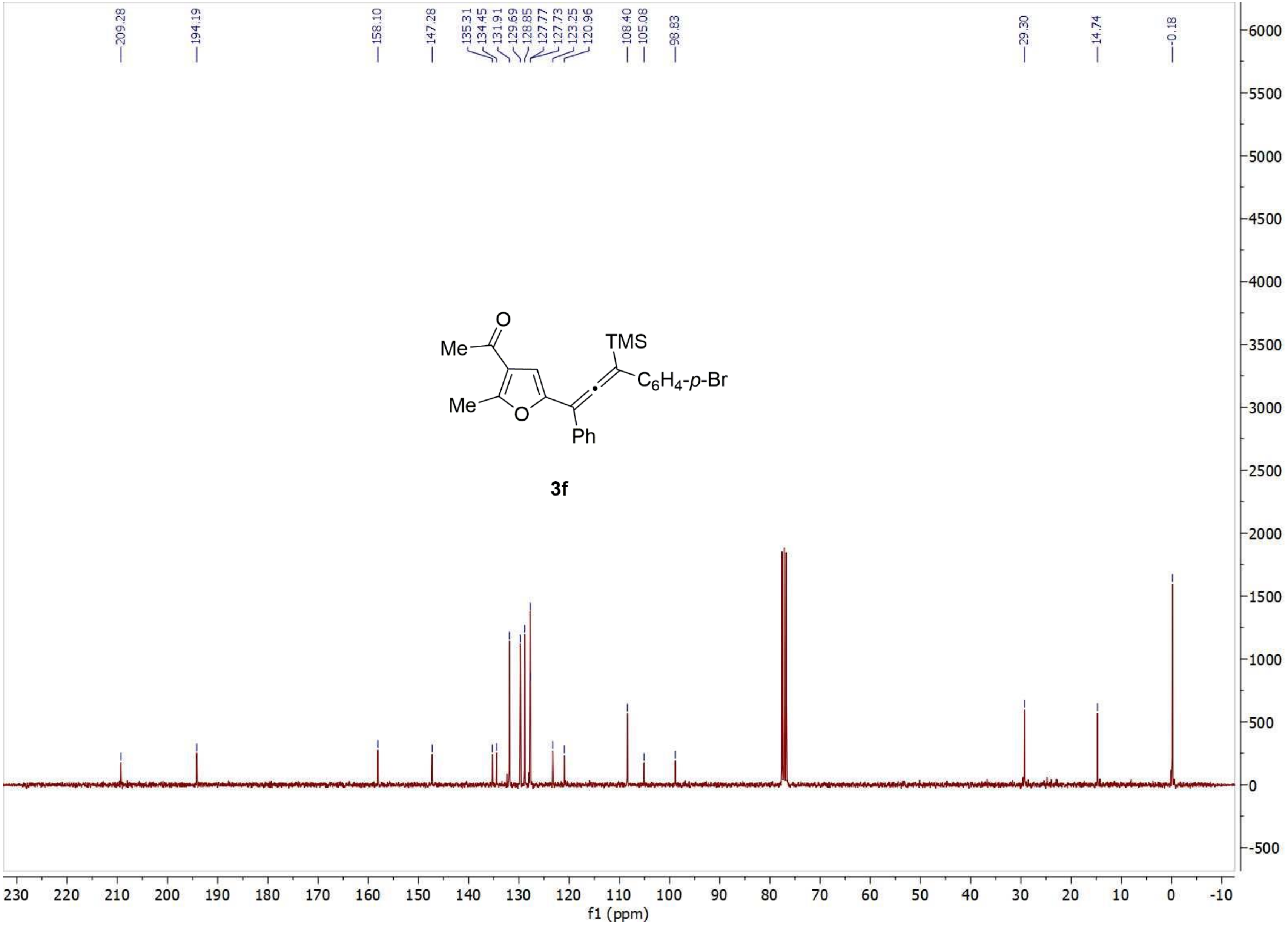

DEPT ^{13}C NMR of compound 3f (75 MHz, $CDCl_3$)

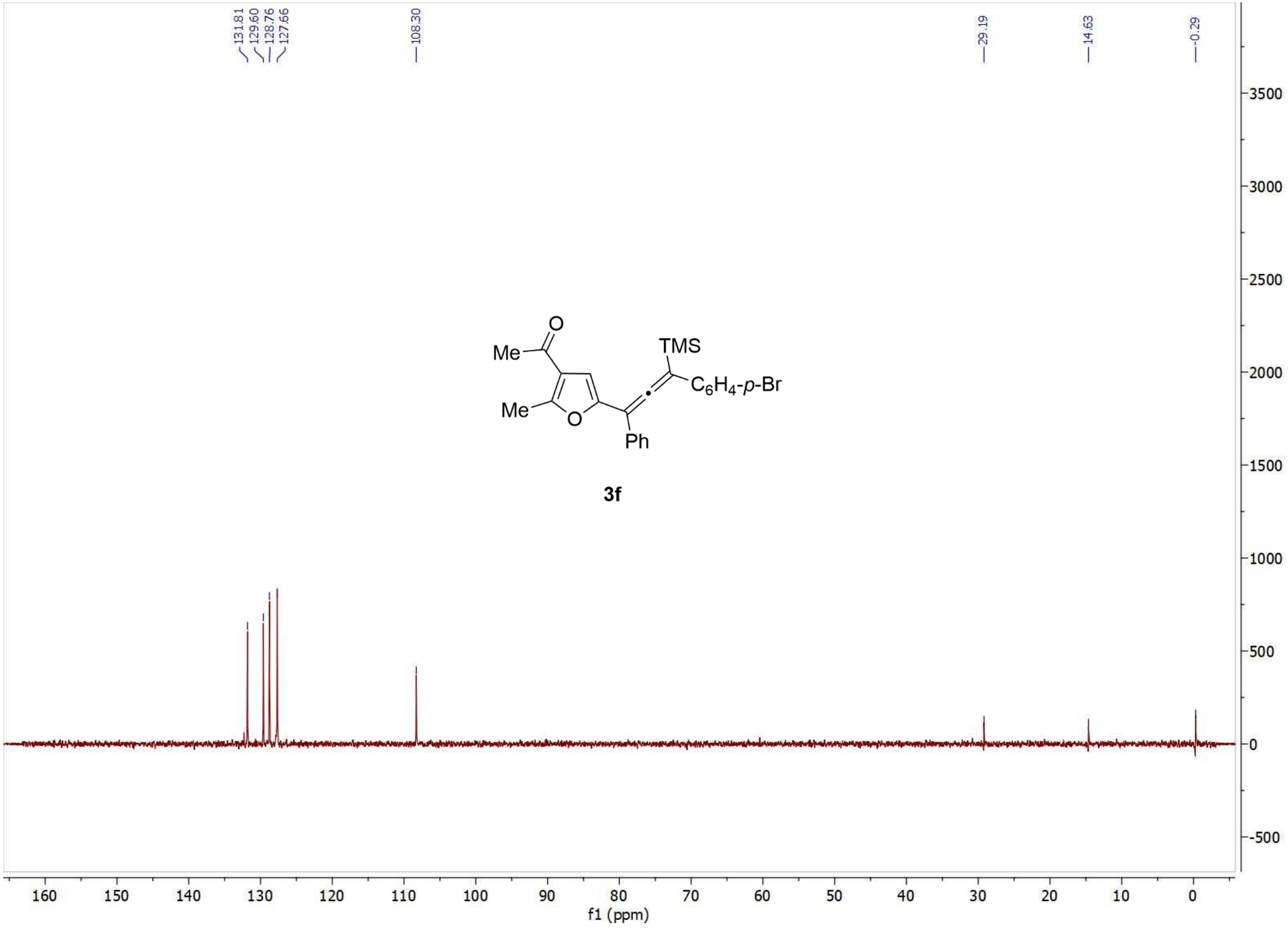

^{1}H NMR of compound 3g (300 MHz, $CDCl_3$)

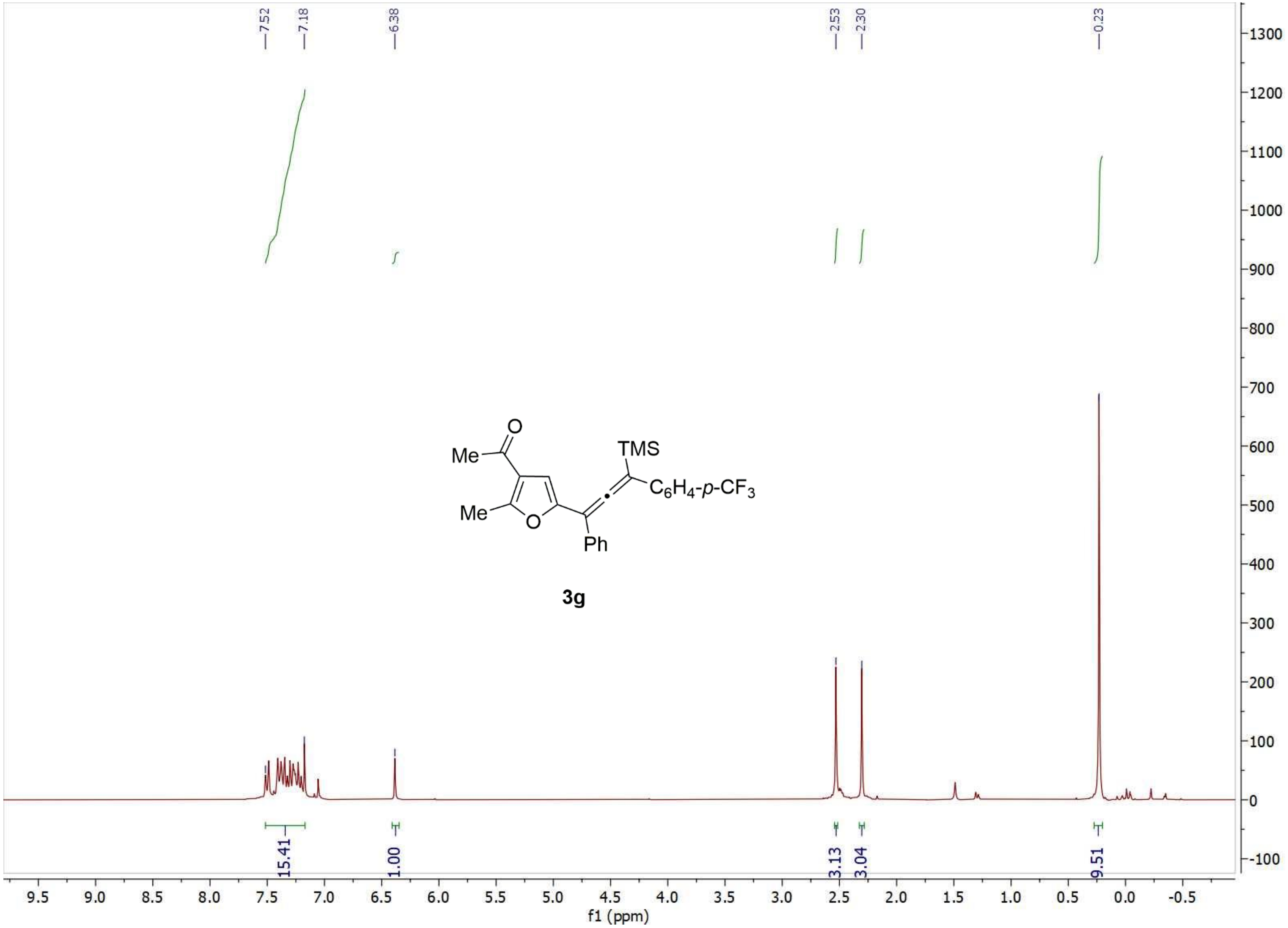

^{13}C NMR of compound 3g (150 MHz, $CDCl_3$)

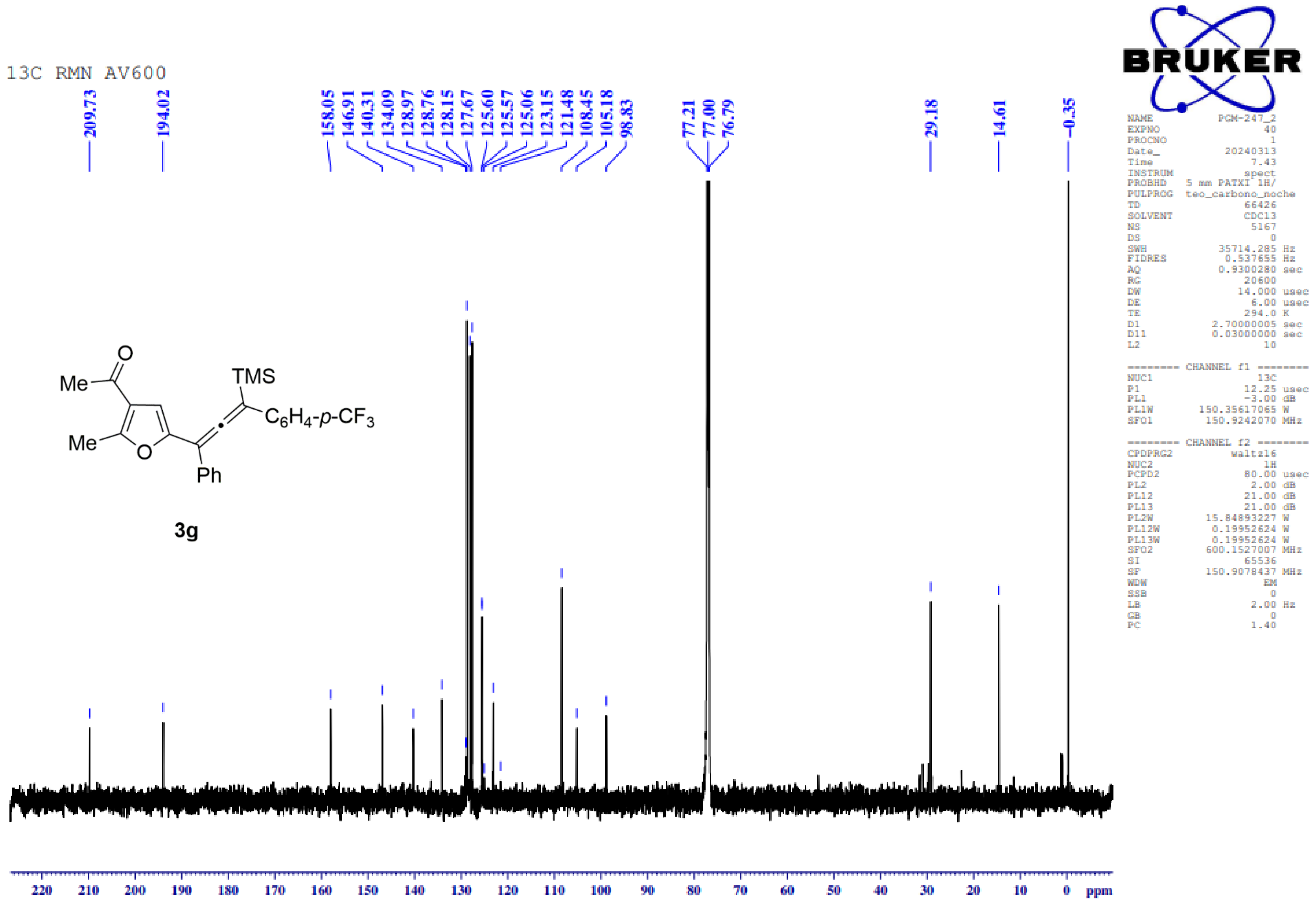

^{13}C NMR of compound 3g (150 MHz, $CDCl_3$)

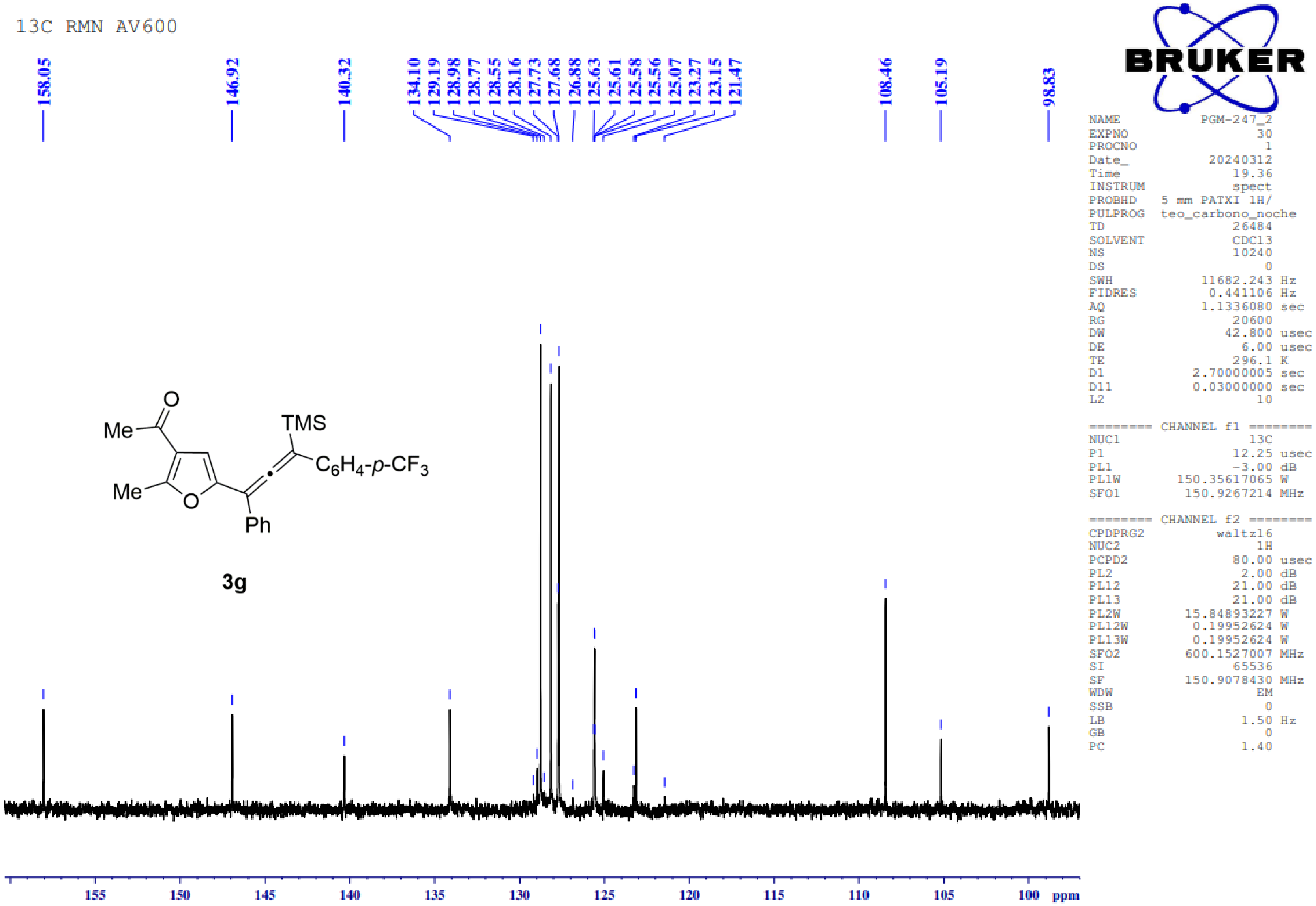

DEPT ^{13}C NMR of compound 3g (150 MHz, $CDCl_3$)

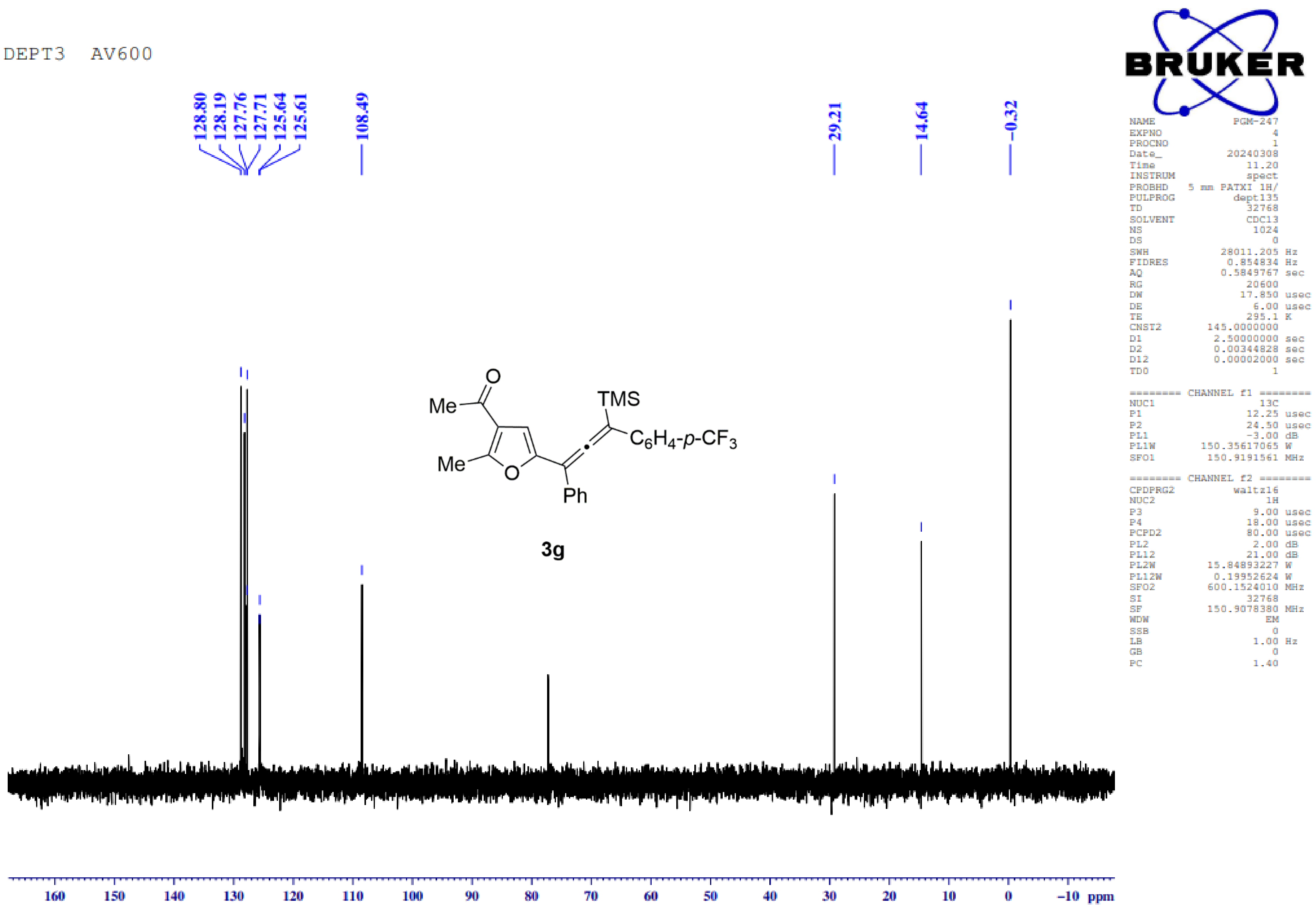

^{19}F NMR of compound 3g (282 MHz, $CDCl_3$)

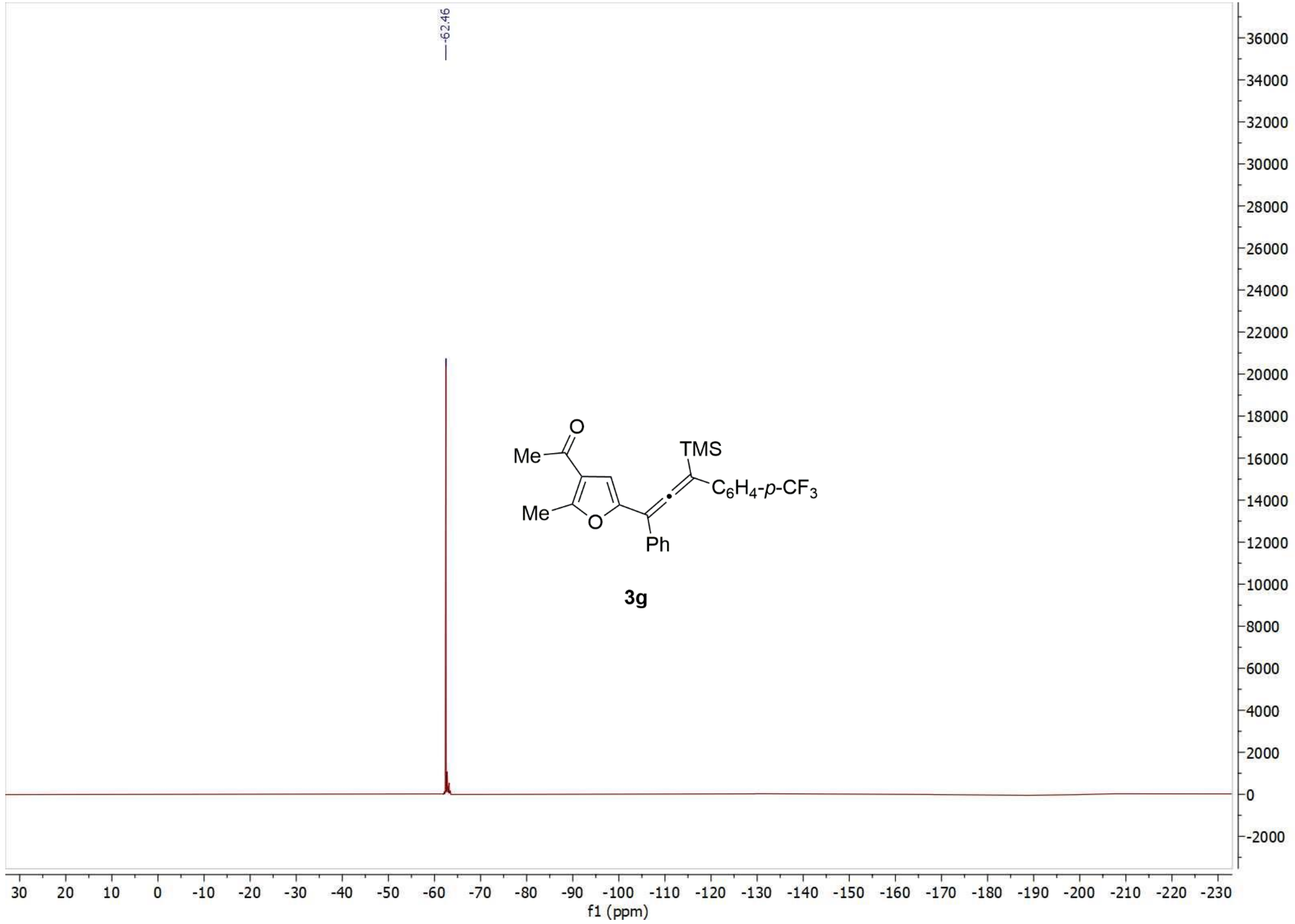

^{1}H NMR of compound 3h (300 MHz, $CDCl_3$)

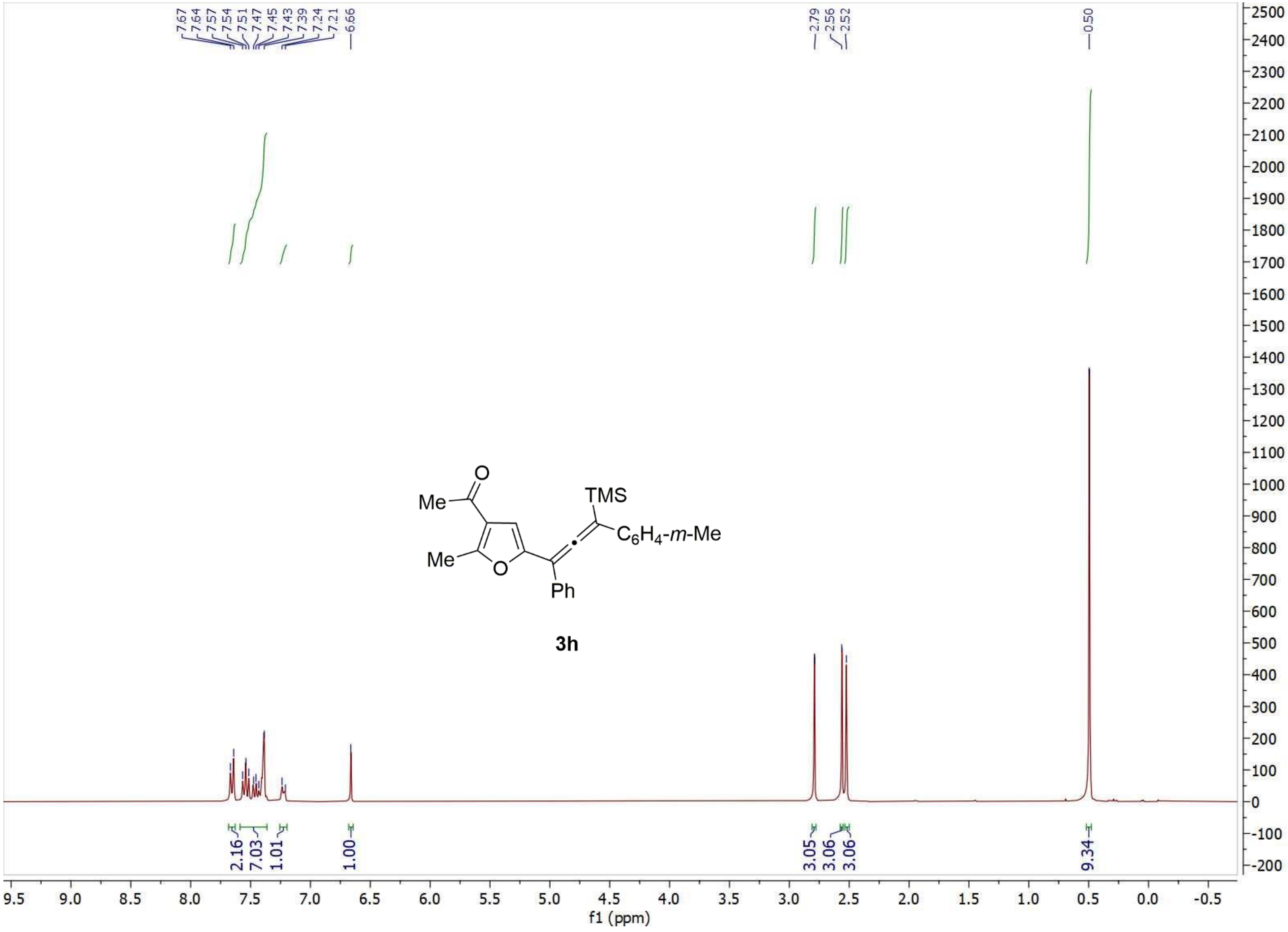

^{13}C NMR of compound 3h (75 MHz, $CDCl_3$)

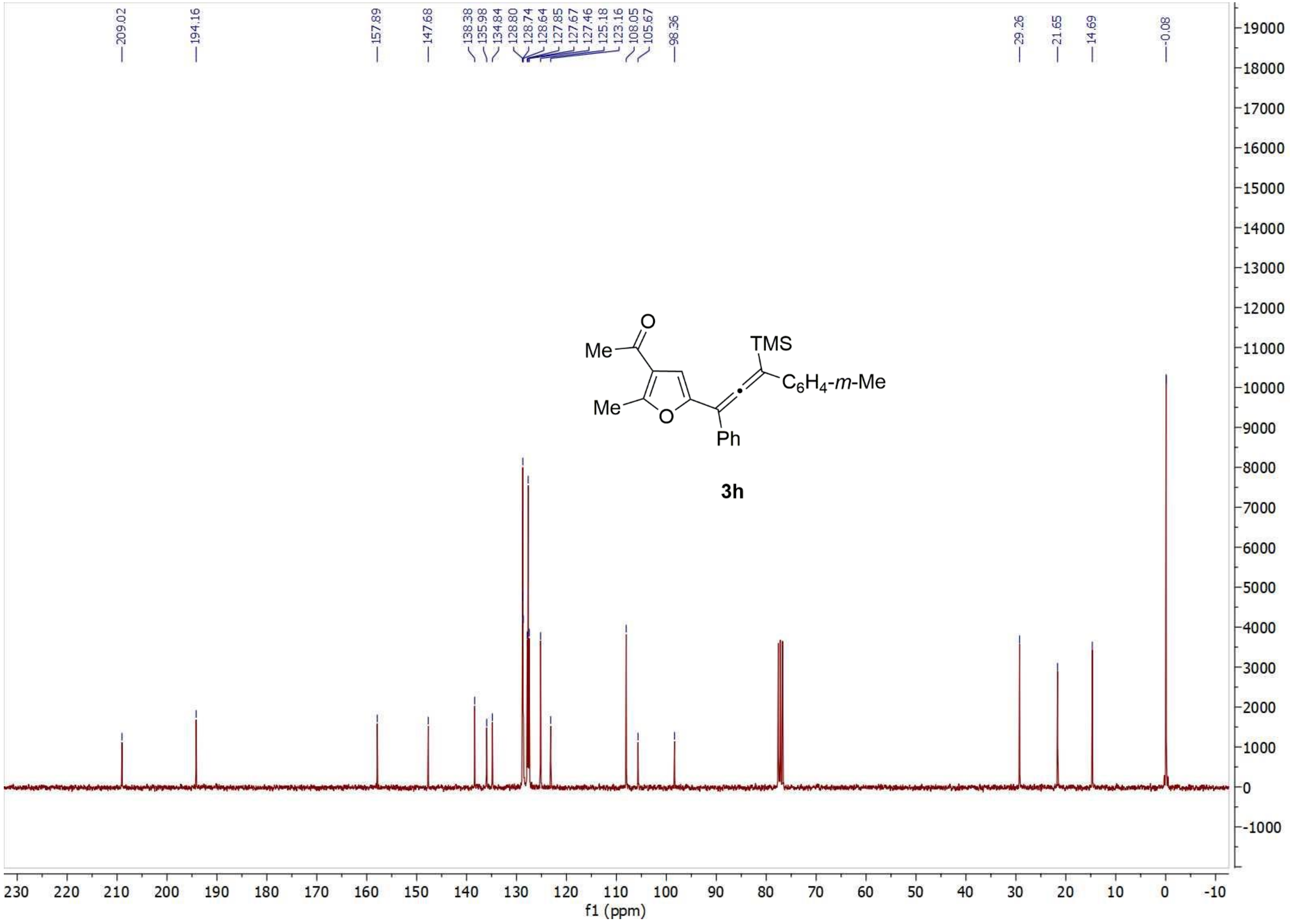

DEPT ^{13}C NMR of compound 3h (75 MHz, $CDCl_3$)

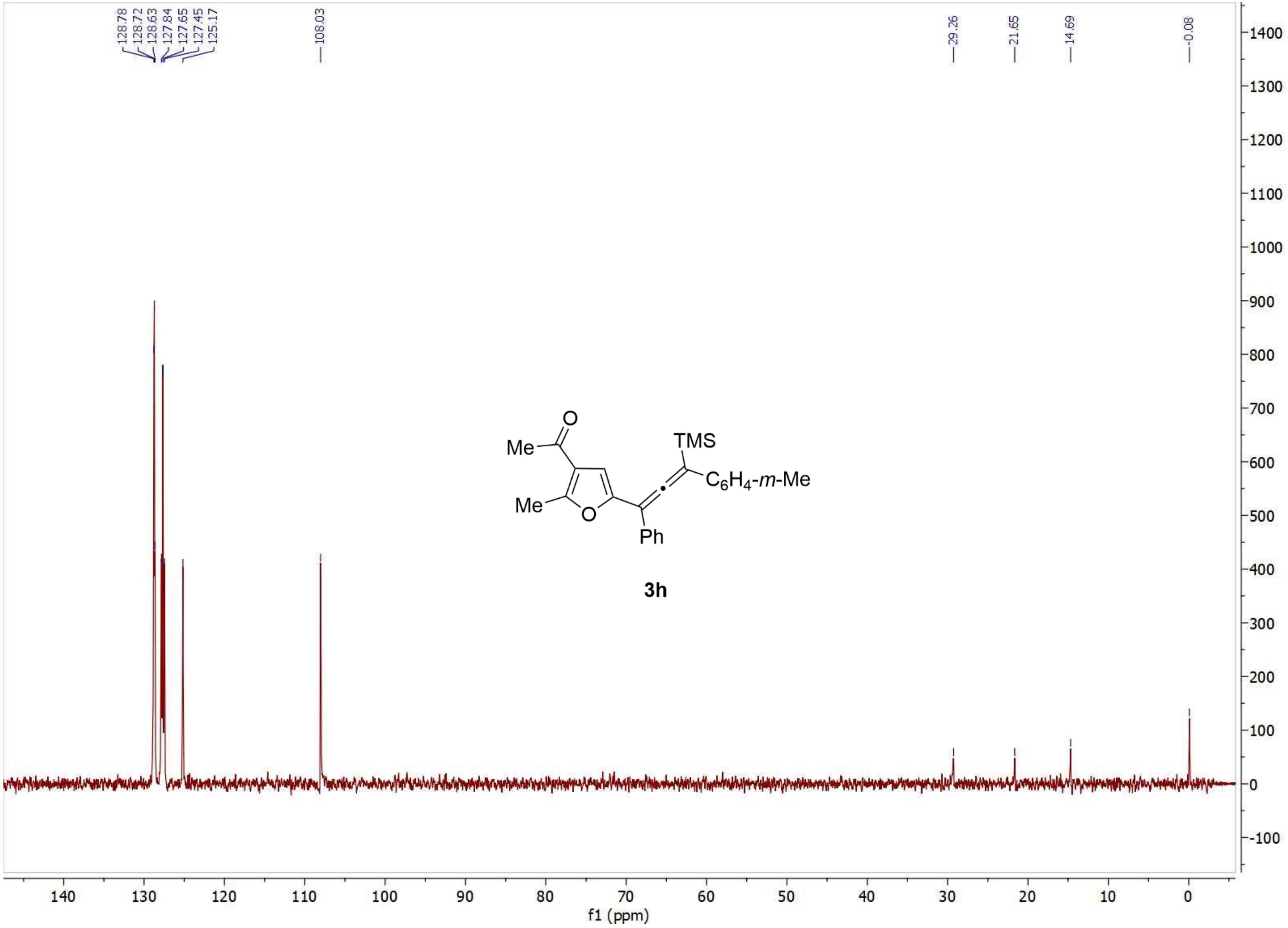

^{1}H NMR of compound 3i (300 MHz, $CDCl_3$)

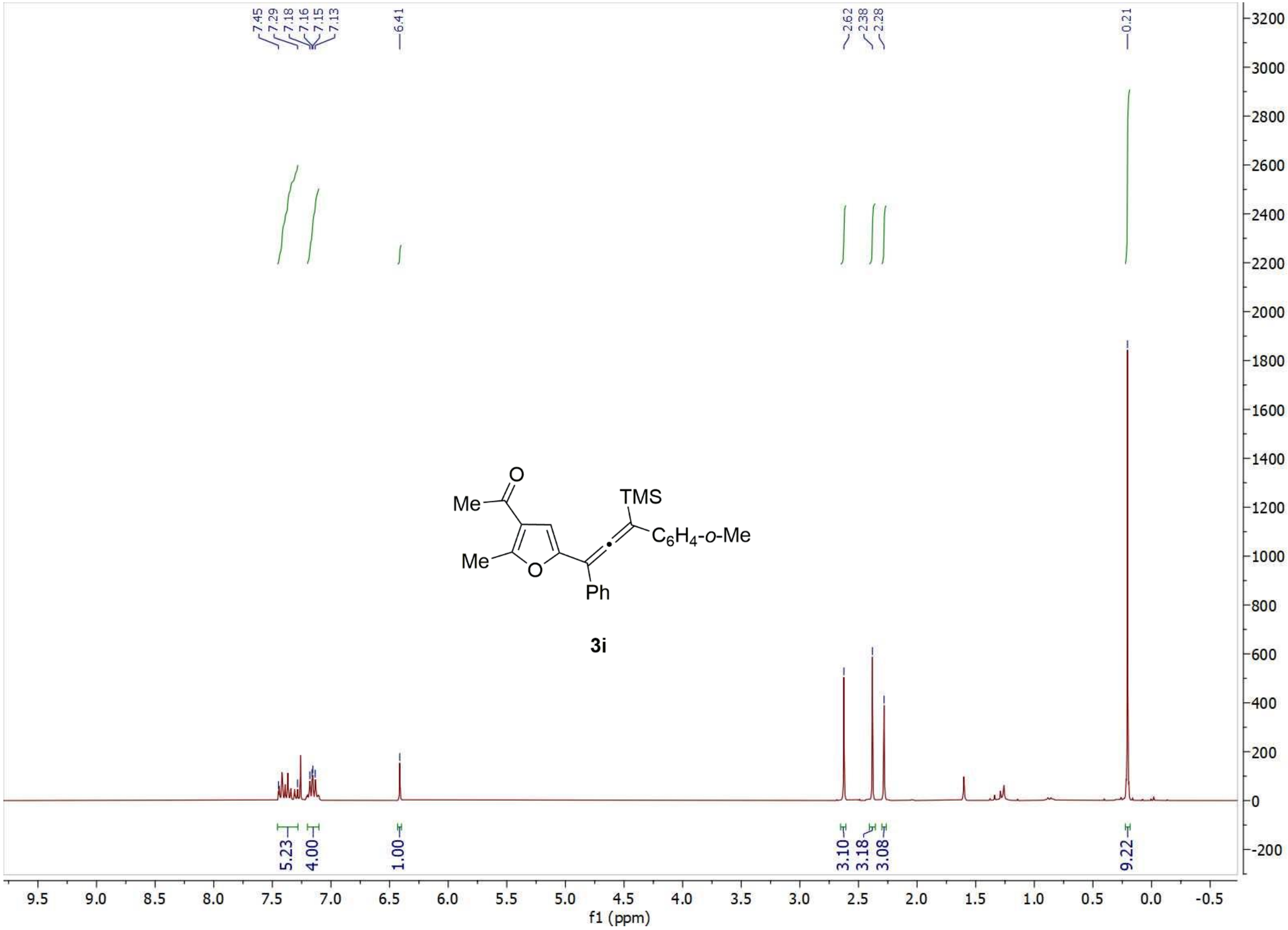

^{13}C NMR of compound 3i (75 MHz, $CDCl_3$)

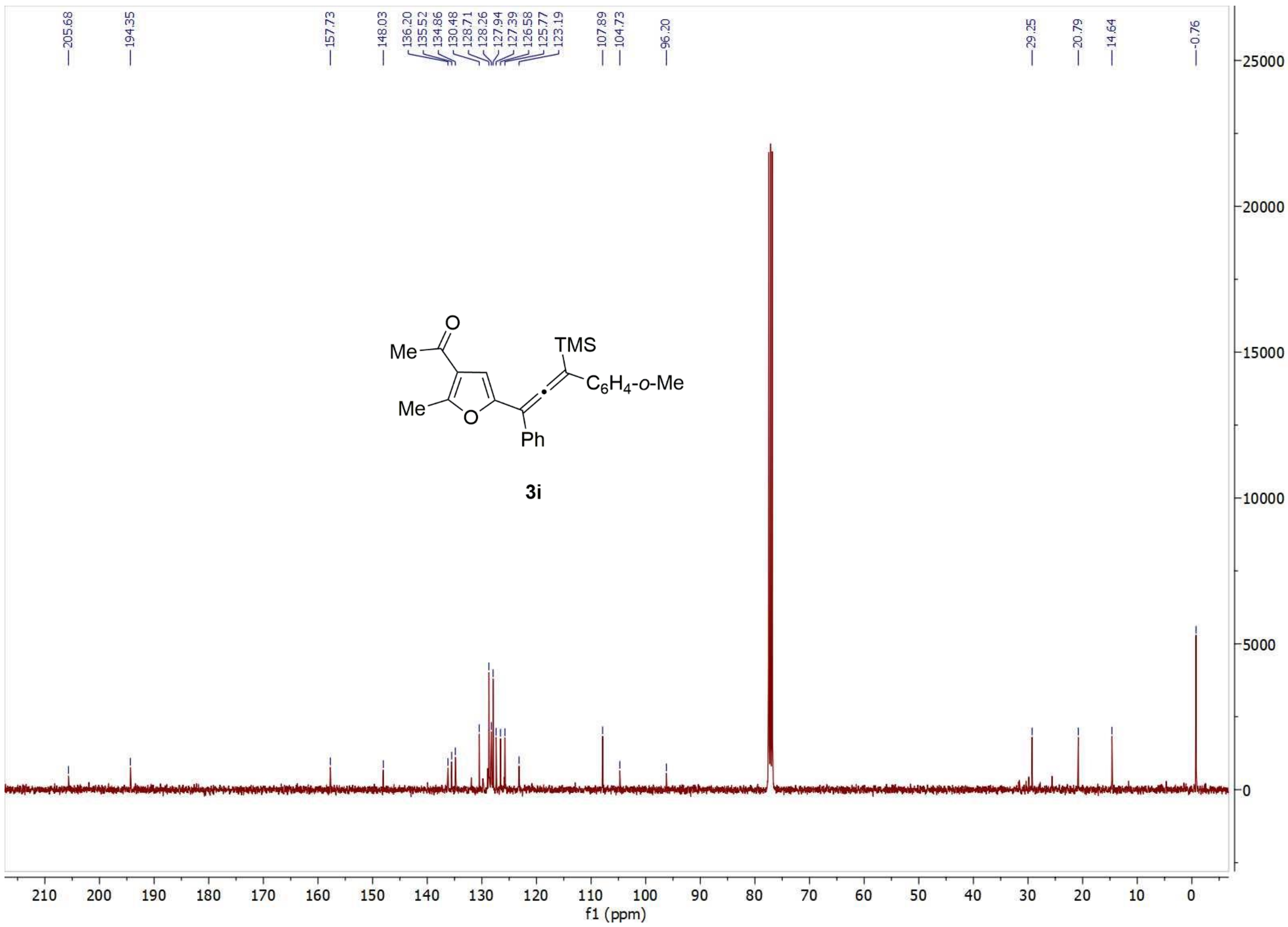

DEPT ^{13}C NMR of compound 3i (75 MHz, $CDCl_3$)

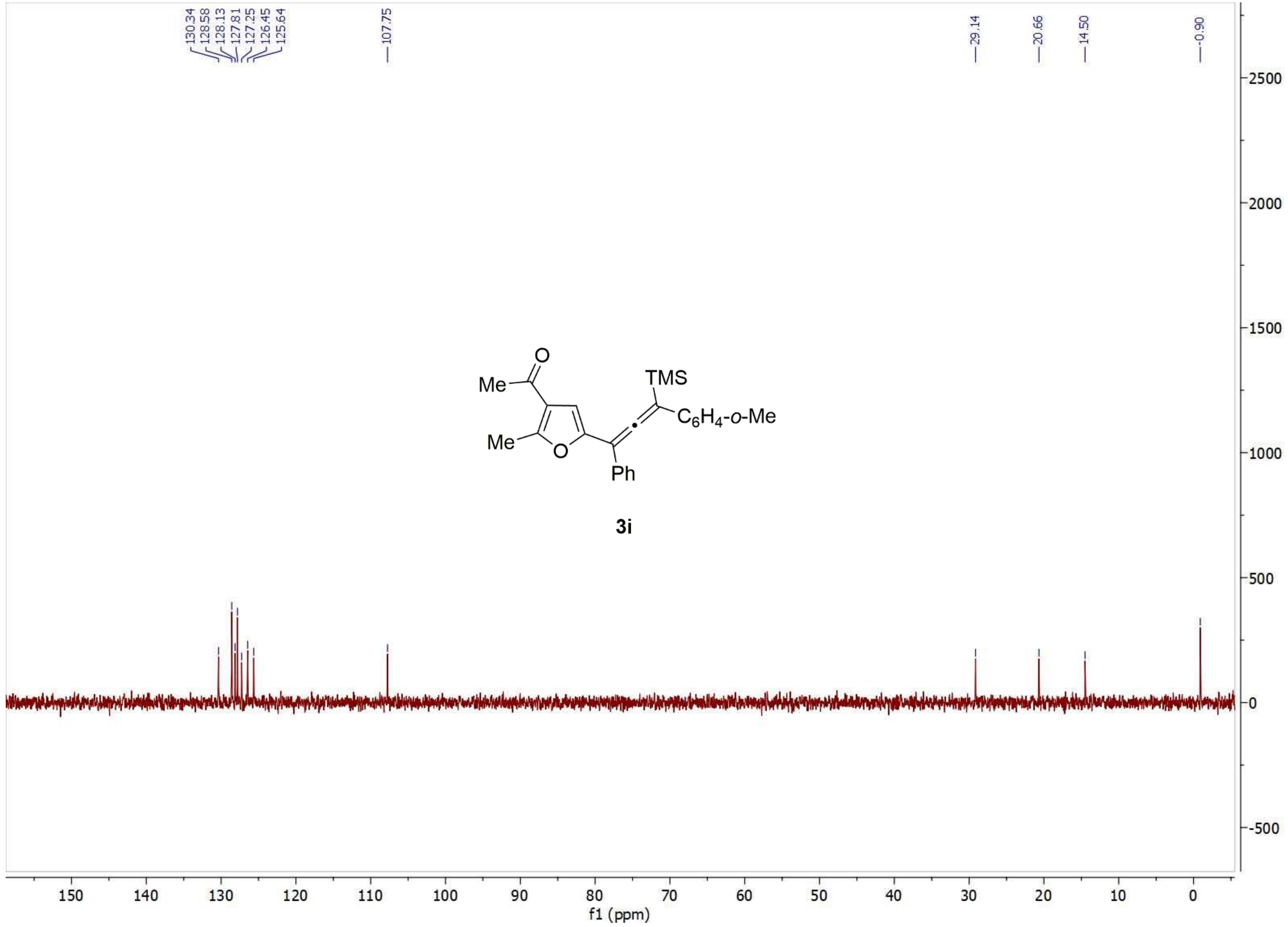

^{1}H NMR of compound 3j (300 MHz, $CDCl_3$)

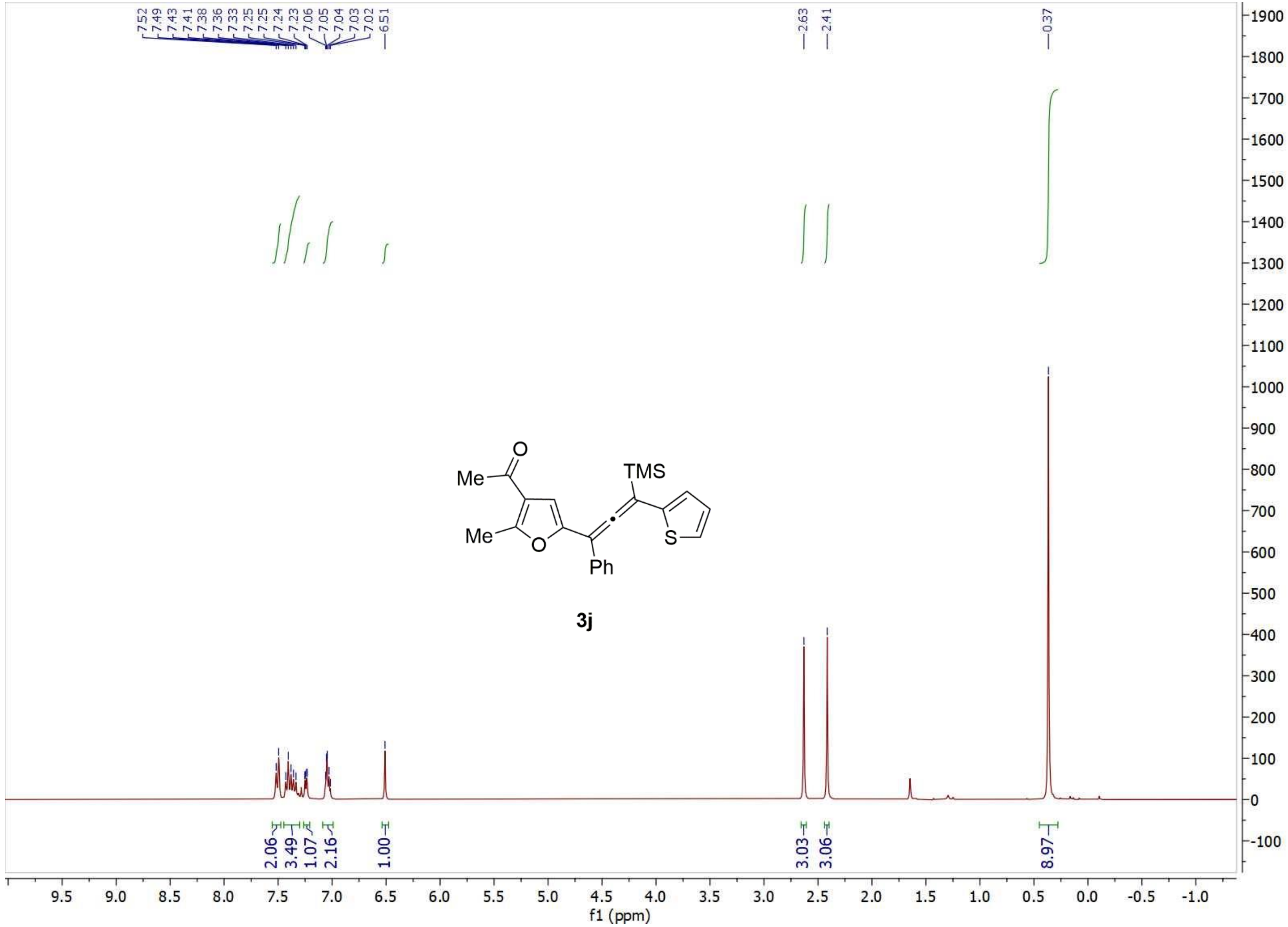

^{13}C NMR of compound 3j (75 MHz, $CDCl_3$)

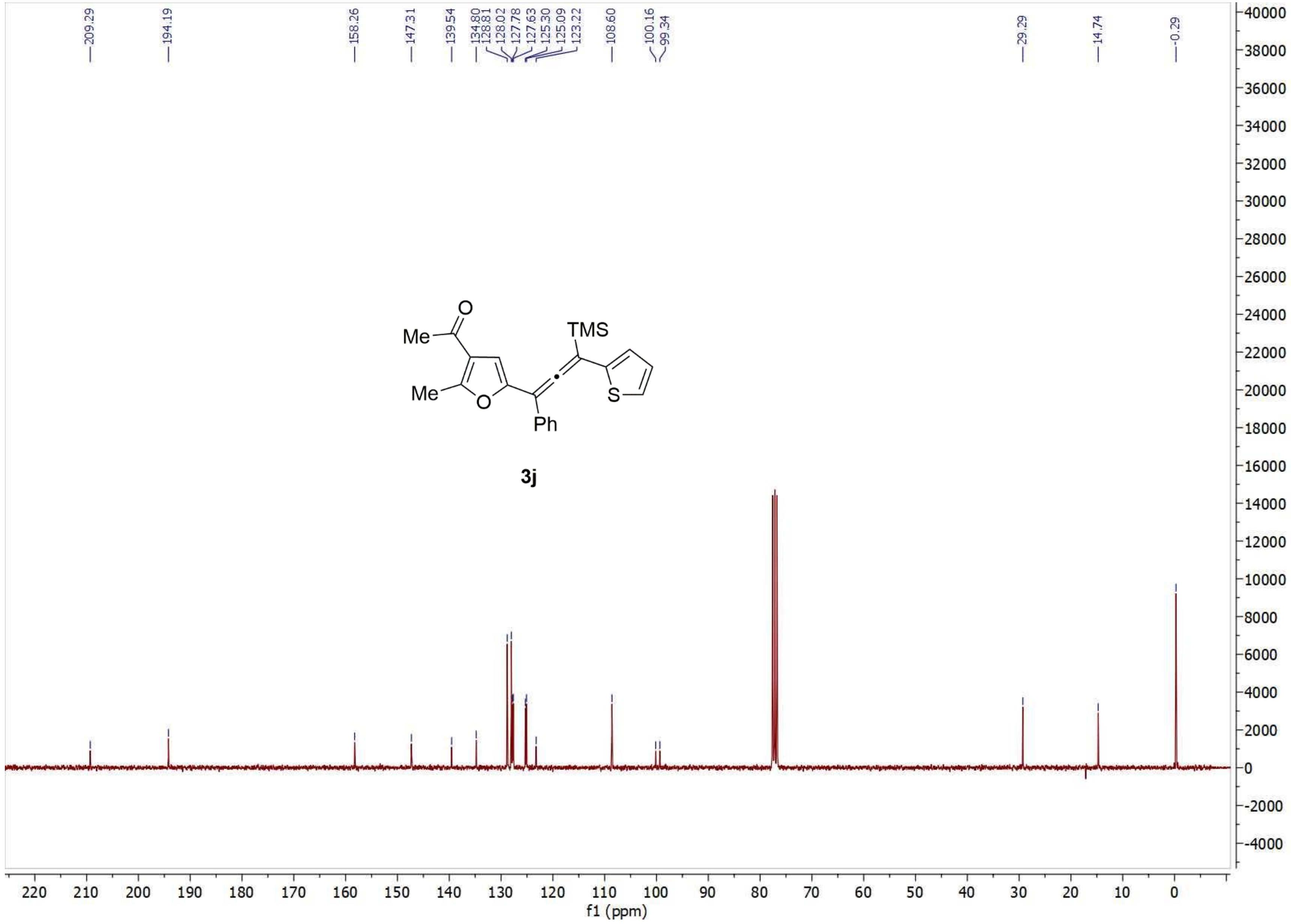

DEPT ^{13}C NMR of compound 3j (75 MHz, $CDCl_3$)

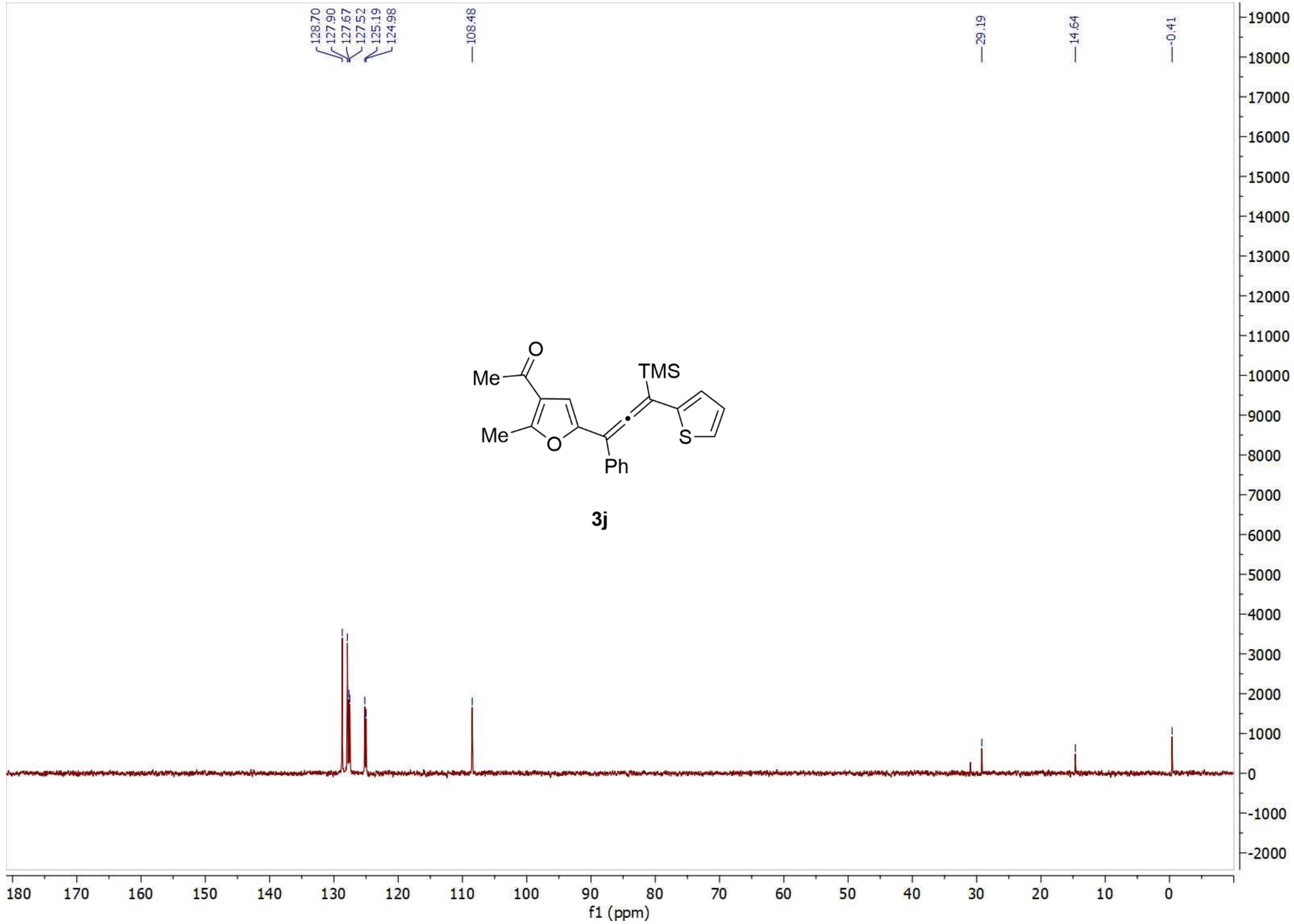

^{1}H NMR of compound 3k (300 MHz, $CDCl_3$)

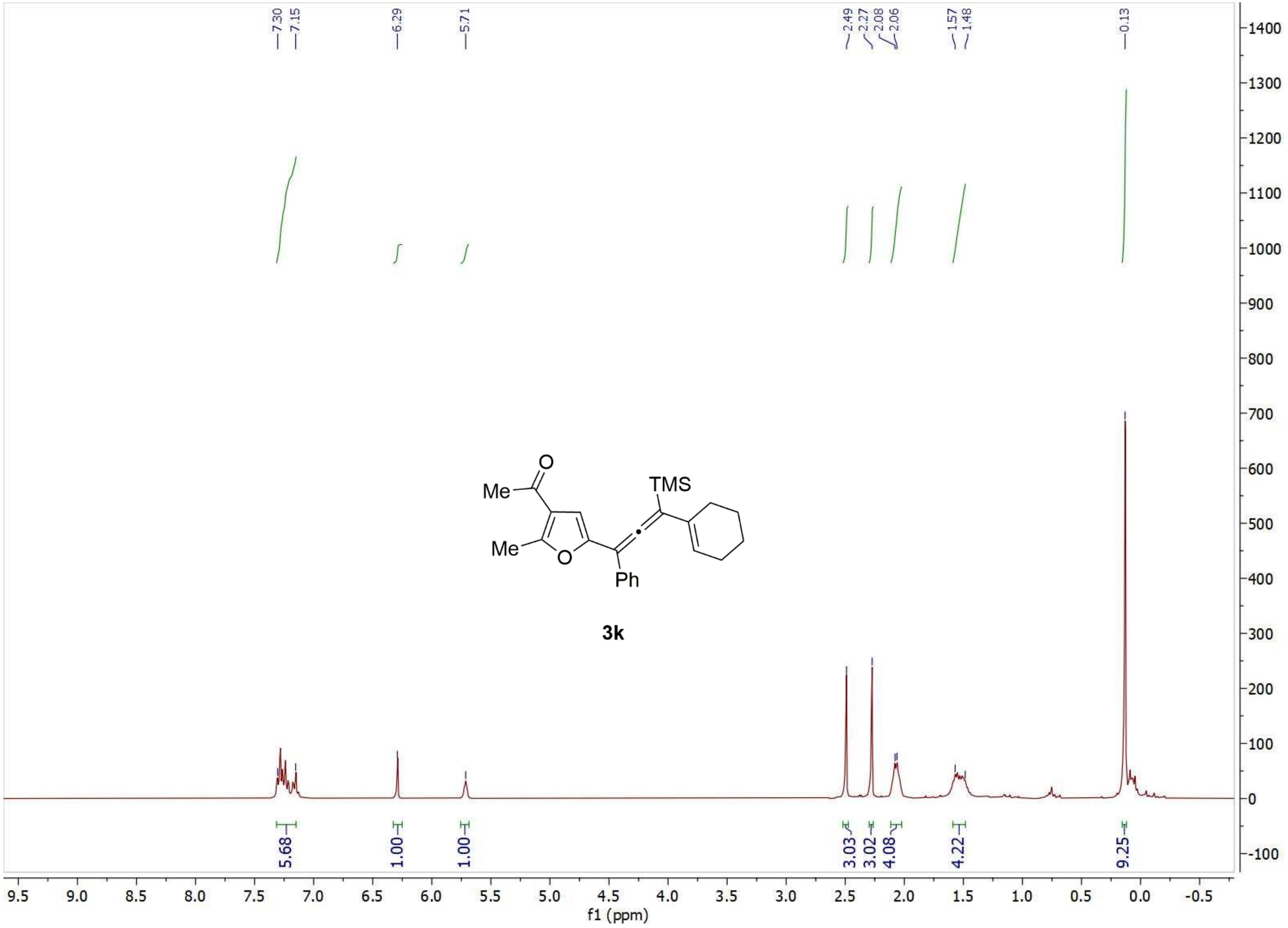

^{13}C NMR of compound 3k (75 MHz, $CDCl_3$)

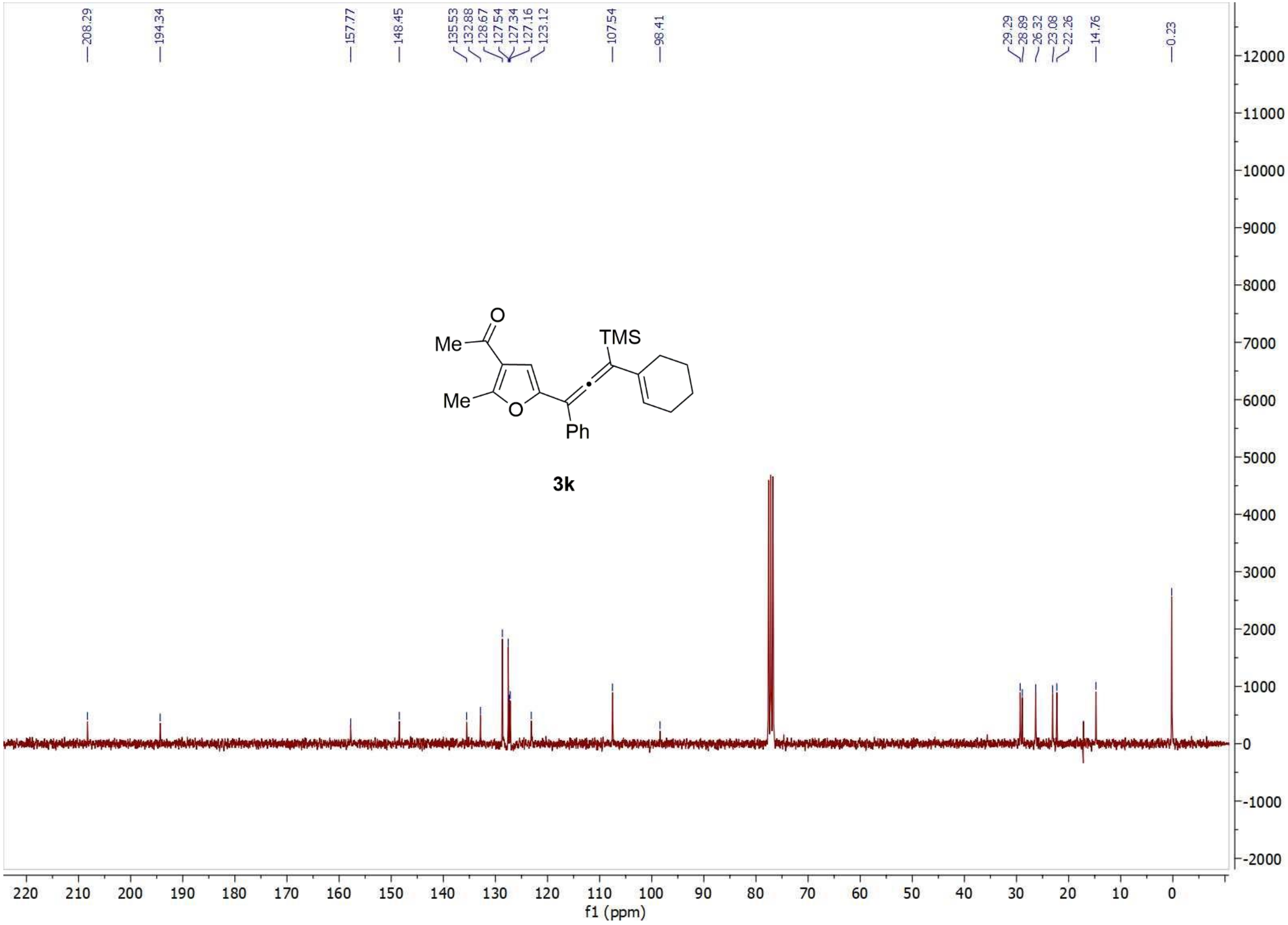

DEPT ^{13}C NMR of compound 3k (75 MHz, $CDCl_3$)

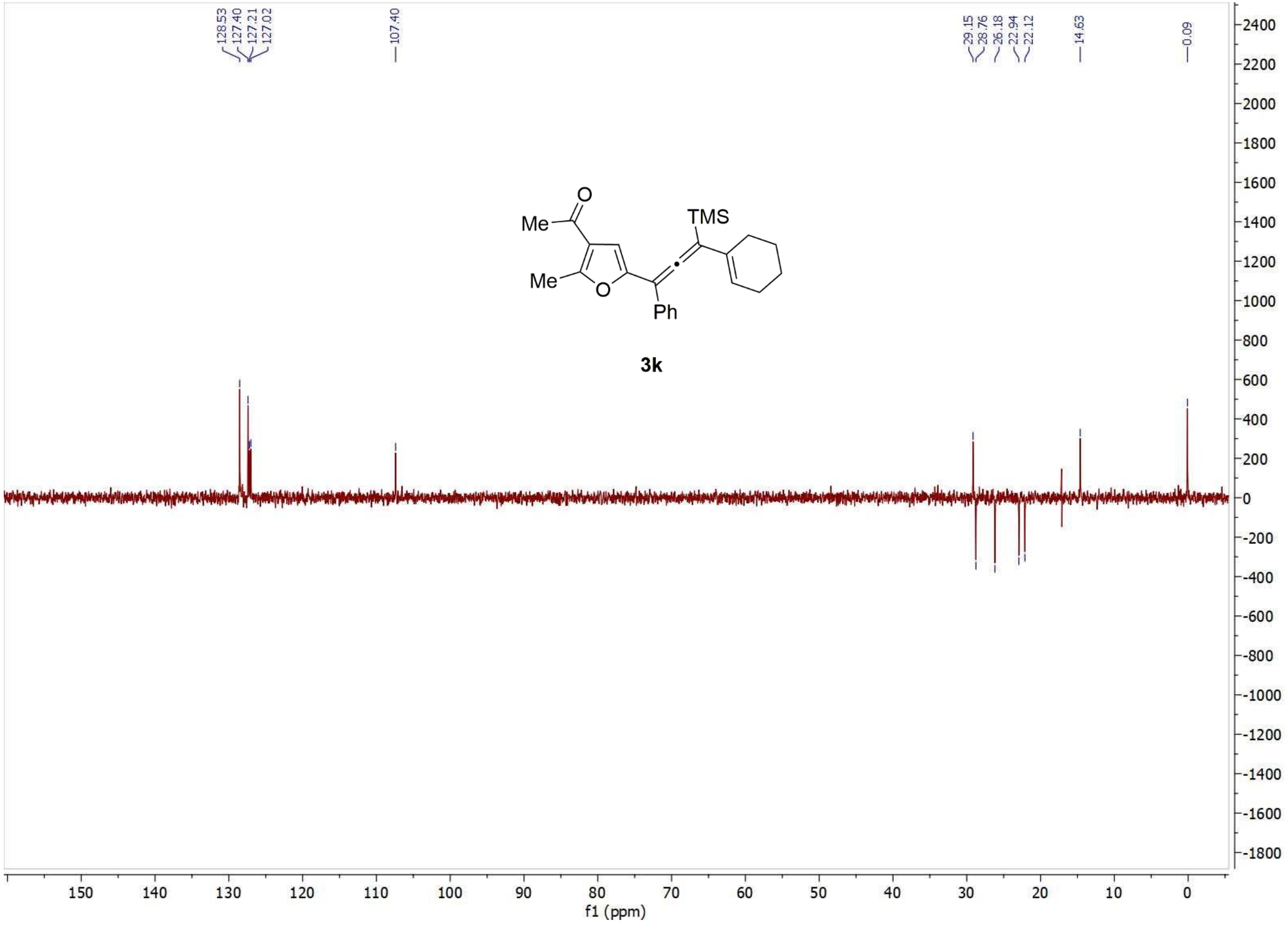

^{1}H NMR of compound 3l (300 MHz, $CDCl_3$)

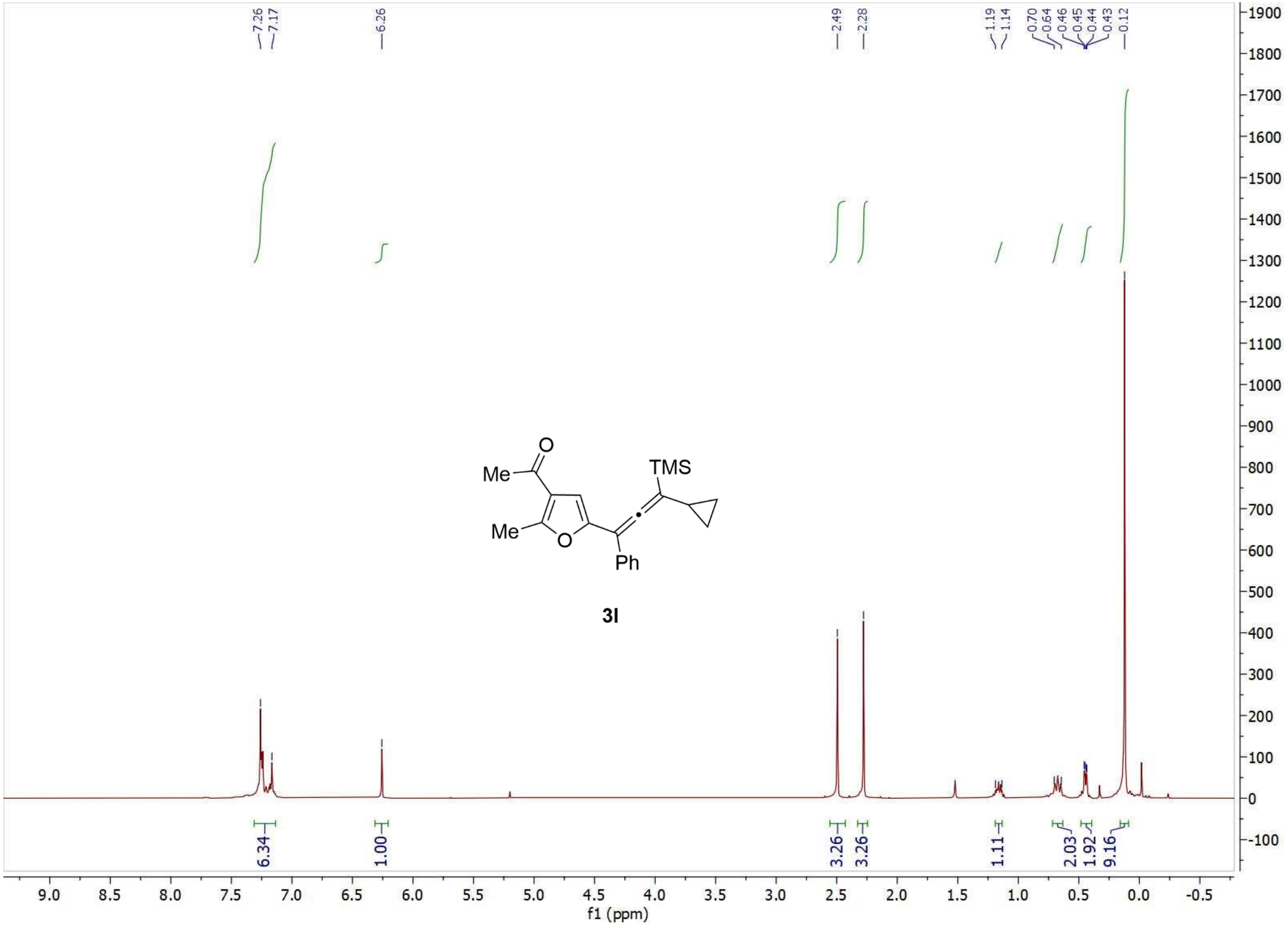

^{13}C NMR of compound 3l (75 MHz, $CDCl_3$)

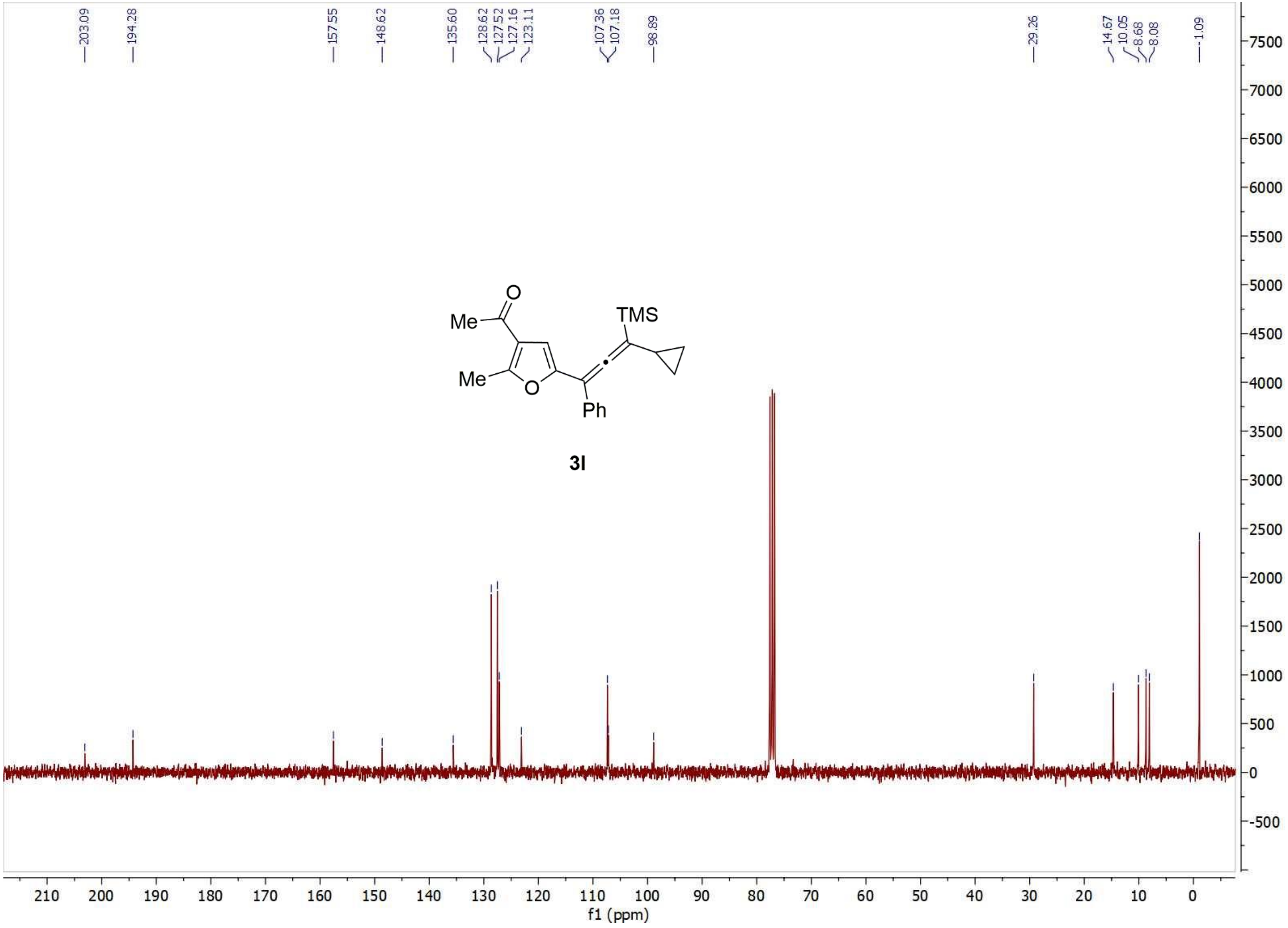

DEPT ^{13}C NMR of compound 3l (75 MHz, $CDCl_3$)

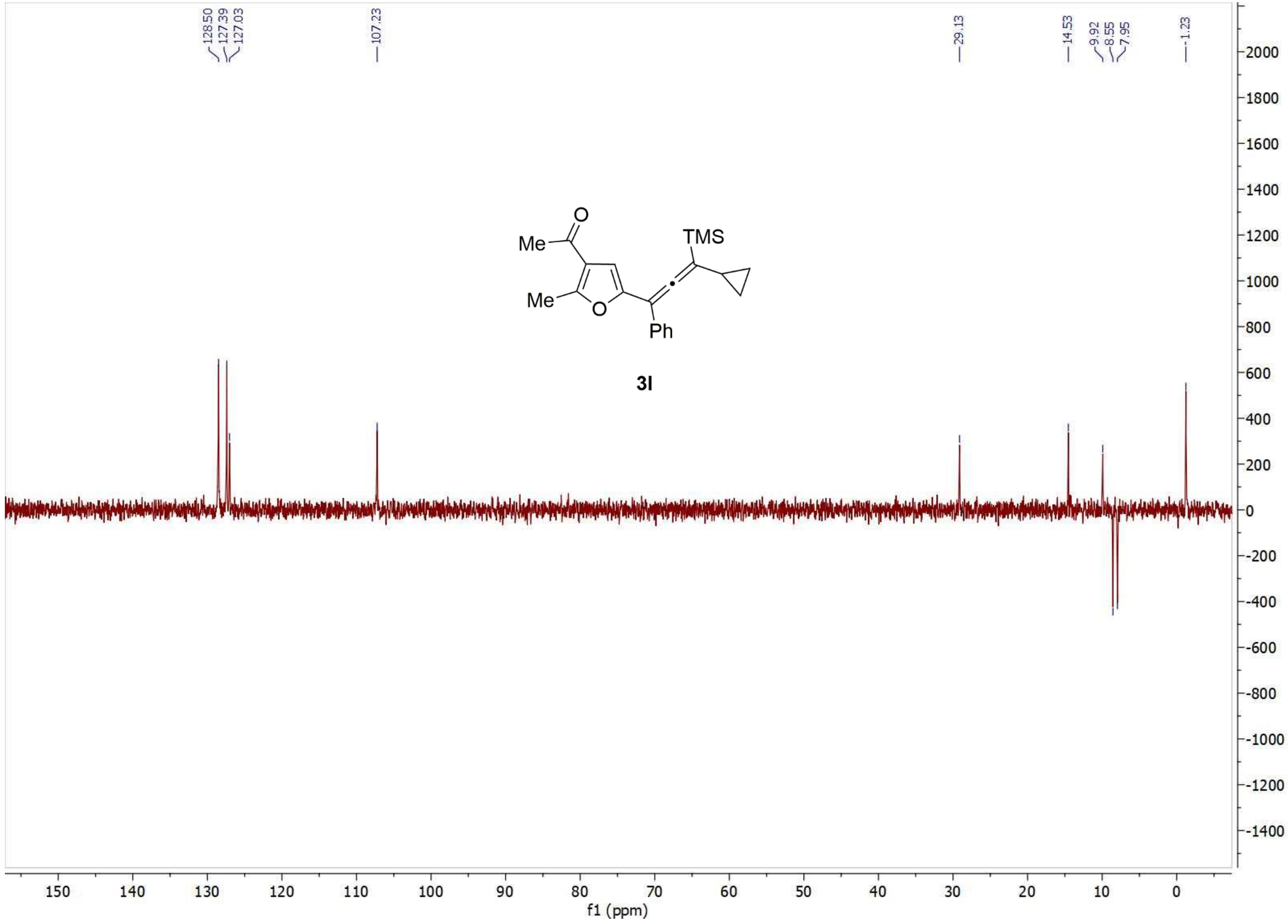

^{1}H NMR of compound 3m (300 MHz, $CDCl_3$)

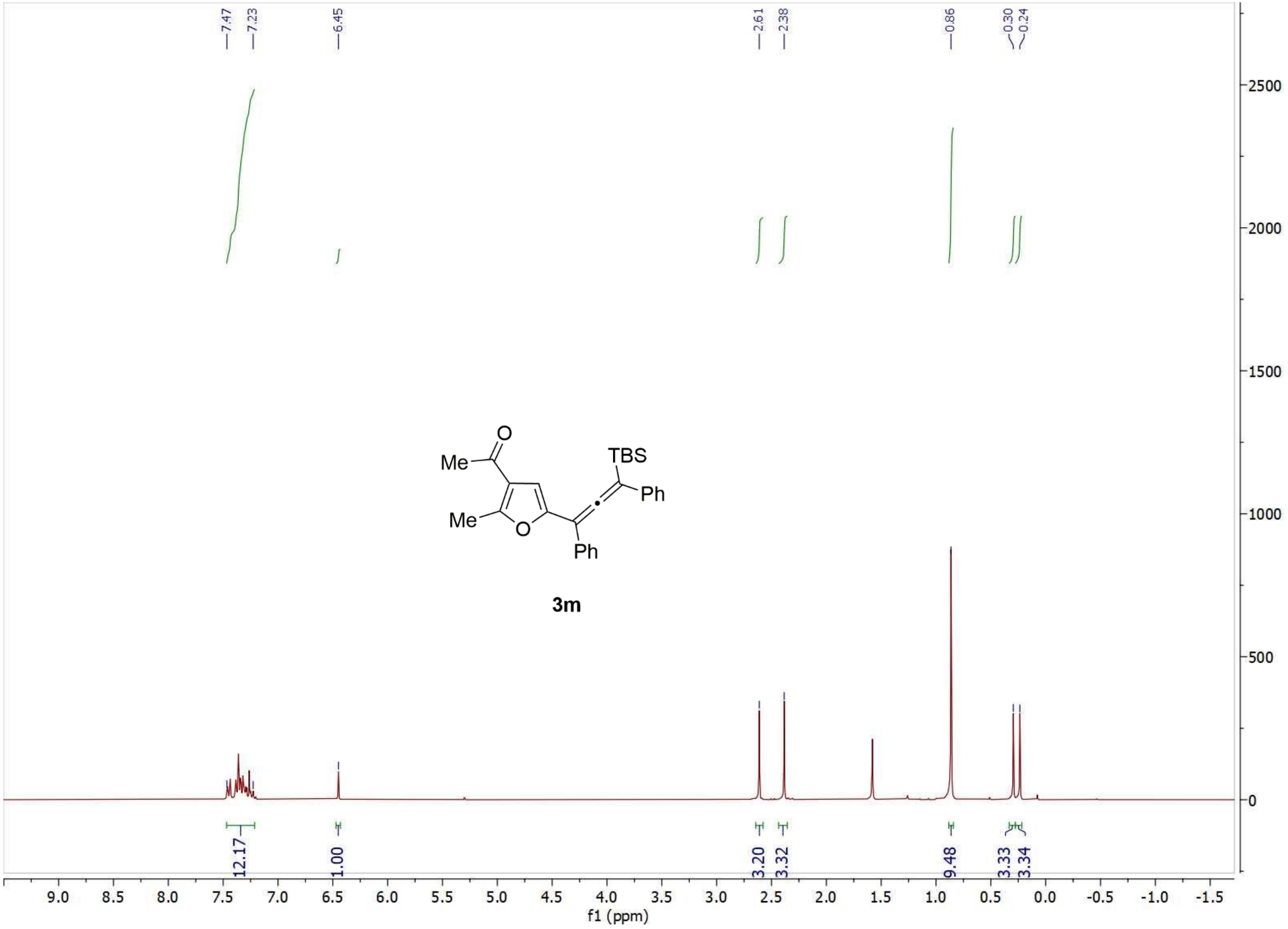

^{13}C NMR of compound 3m (75 MHz, $CDCl_3$)

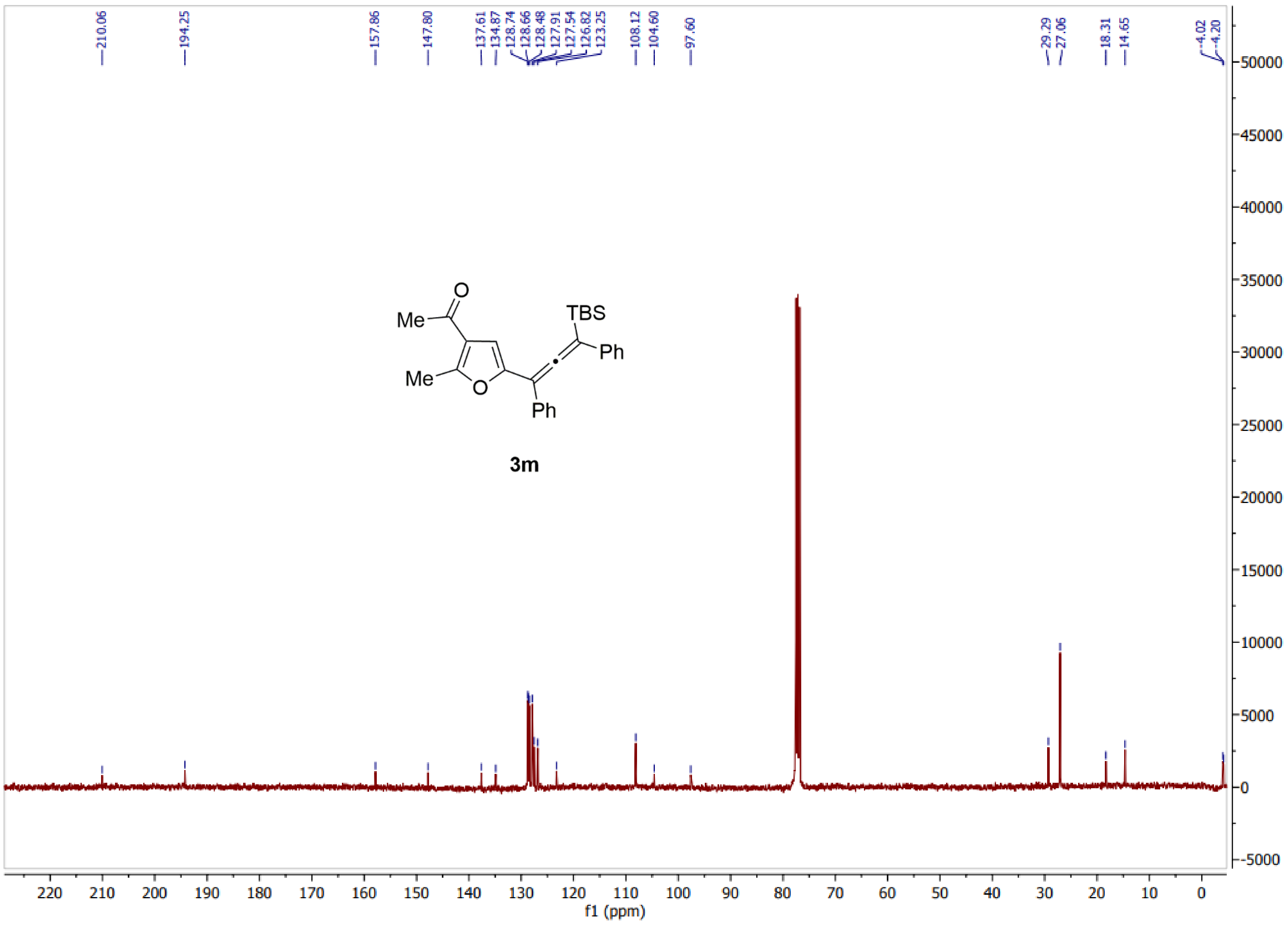

DEPT ^{13}C NMR of compound 3m (75 MHz, $CDCl_3$)

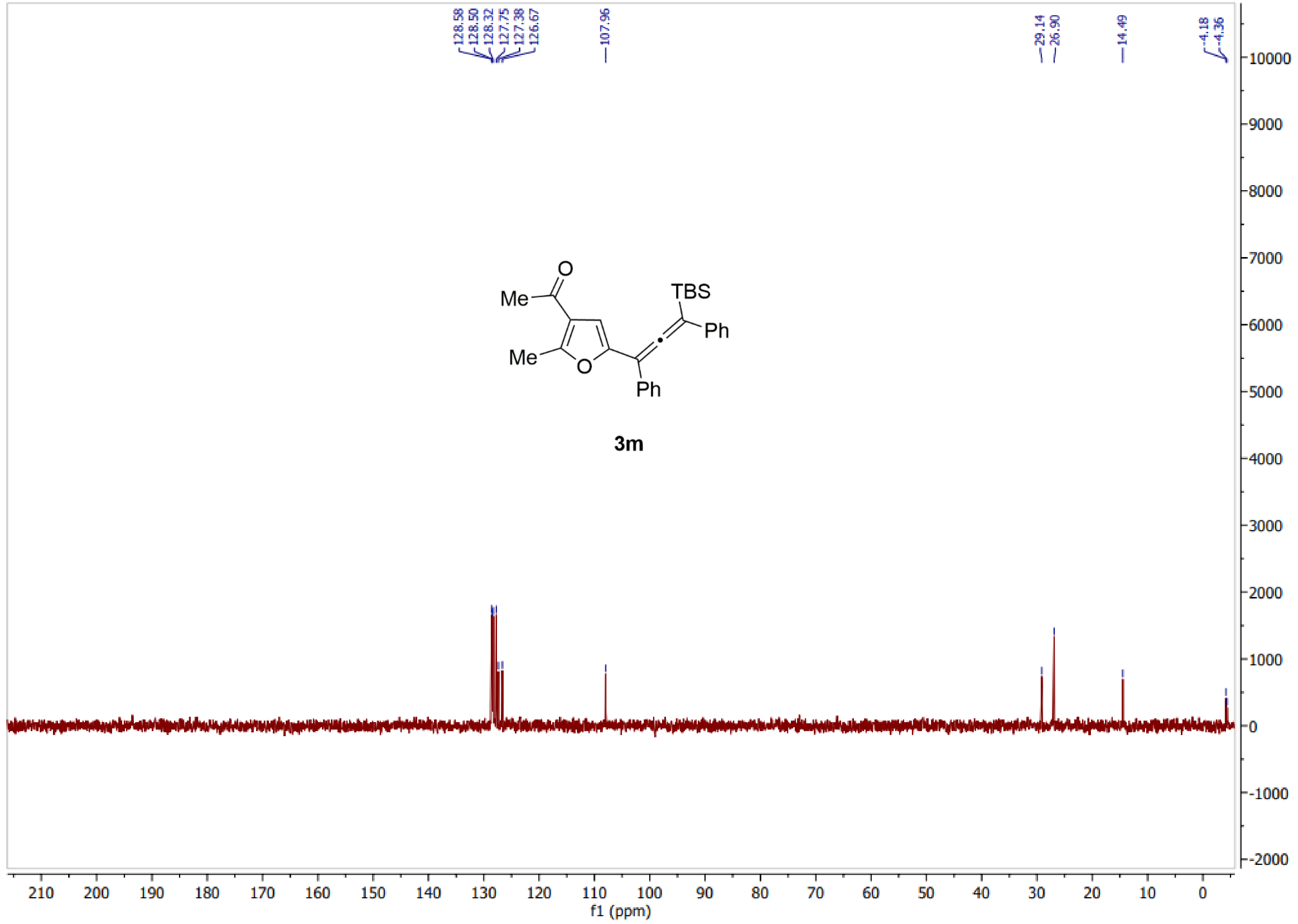

^{1}H NMR of compound 3n (300 MHz, $CDCl_3$)

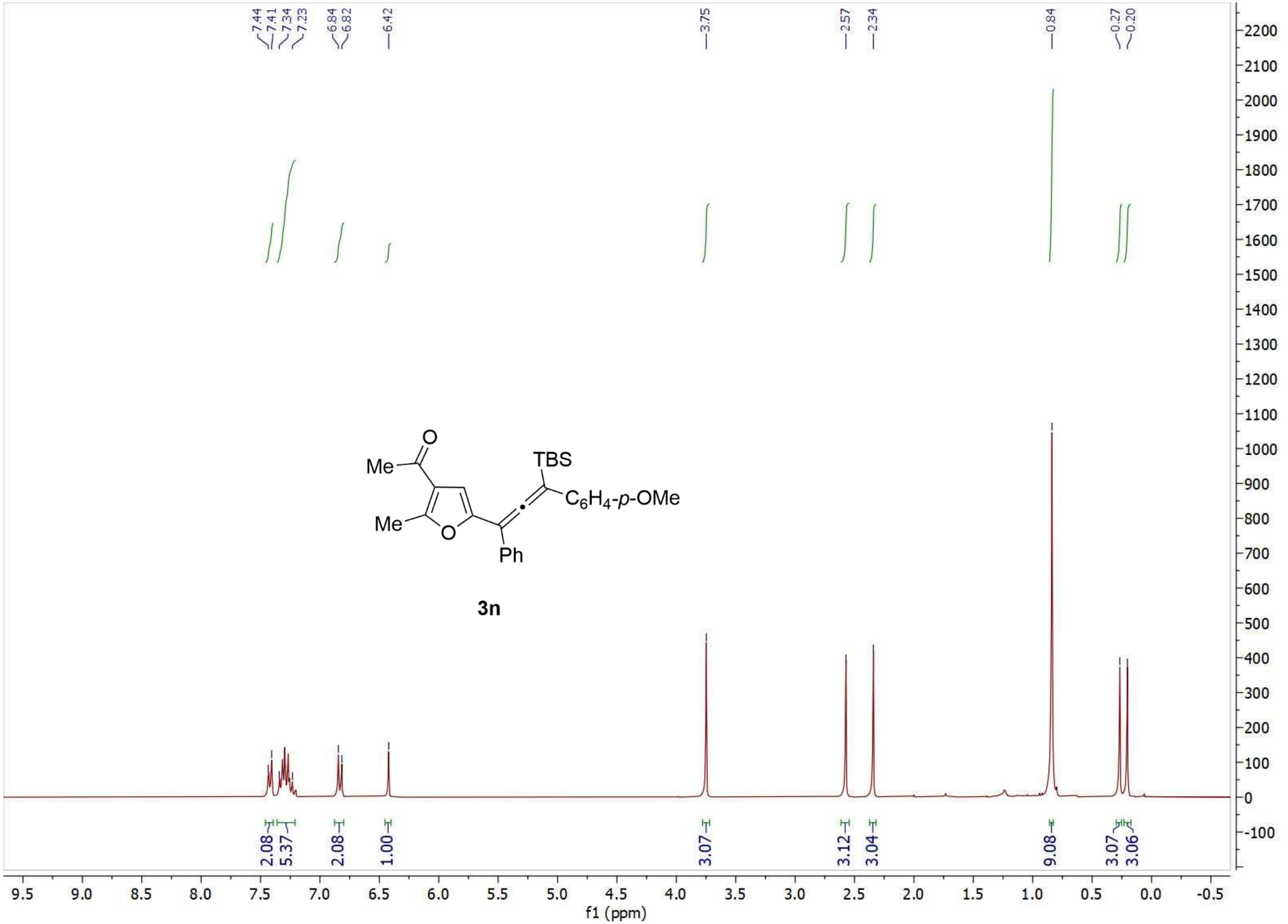

^{13}C NMR of compound 3n (75 MHz, $CDCl_3$)

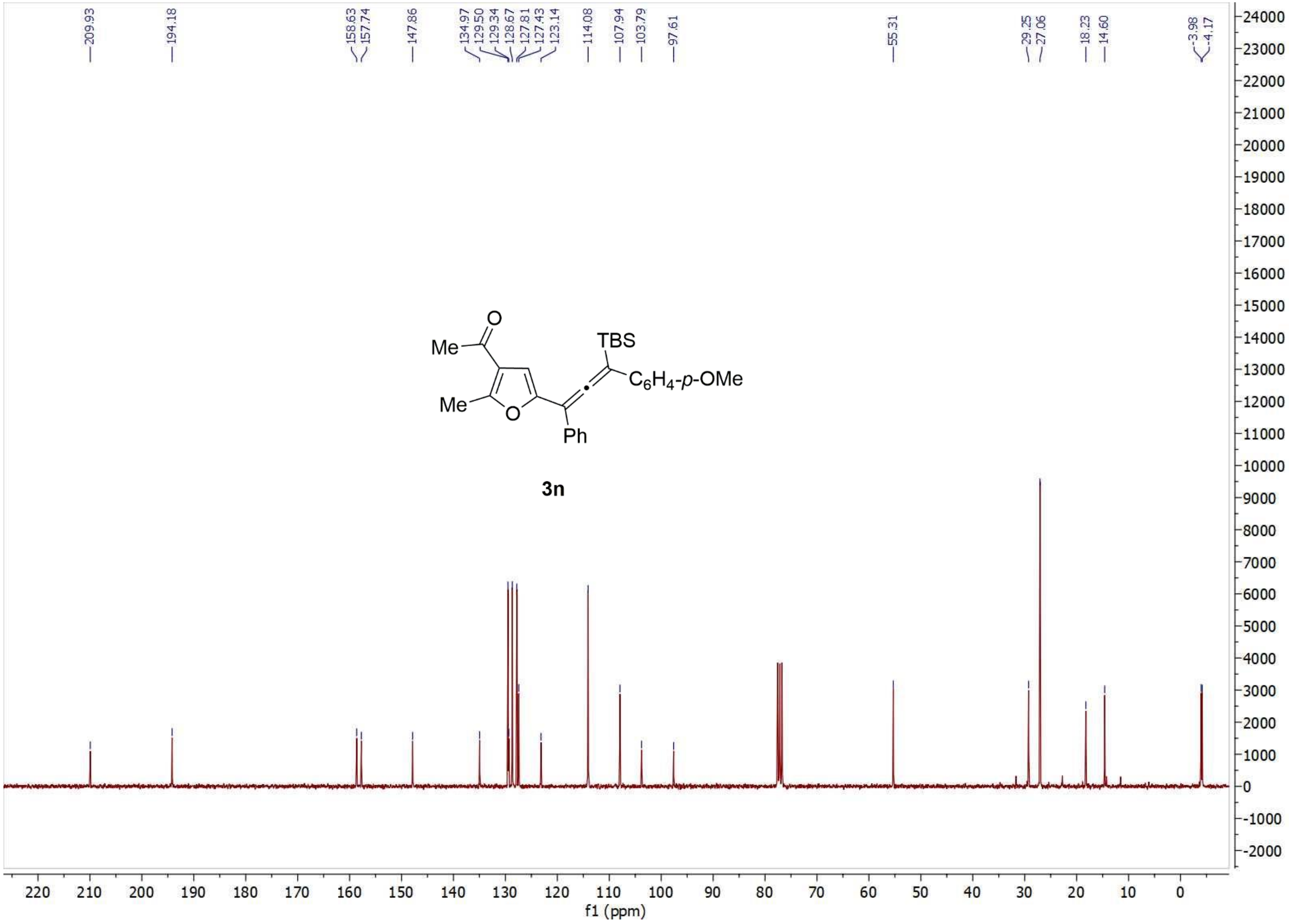

DEPT ^{13}C NMR of compound 3n (75 MHz, $CDCl_3$)

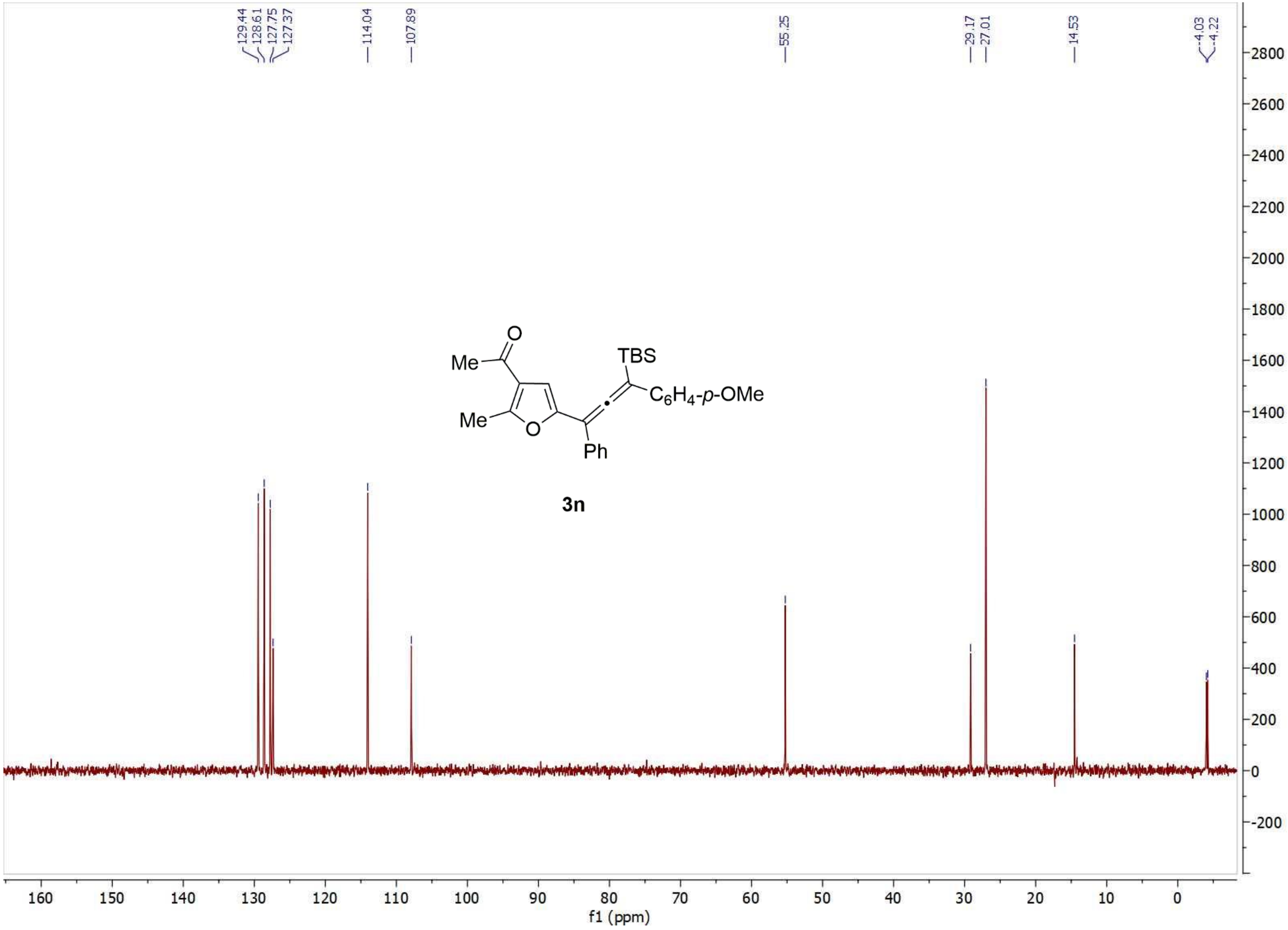

^{1}H NMR of compound 3o (300 MHz, $CDCl_3$)

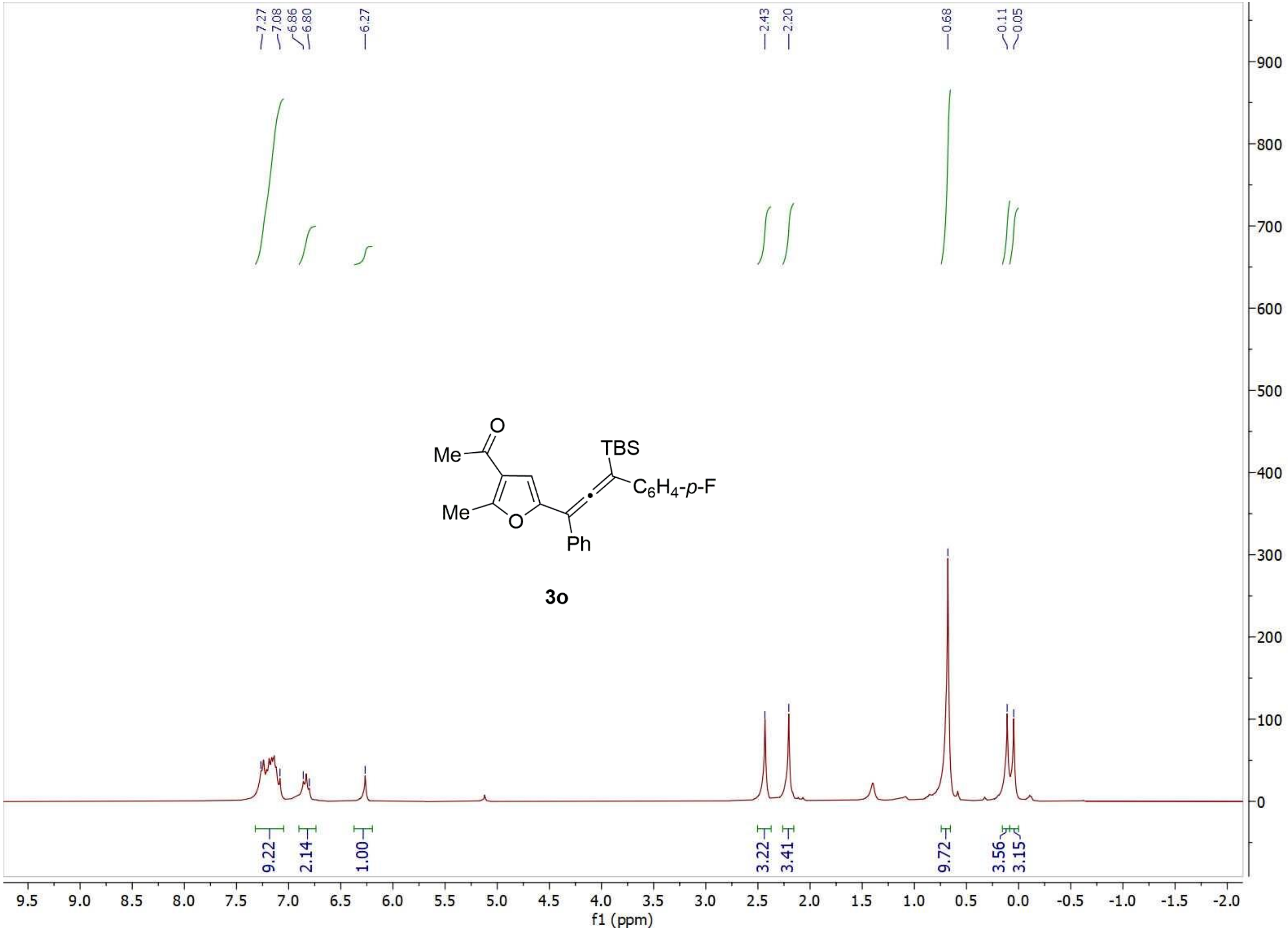

^{13}C NMR of compound 3o (75 MHz, $CDCl_3$)

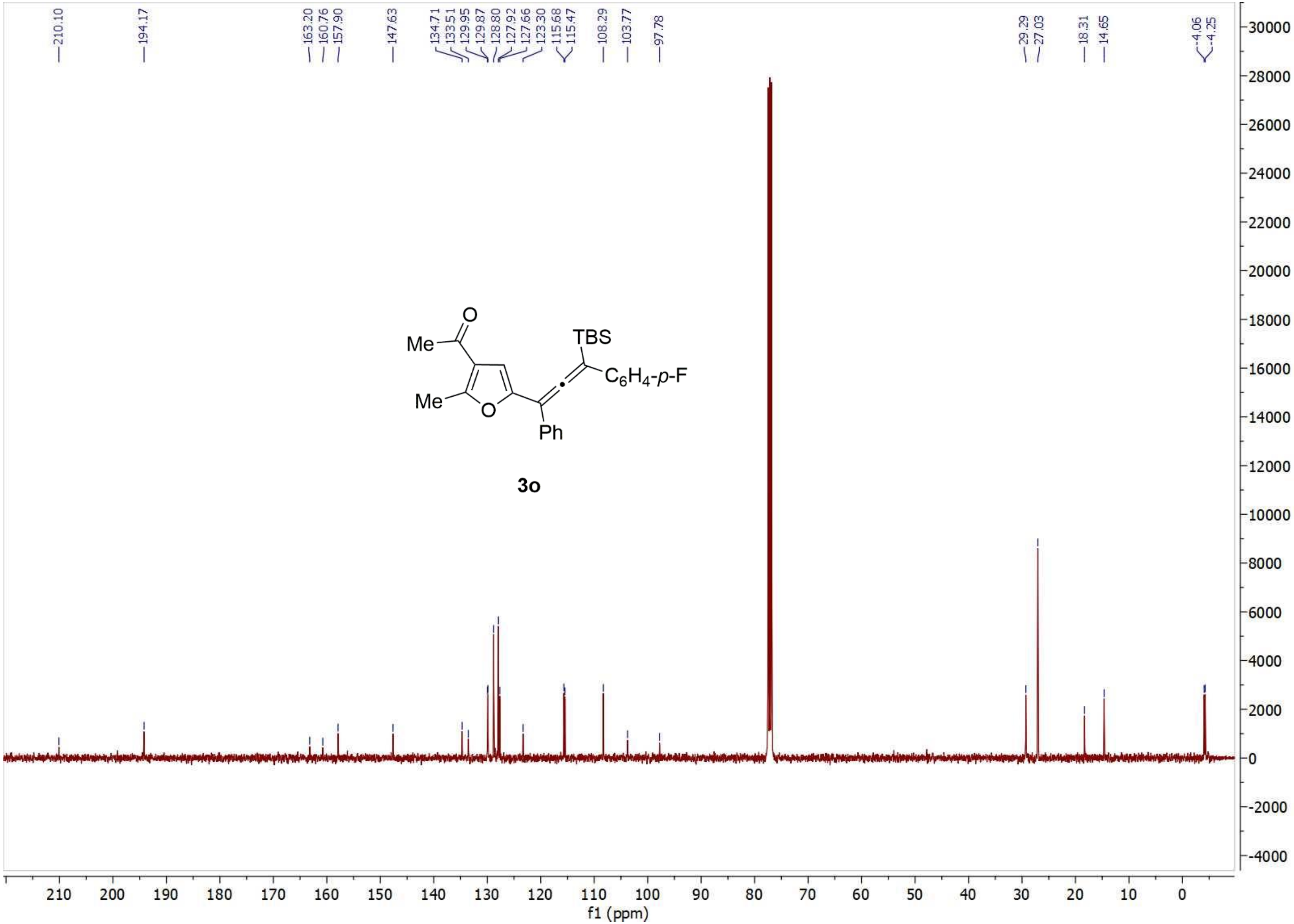

DEPT ^{13}C NMR of compound 3o (75 MHz, $CDCl_3$)

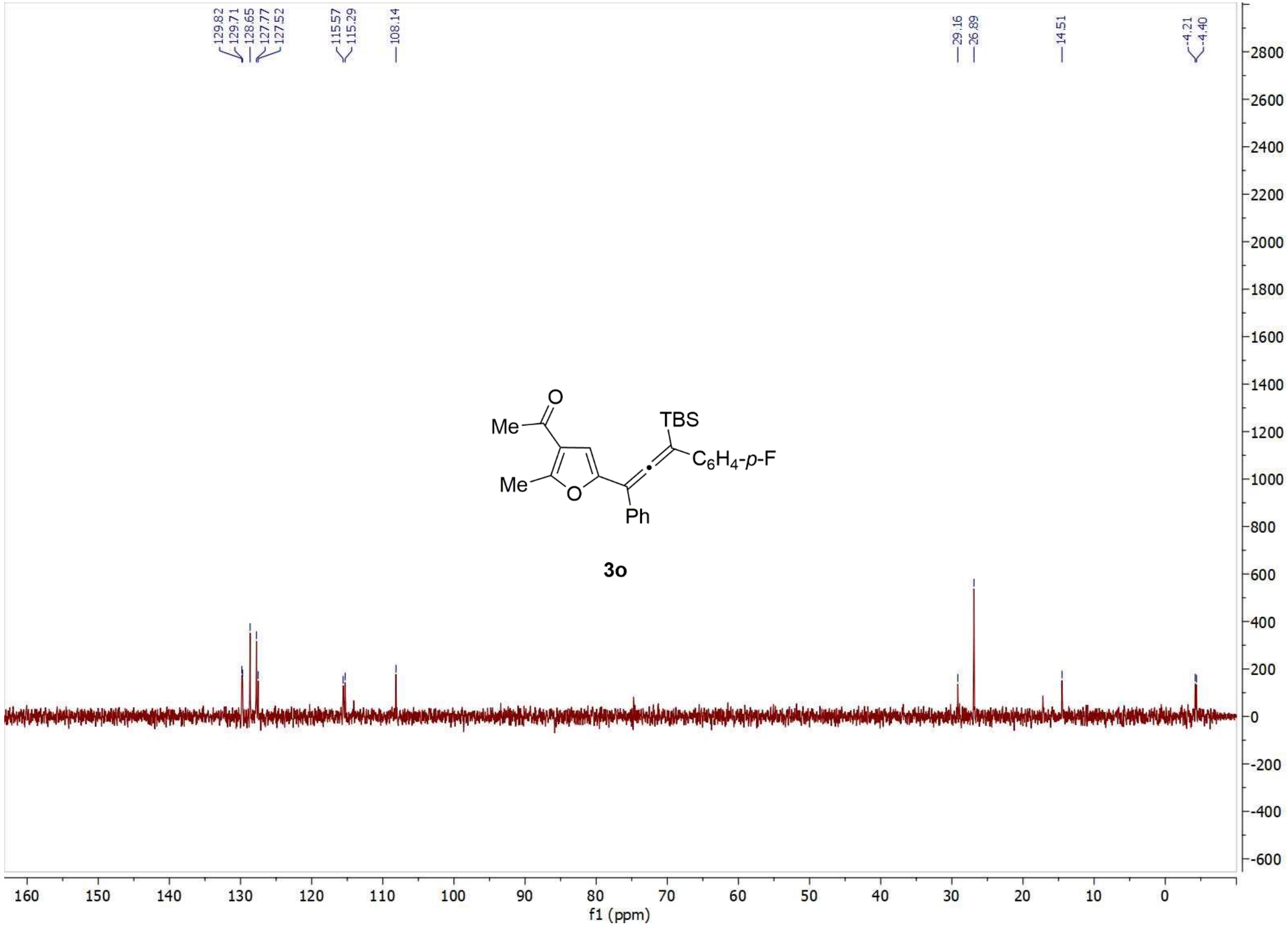

^{19}F NMR of compound 3o (282 MHz, $CDCl_3$)

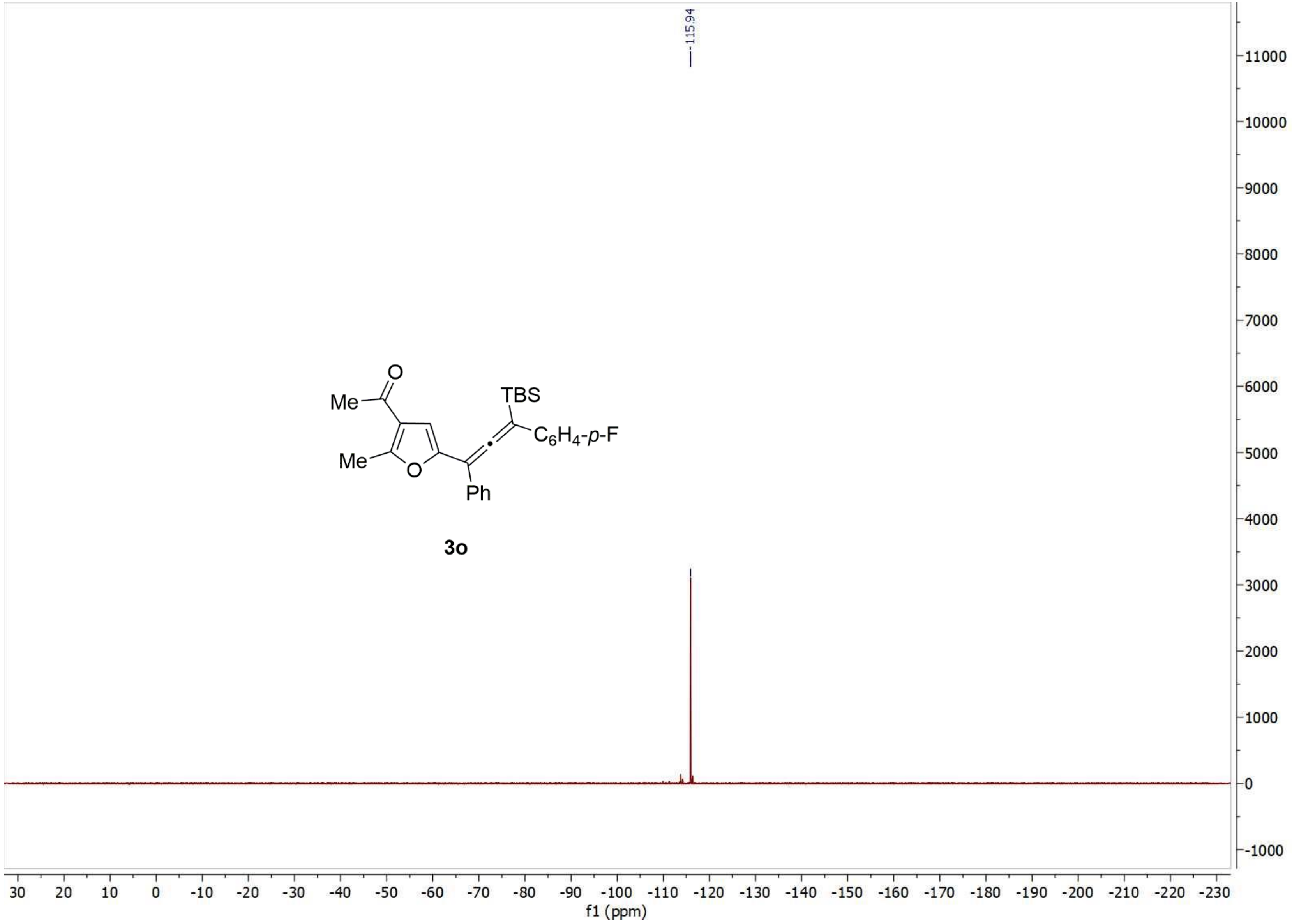

^{1}H NMR of compound 3p (300 MHz, $CDCl_3$)

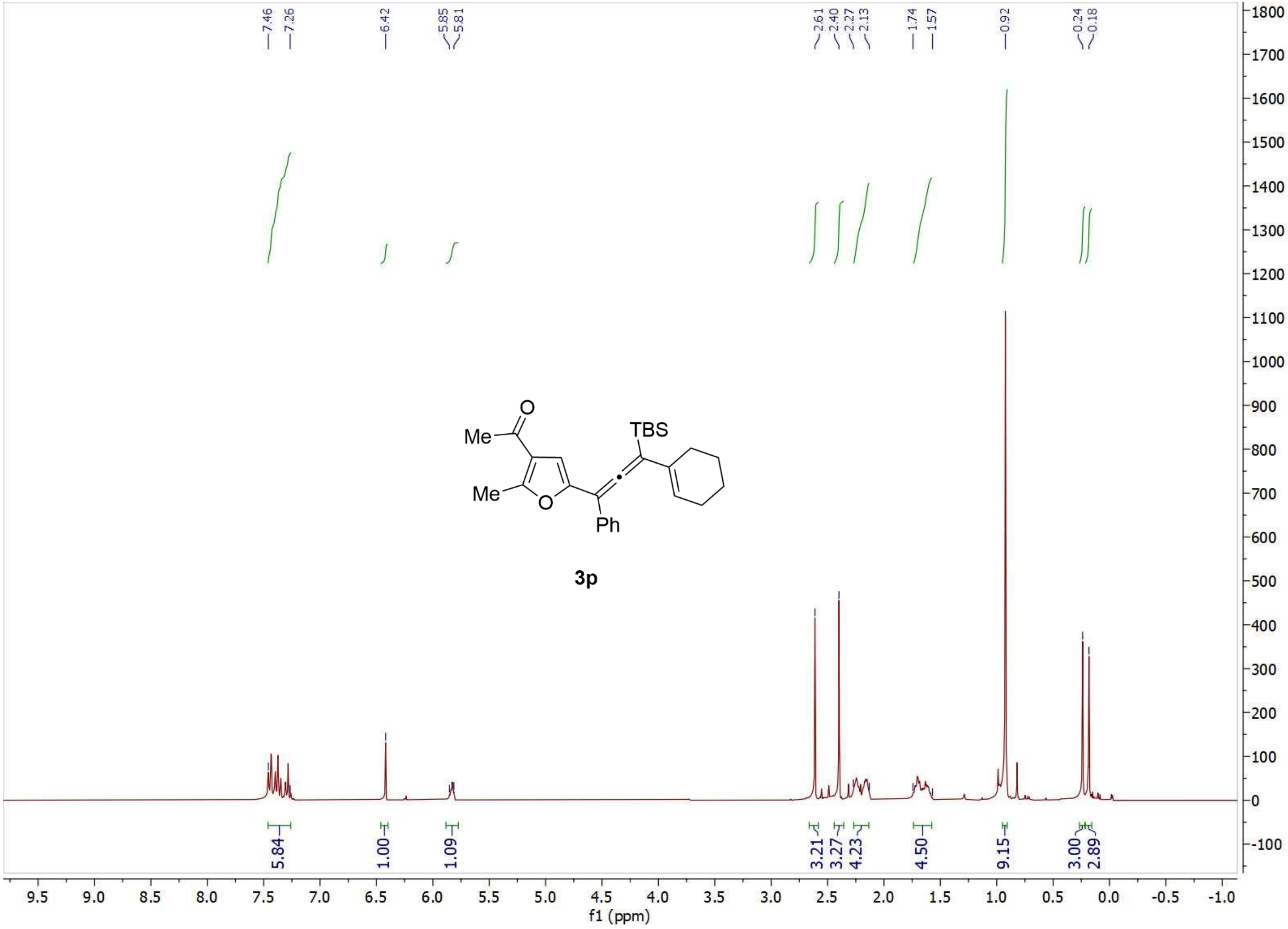

^{13}C NMR of compound 3p (75 MHz, $CDCl_3$)

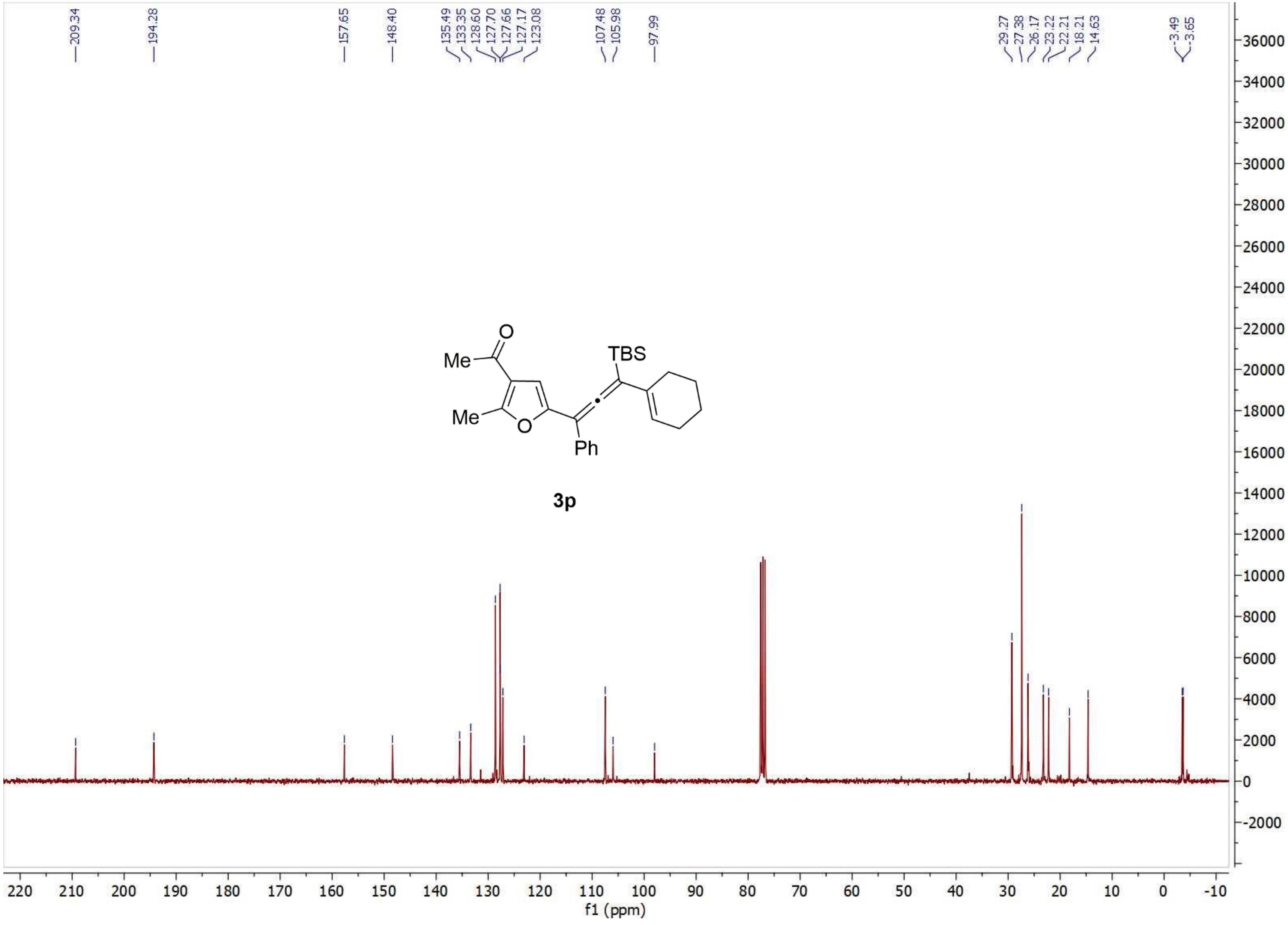

DEPT ^{13}C NMR of compound 3p (75 MHz, $CDCl_3$)

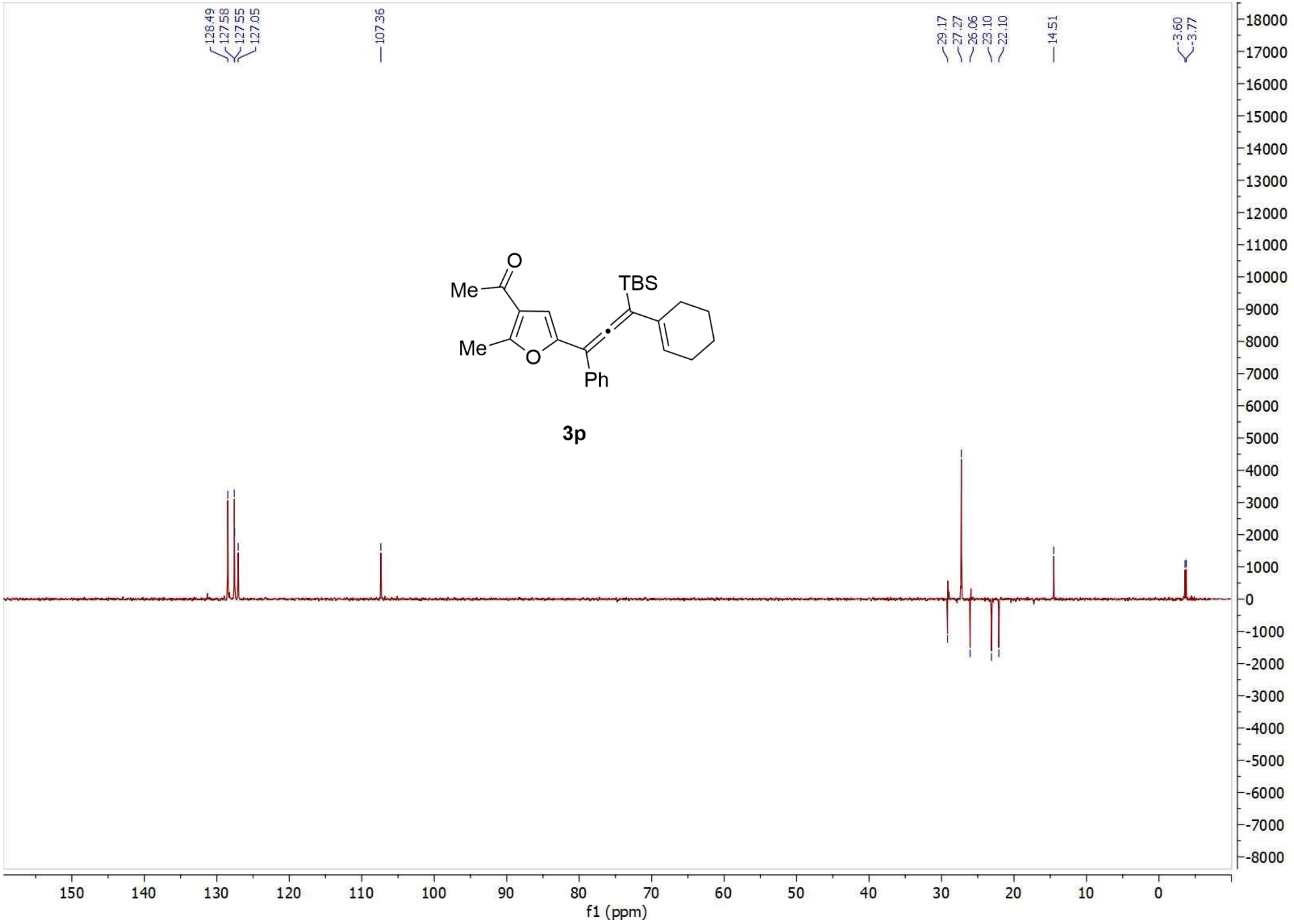

3p

^{1}H NMR of compound 3q (300 MHz, $CDCl_3$)

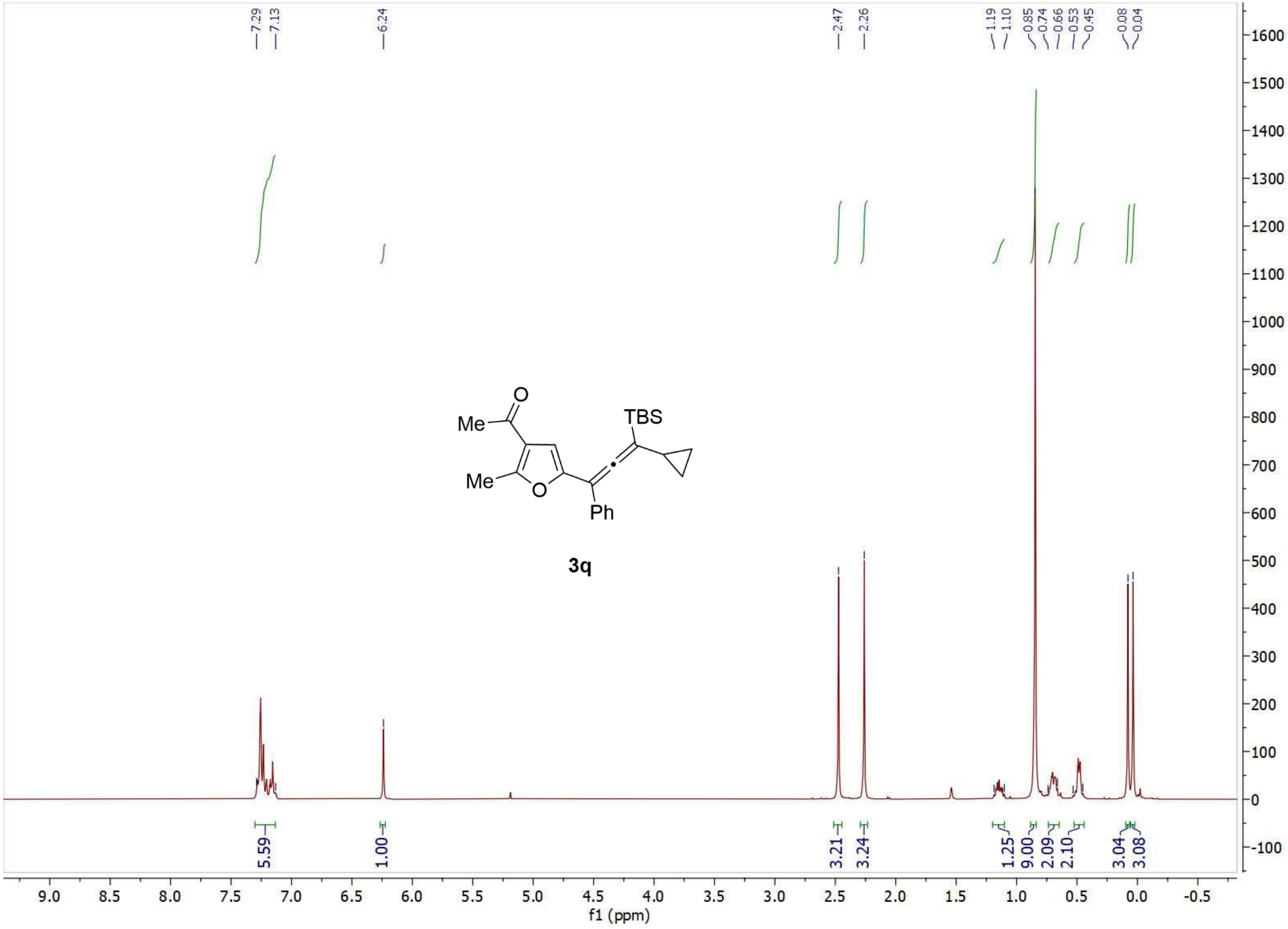

^{13}C NMR of compound 3q (75 MHz, $CDCl_3$)

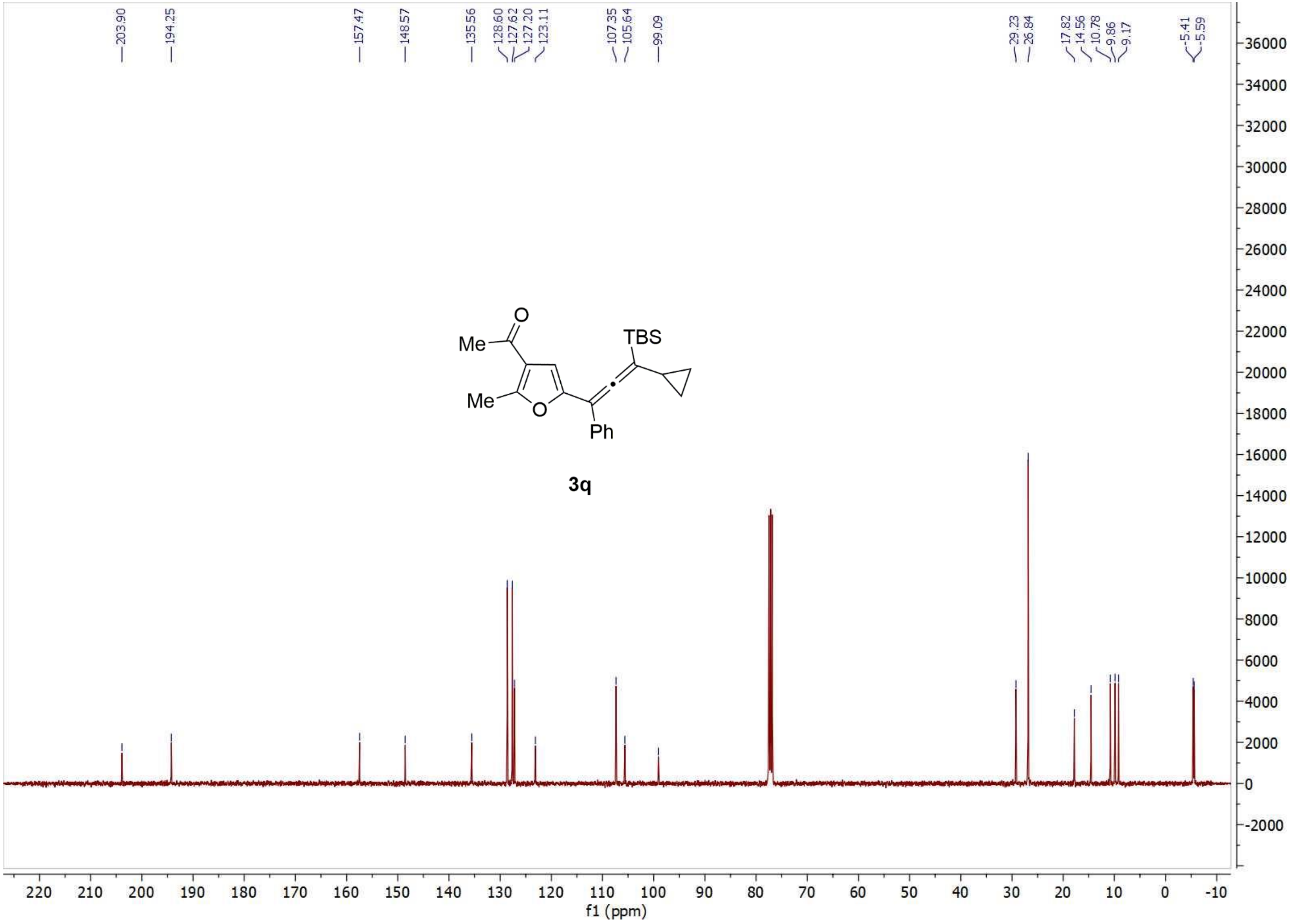

DEPT ^{13}C NMR of compound 3q (75 MHz, $CDCl_3$)

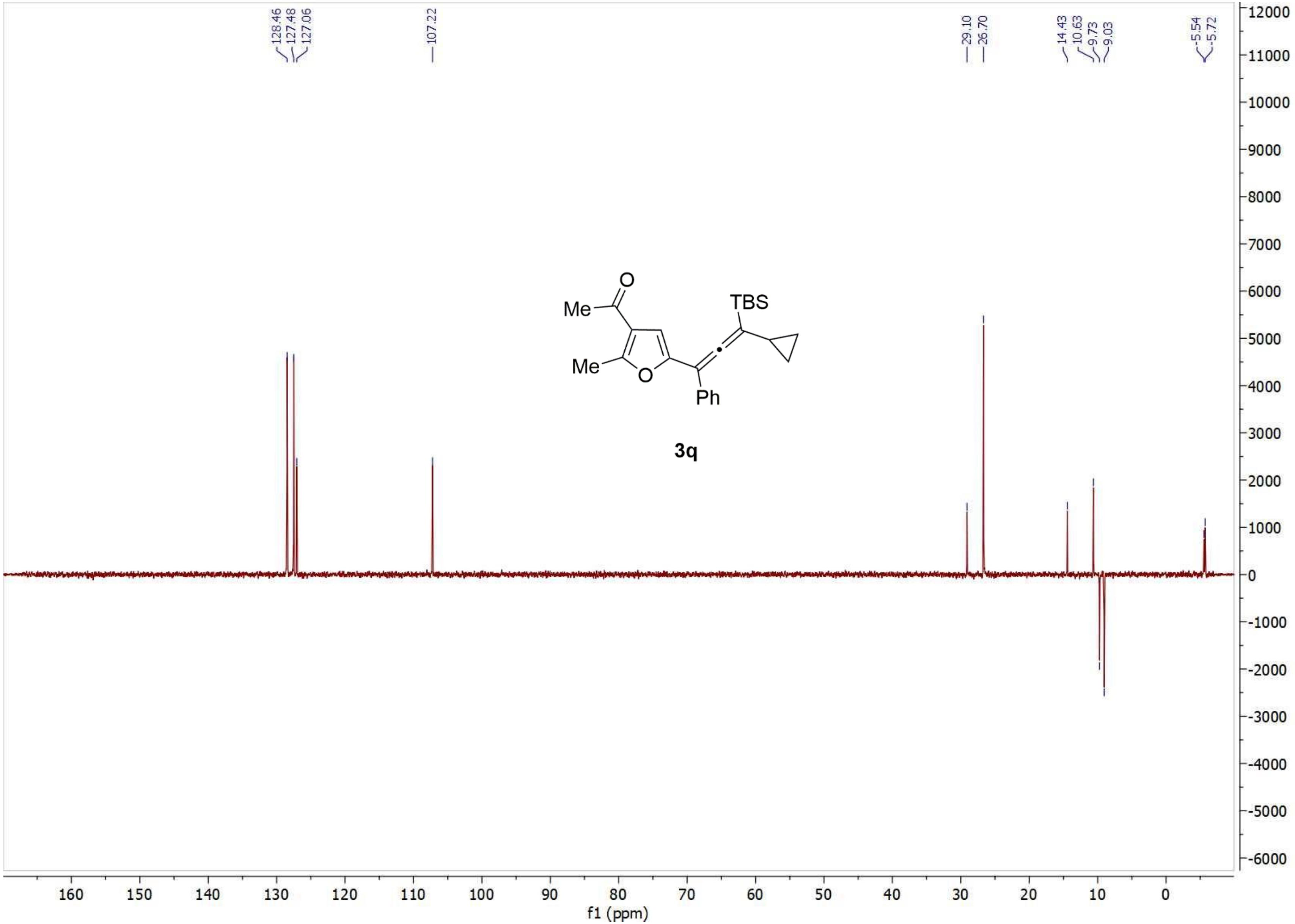

^{1}H NMR of compound 3r (300 MHz, $CDCl_3$)

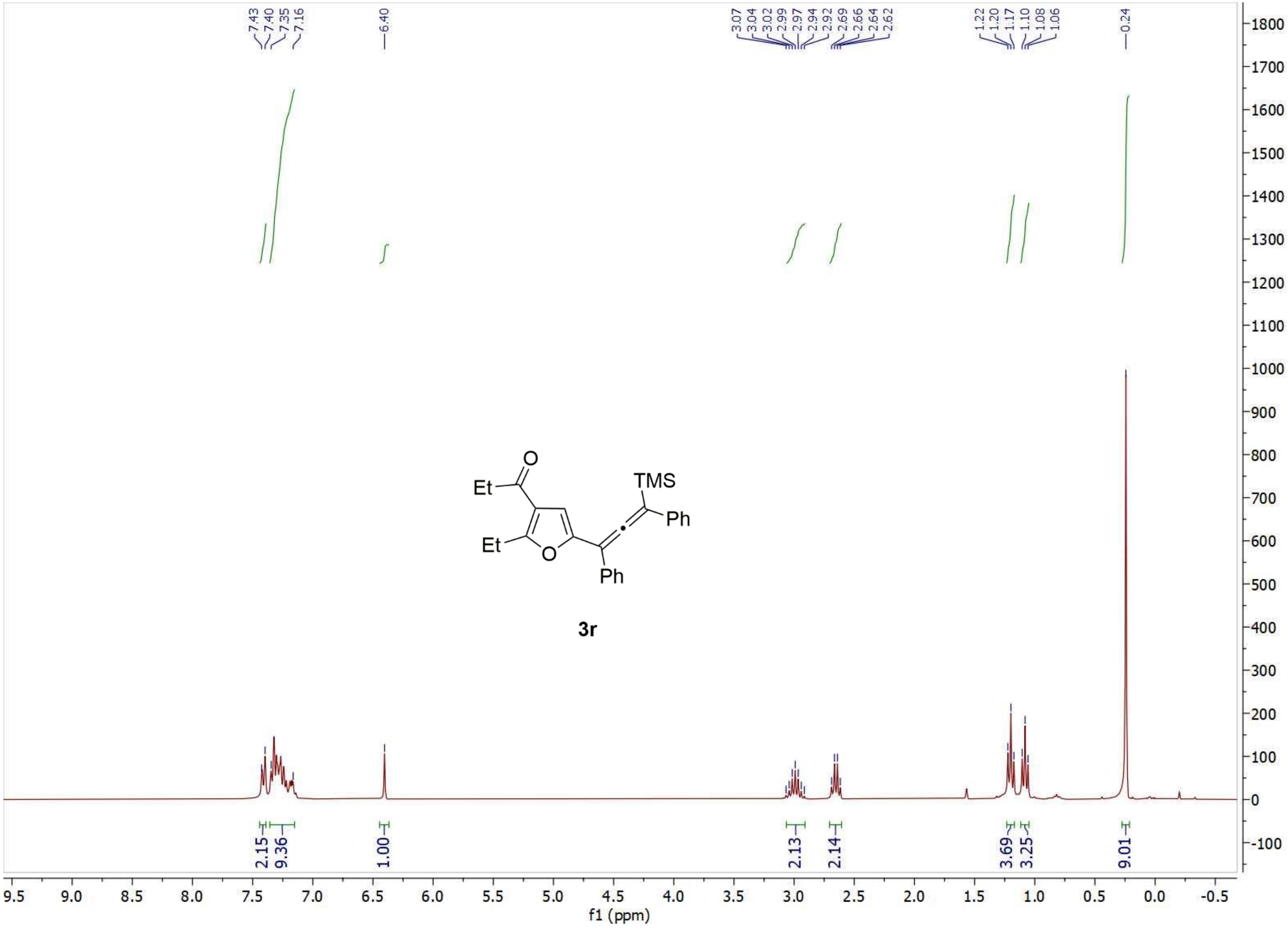

^{13}C NMR of compound 3r (75 MHz, $CDCl_3$)

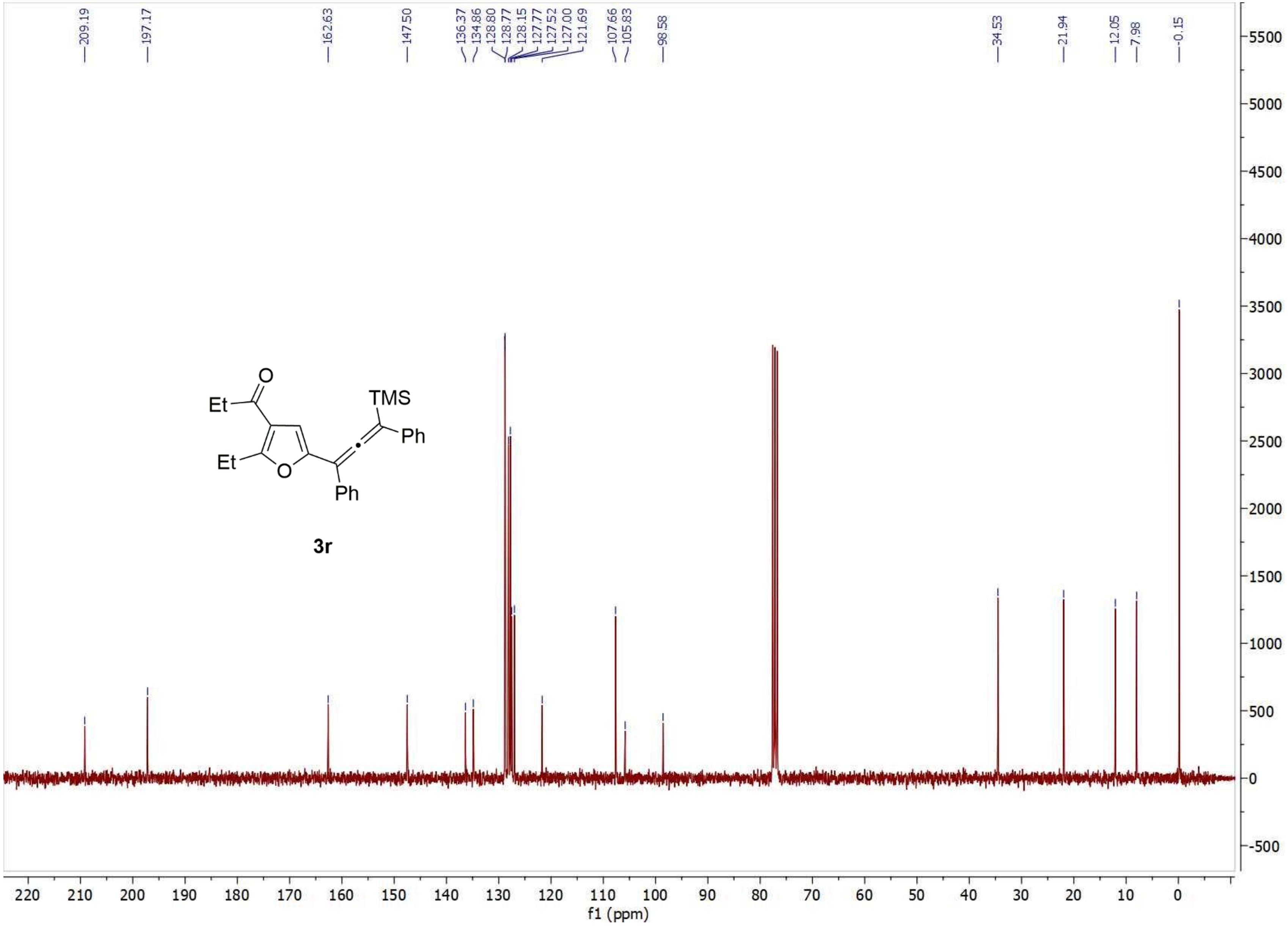

DEPT ^{13}C NMR of compound 3r (75 MHz, $CDCl_3$)

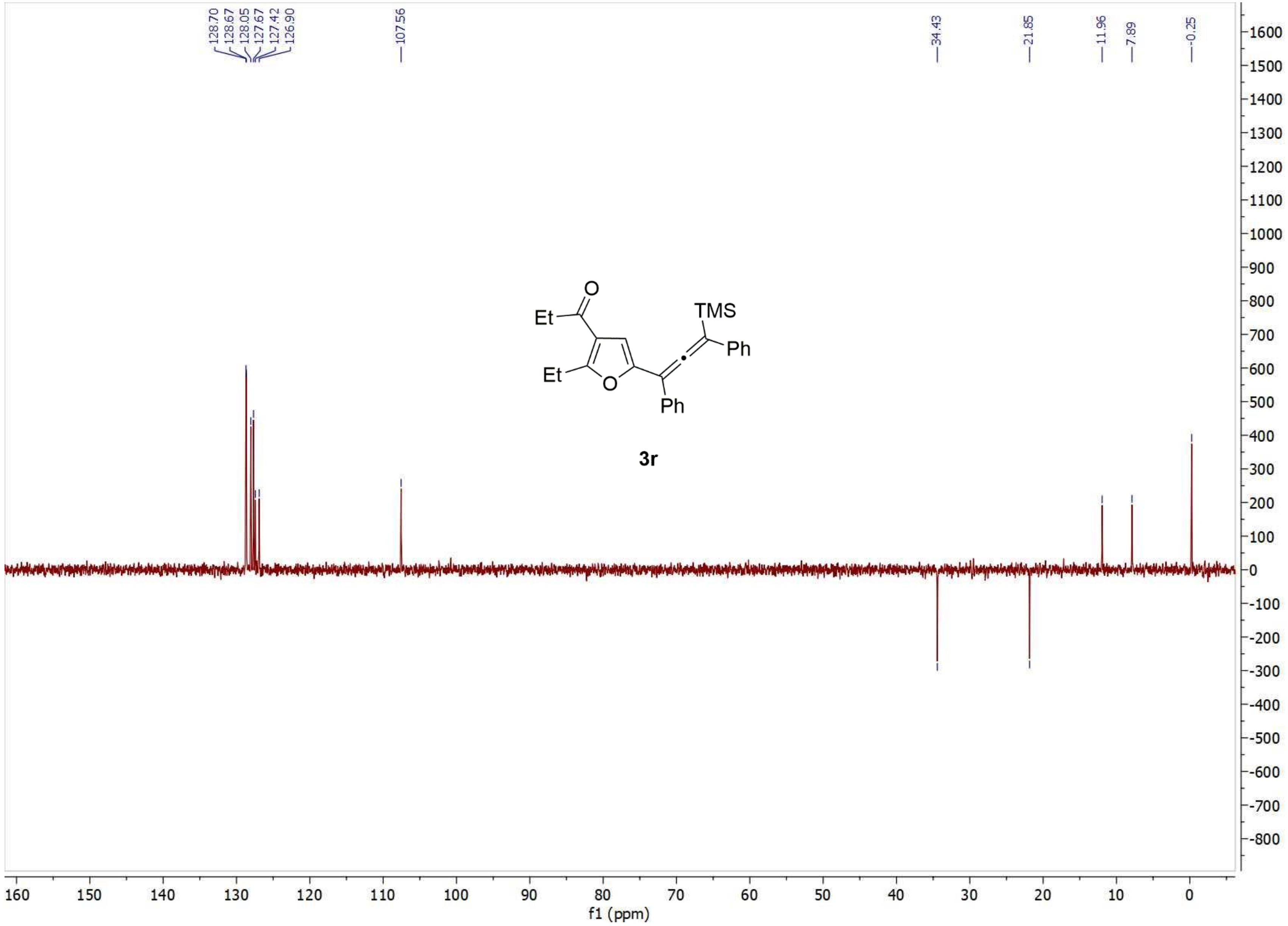

^{1}H NMR of compound 3s (300 MHz, $CDCl_3$)

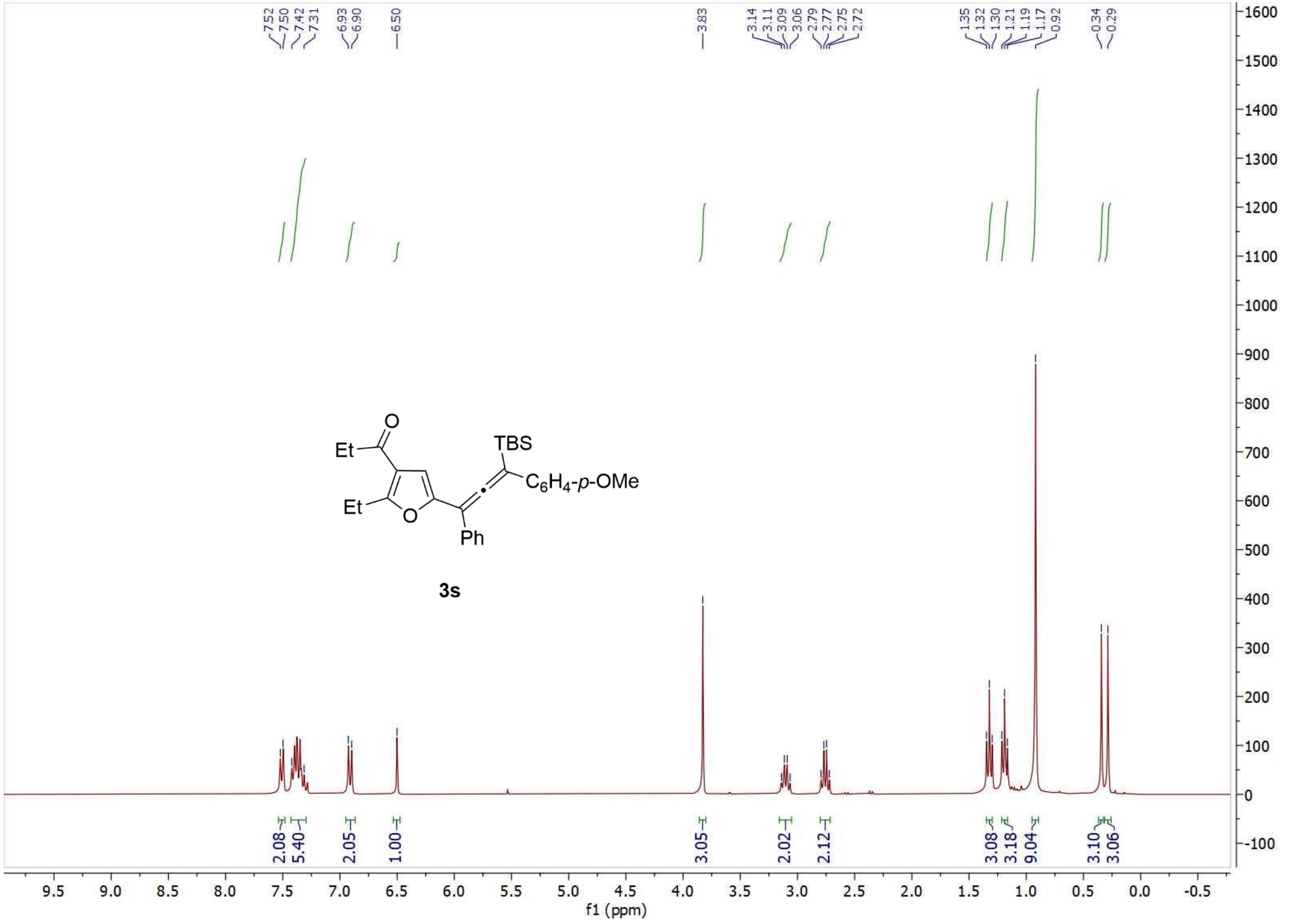

^{13}C NMR of compound 3s (75 MHz, $CDCl_3$)

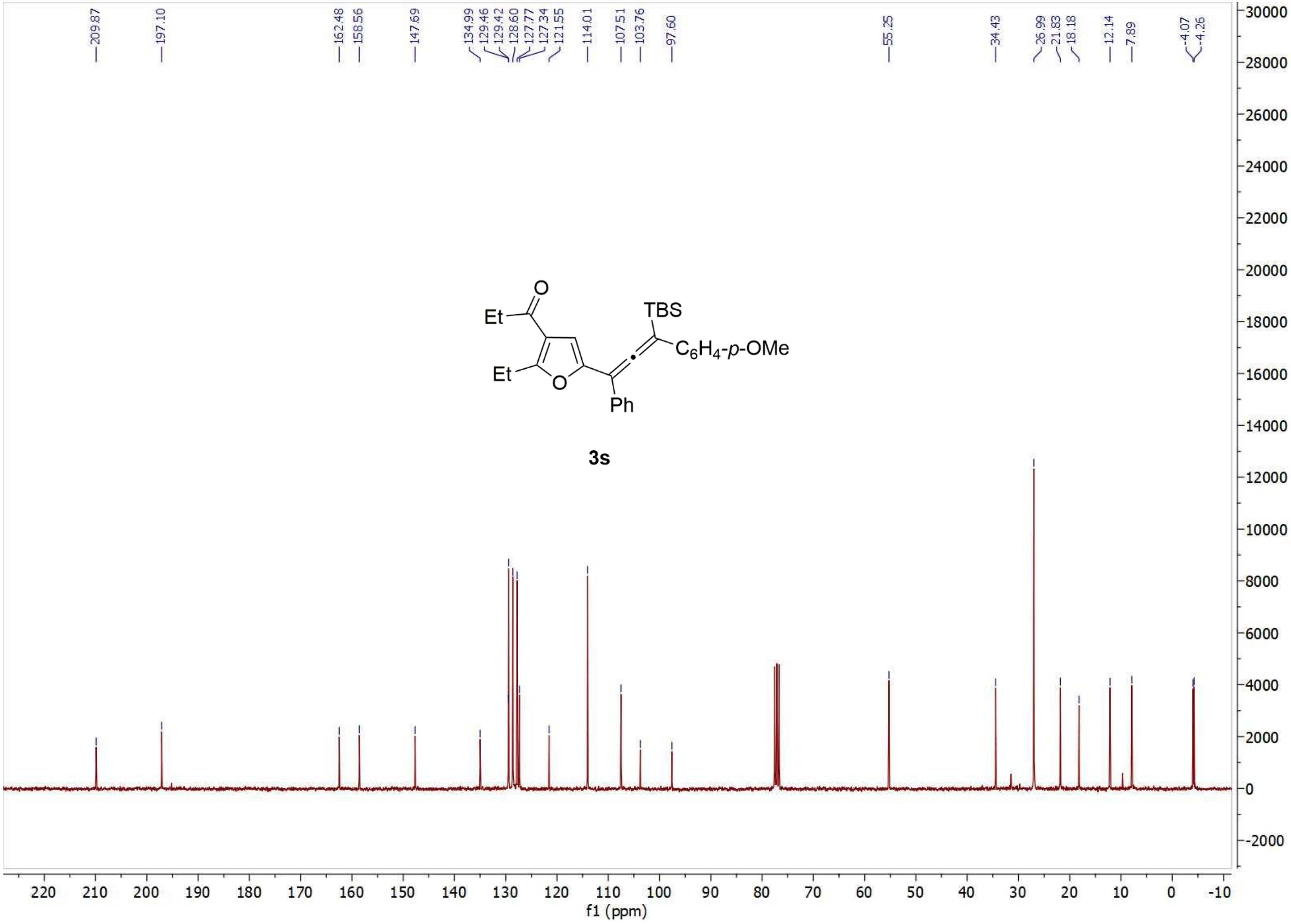

DEPT ^{13}C NMR of compound 3s (75 MHz, $CDCl_3$)

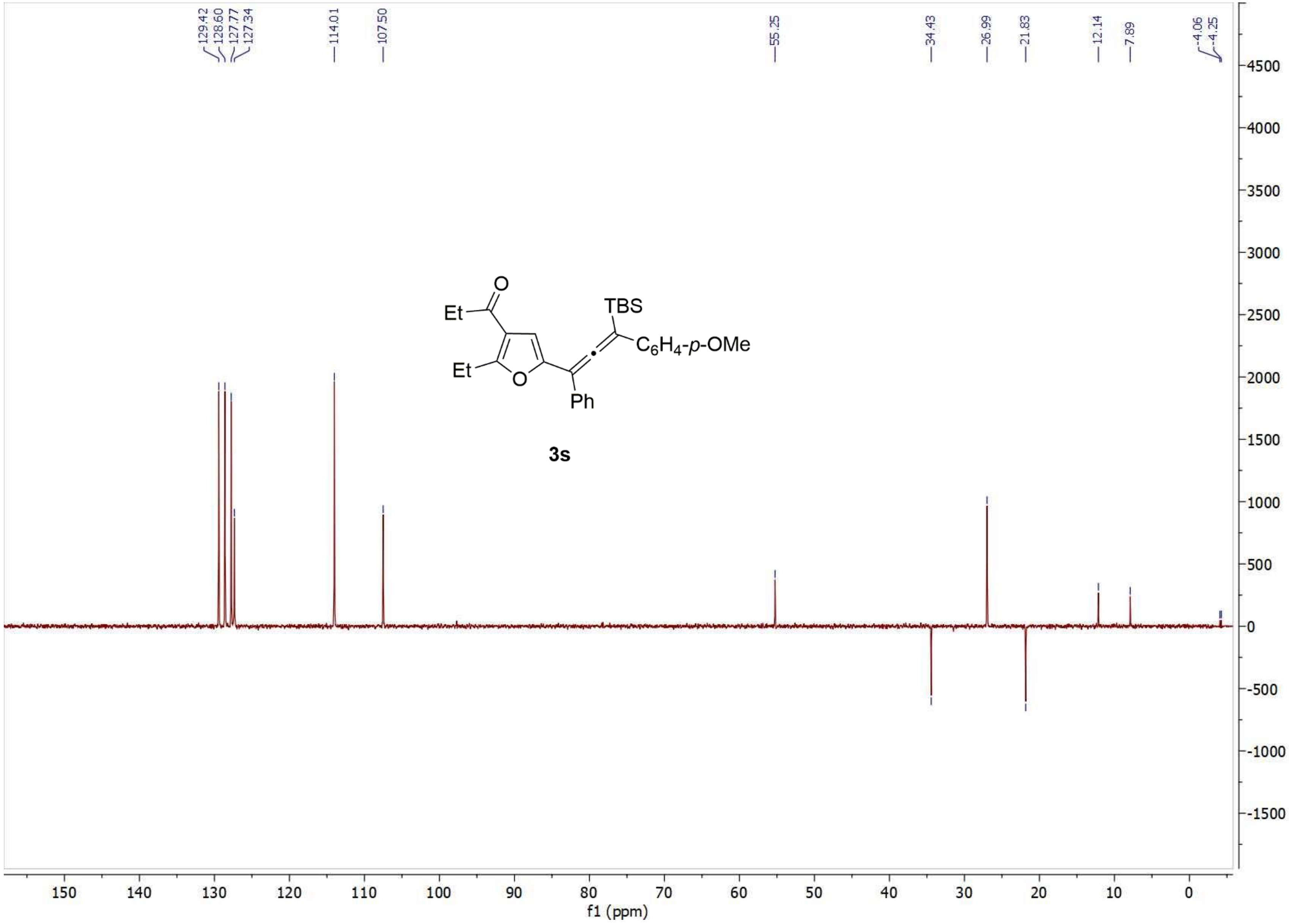

^{1}H NMR of compound 3t (300 MHz, $CDCl_3$)

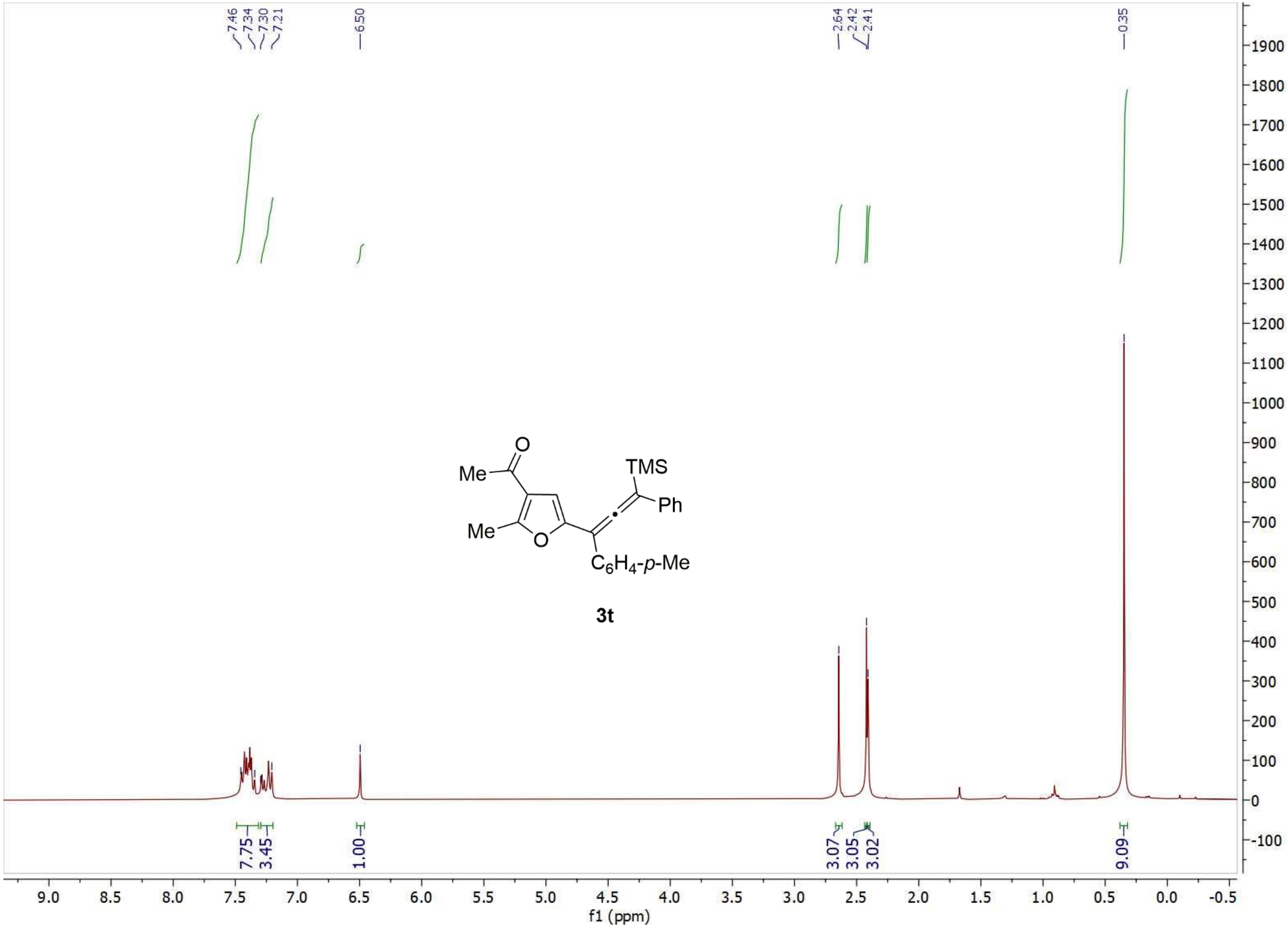

^{13}C NMR of compound 3t (75 MHz, $CDCl_3$)

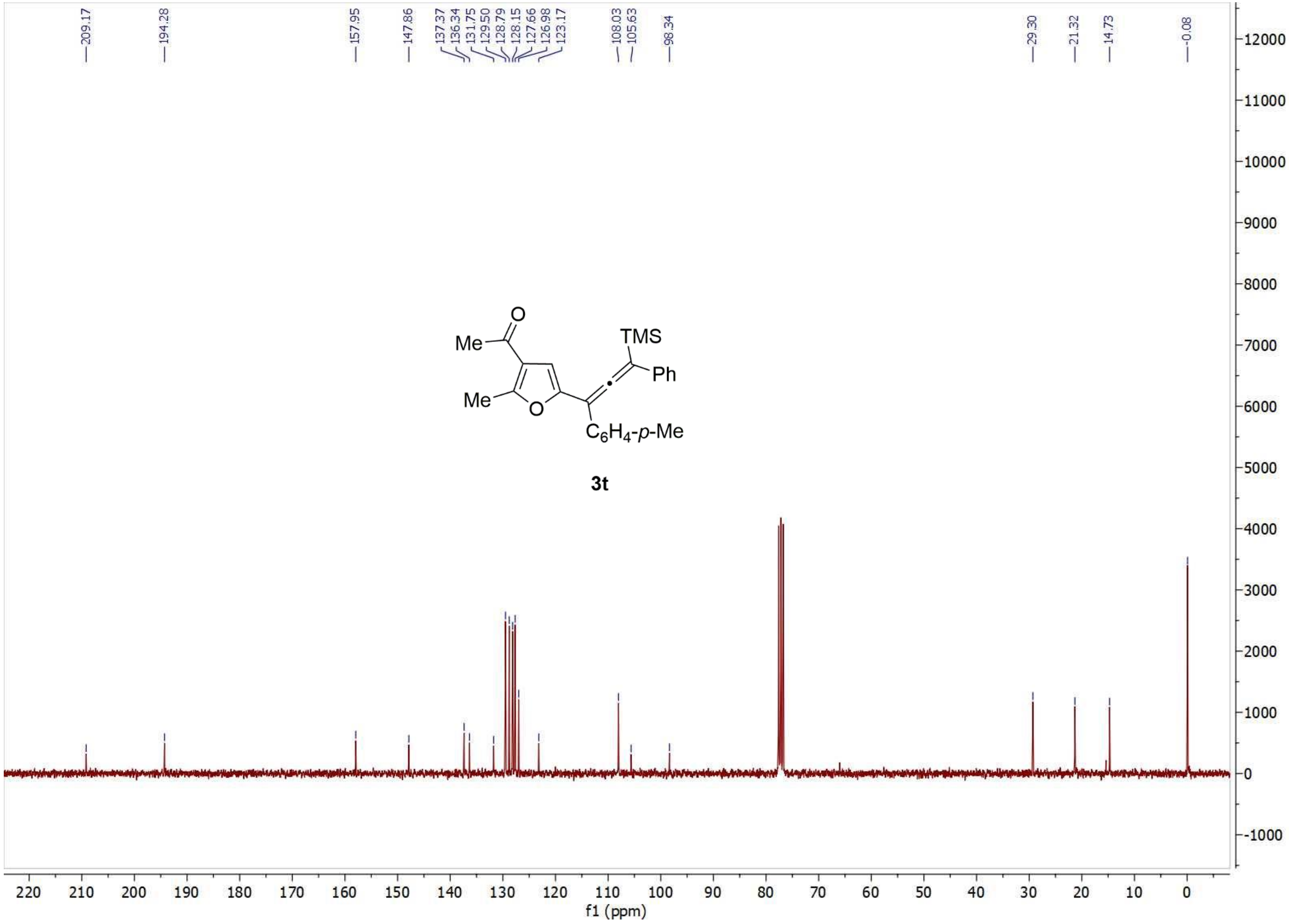

DEPT ^{13}C NMR of compound 3t (75 MHz, $CDCl_3$)

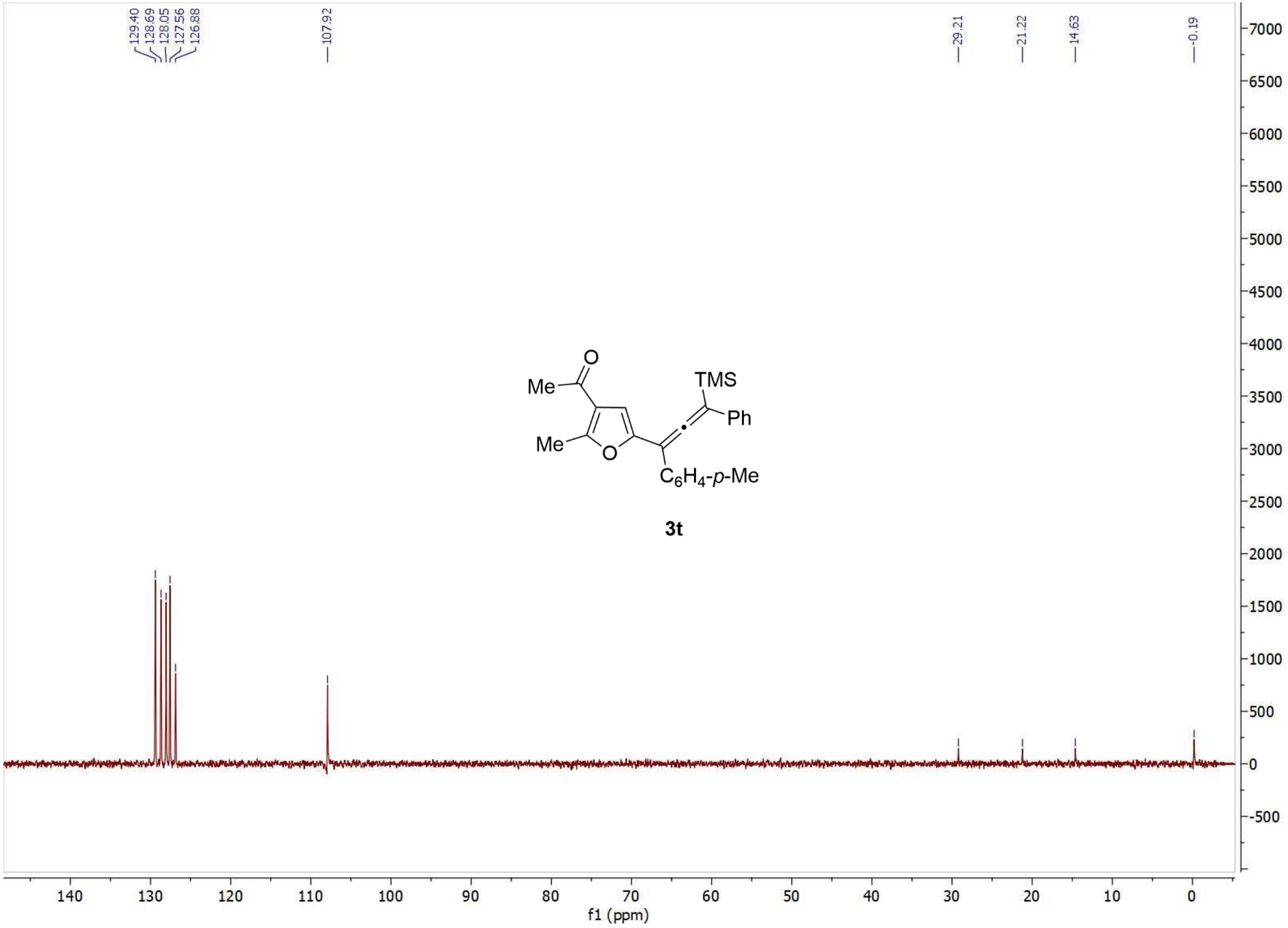

^{1}H NMR of compound 3u (300 MHz, $CDCl_3$)

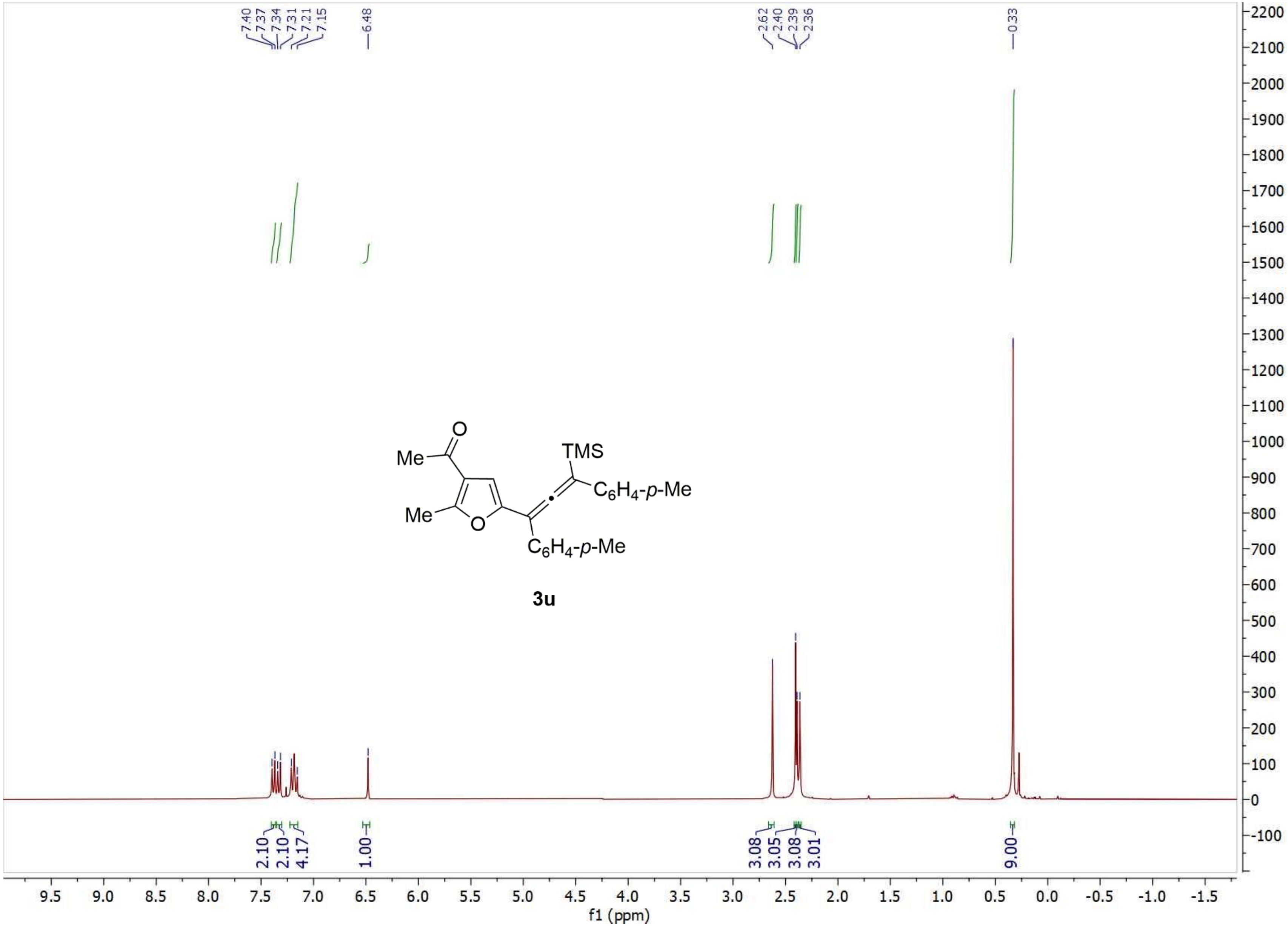

^{13}C NMR of compound 3u (75 MHz, $CDCl_3$)

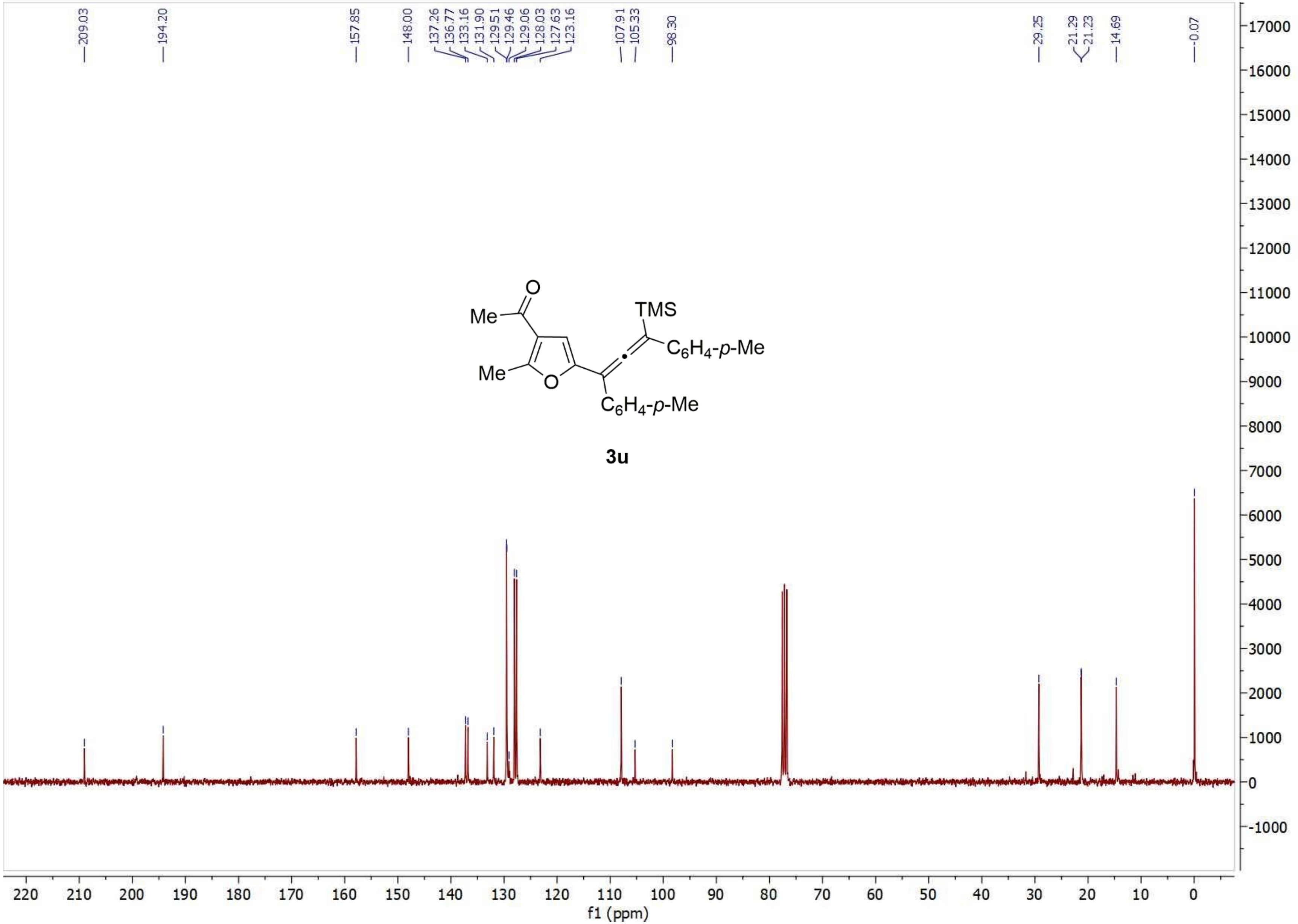

DEPT ^{13}C NMR of compound 3u (75 MHz, $CDCl_3$)

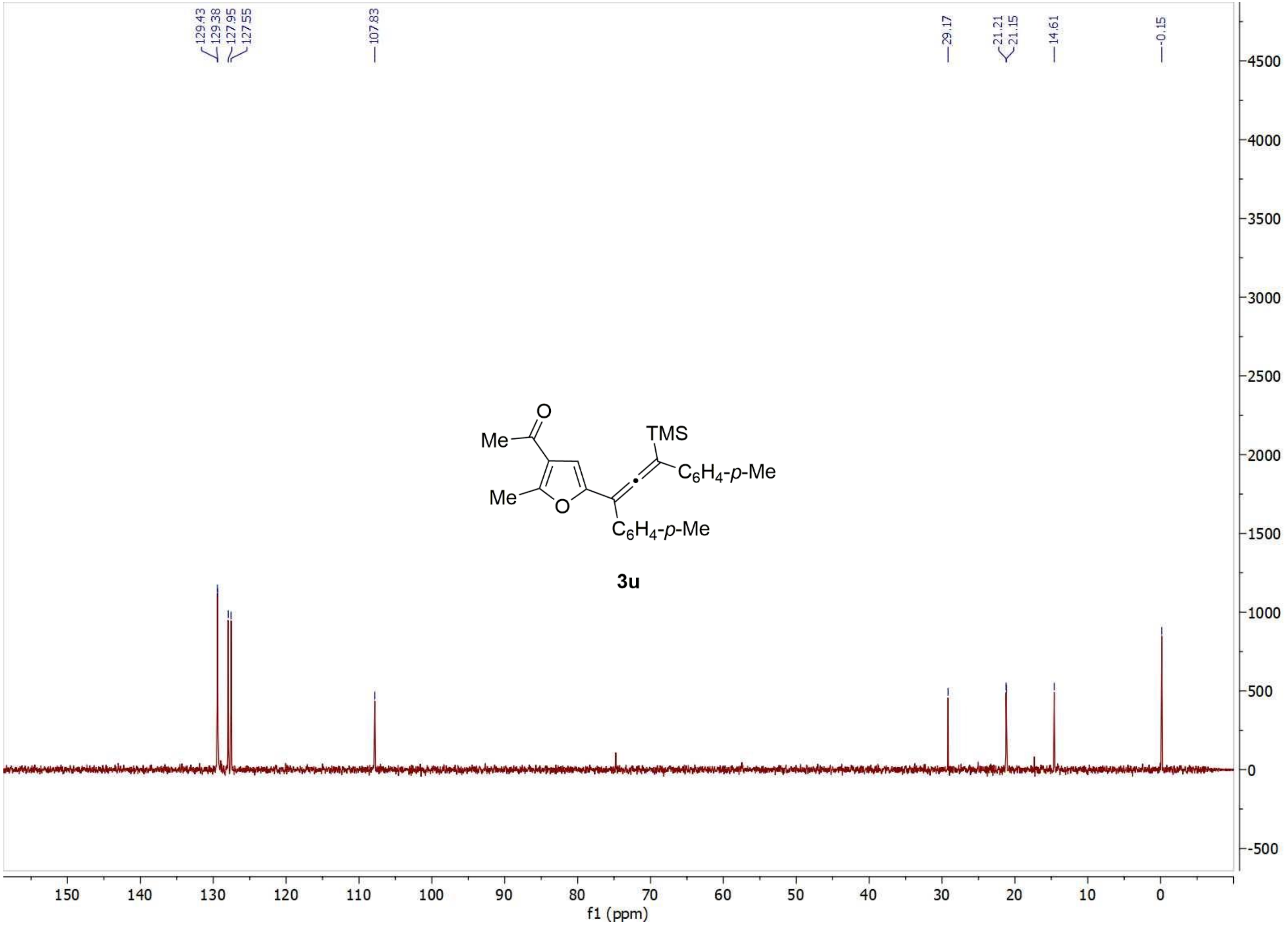

^{1}H NMR of compound 3v (300 MHz, $CDCl_3$)

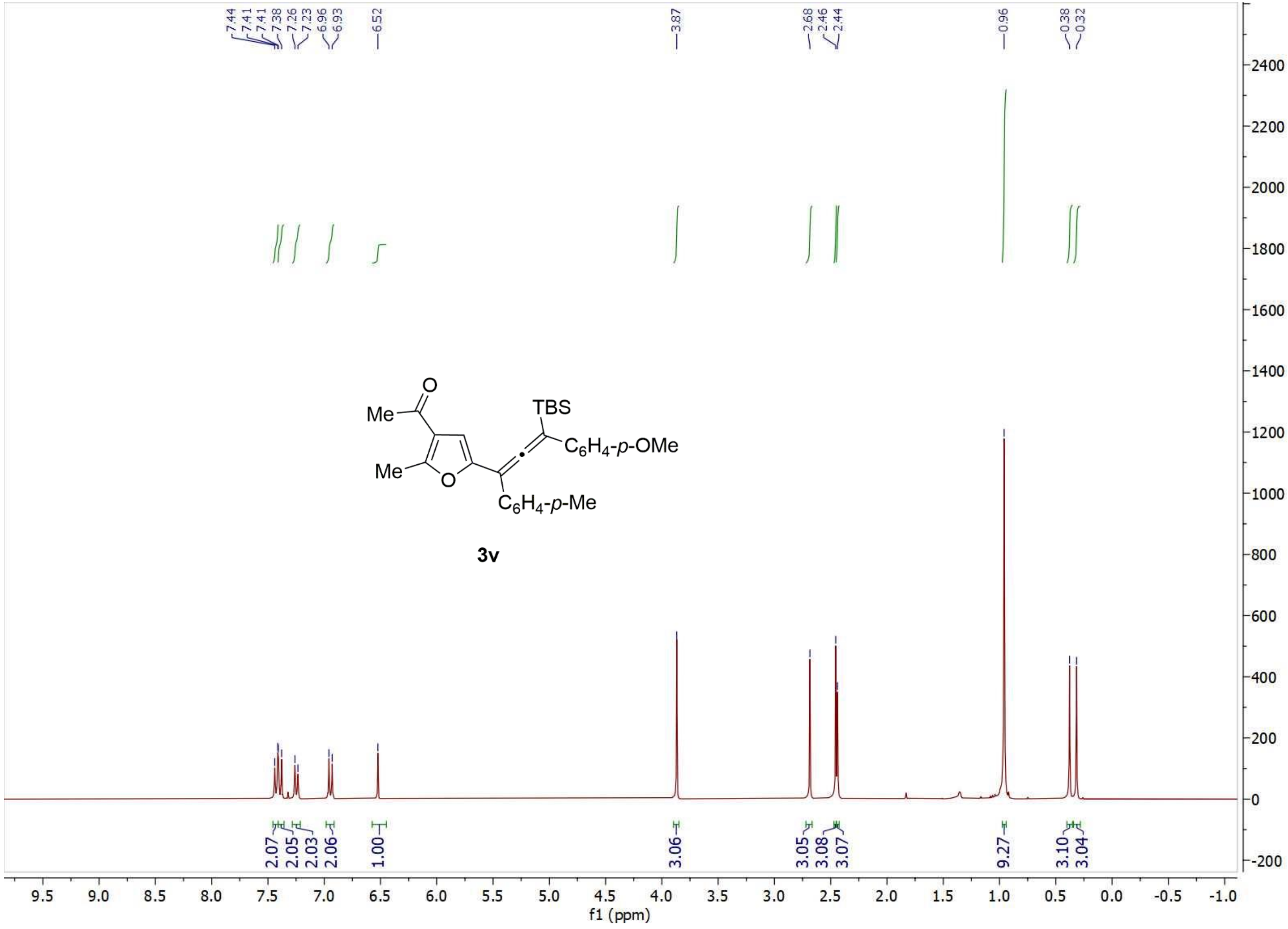

^{13}C NMR of compound 3v (75 MHz, $CDCl_3$)

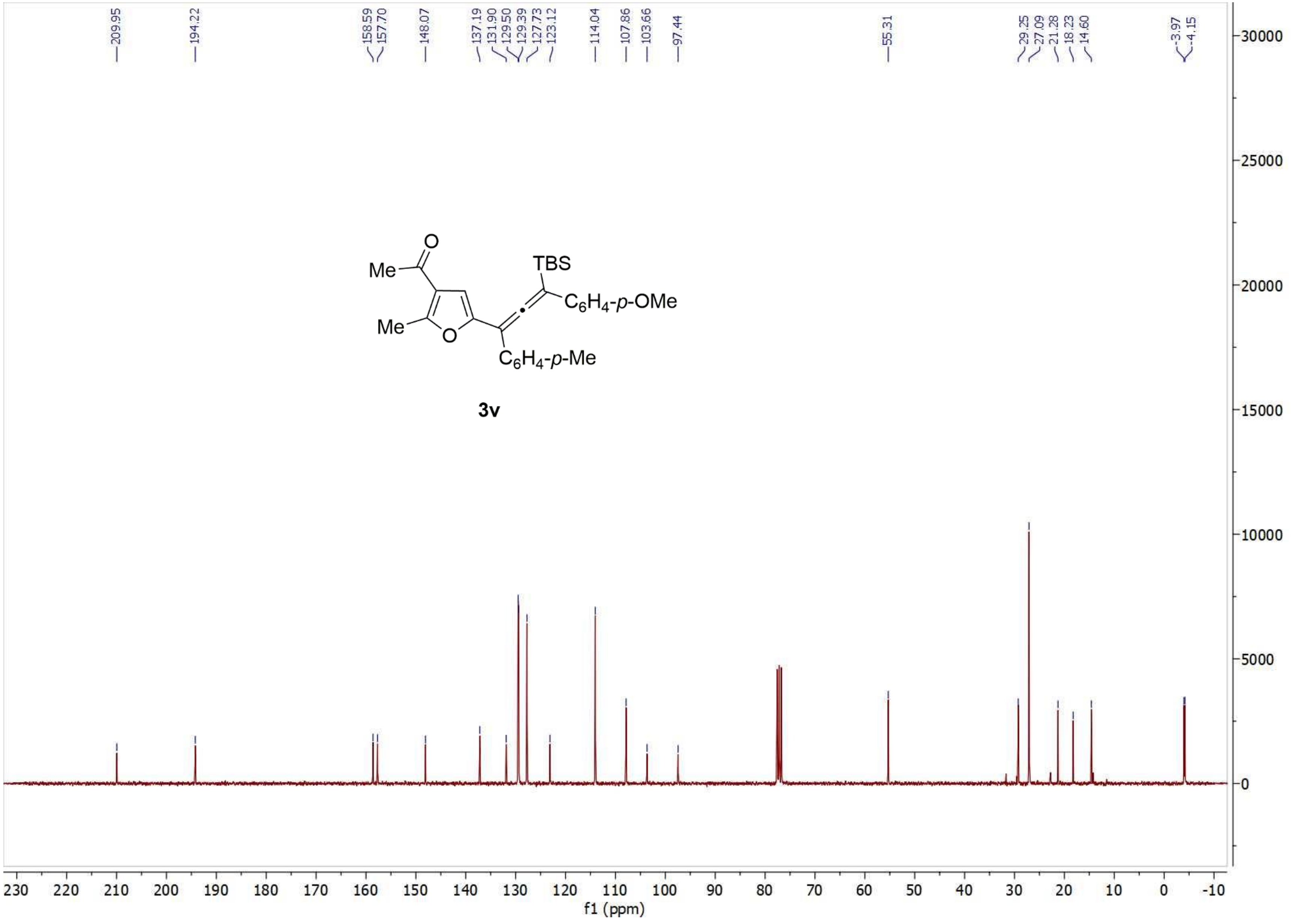

DEPT ^{13}C NMR of compound 3v (75 MHz, $CDCl_3$)

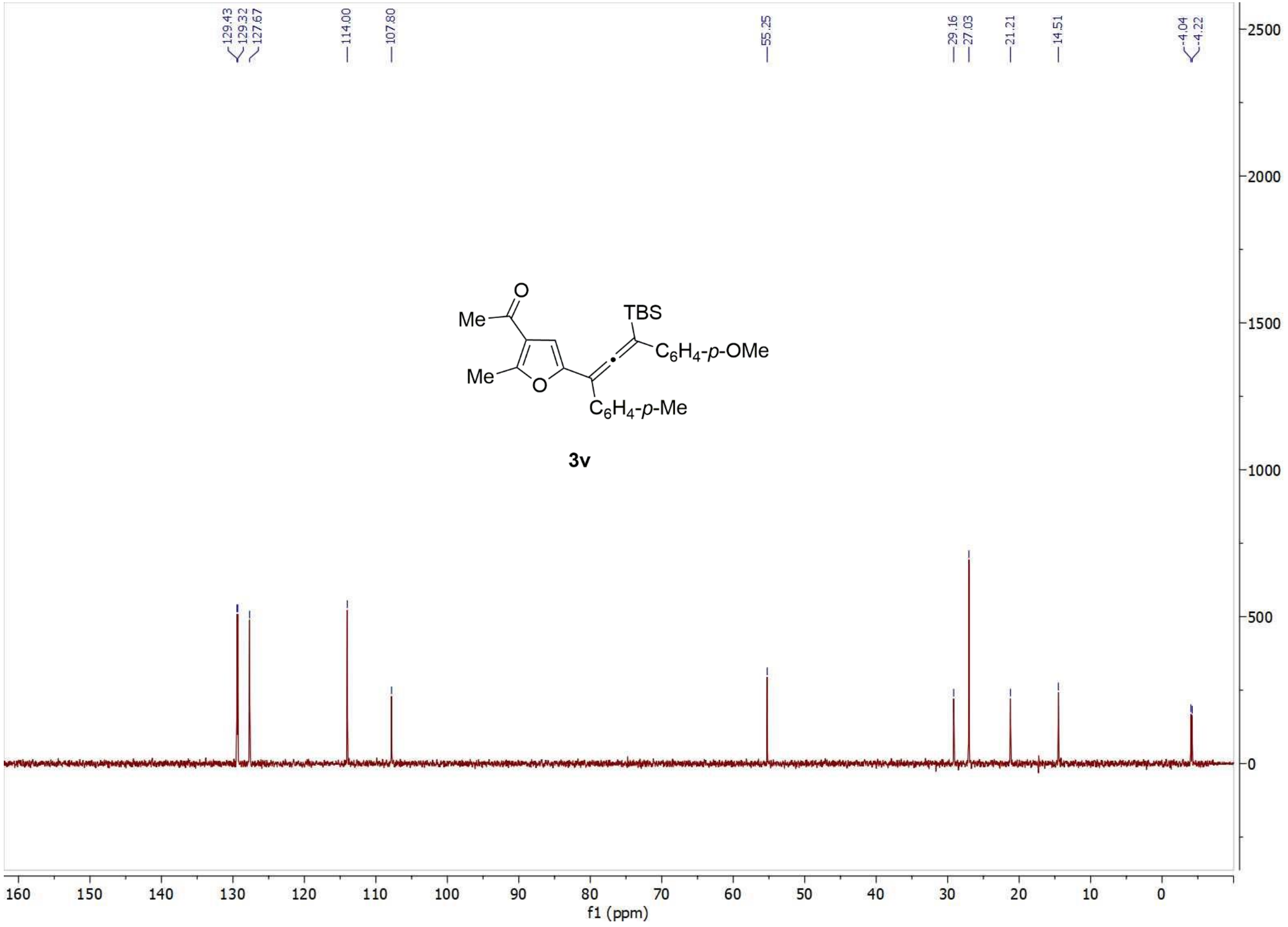

^{1}H NMR of compound 5 (300 MHz, $CDCl_3$)

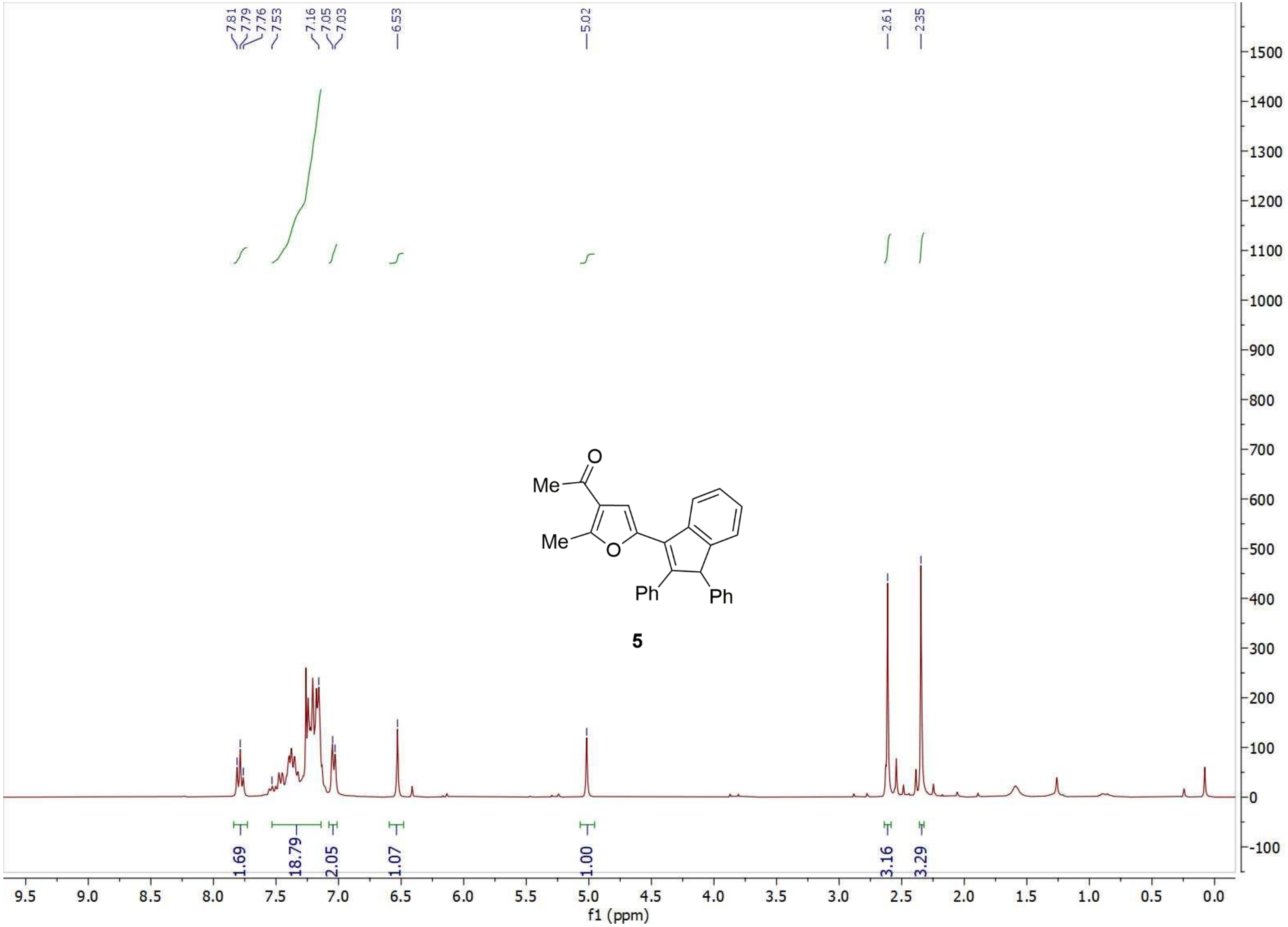

^{13}C NMR of compound 5 (75 MHz, $CDCl_3$)

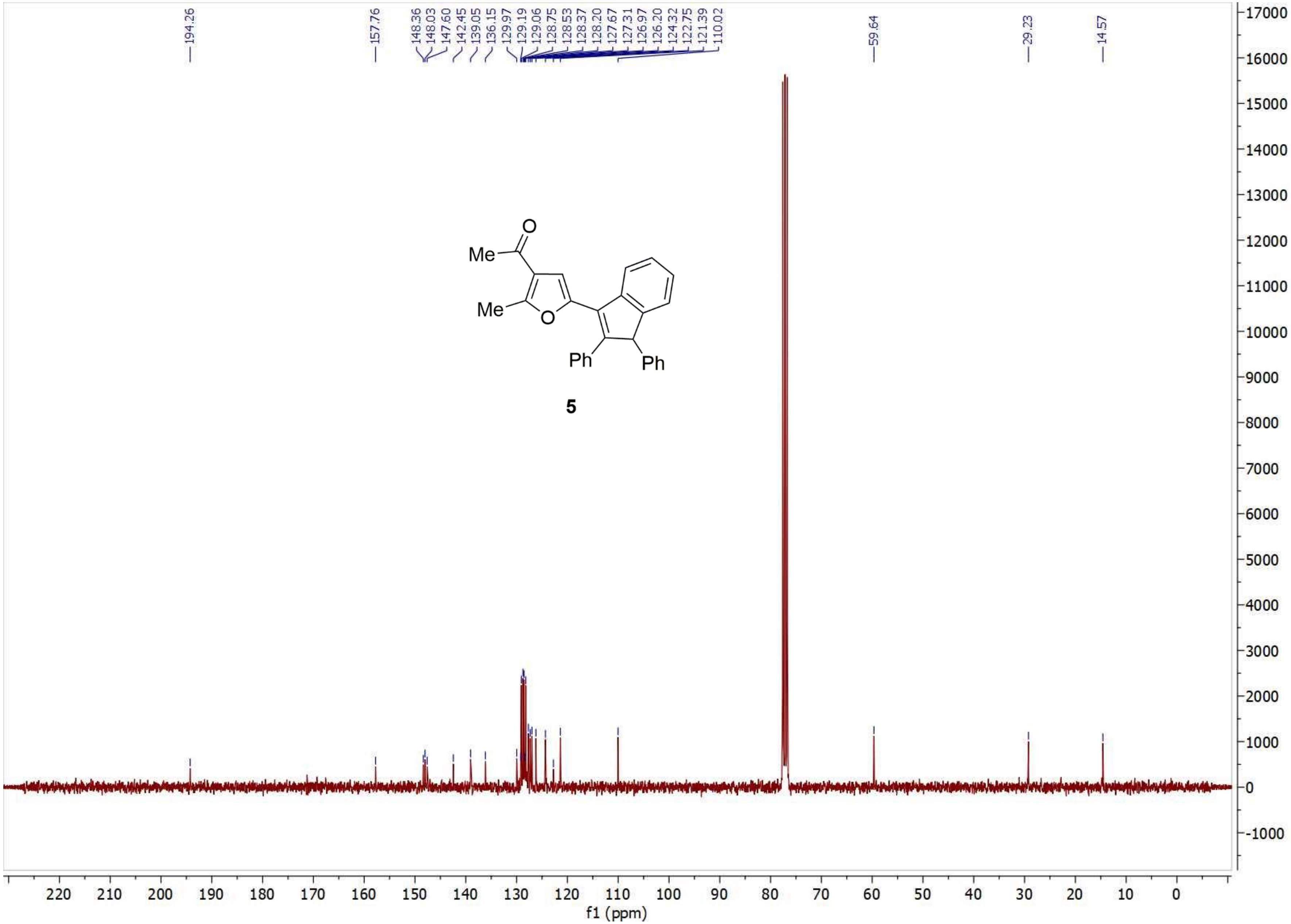

Supplement: Supplementary file 1 — ol4c01468_si_001.pdf [file ol4c01468_si_001.pdf]
